# Female Sexual Dysfunction: A Primer for Primary Care Health Professionals

**DOI:** 10.15766/mep_2374-8265.11312

**Published:** 2023-04-25

**Authors:** Sarah Merriam, Juliana M. Kling, Holly N. Thomas, Rachel S. Casas

**Affiliations:** 1 Clinical Assistant Professor, Department of Medicine, University of Pittsburgh School of Medicine, and Department of Medicine, VA Pittsburgh Healthcare System; 2 Associate Professor, Department of Medicine, Mayo Clinic; 3 Assistant Professor, Department of Medicine, University of Pittsburgh School of Medicine; 4 Associate Professor, Department of Medicine, Penn State Health Milton S. Hershey Medical Center

**Keywords:** Female Sexual Dysfunction, Human Sexuality, Primary Care, Women's Health

## Abstract

**Introduction:**

Female sexual dysfunction (FSD) is common and associated with decreased quality of life, relationship satisfaction, and overall well-being. However, primary care practitioners report discomfort discussing, diagnosing, and treating FSD.

**Methods:**

We delivered two sessions on the approach to evaluation and treatment of FSD: a 60-minute didactic session and a 90-minute workshop. The intended audience was primary health care professionals who care for women. The workshop utilized interactive teaching methods including a large-group discussion, case-based discussions, debrief of an observed patient-physician discussion, and language drills to develop participants’ knowledge and skills. Participants were surveyed about their practice patterns and attitudes toward FSD following the sessions on a 5-point Likert scale (1 = *strongly disagree,* 5 = *strongly agree*).

**Results:**

We collected 131 evaluations from a national Veterans Health Administration 60-minute didactic and four evaluations from the Society of General Internal Medicine Annual Meeting 90-minute workshop (response rates were 60% and 15%, respectively). One hundred thirty-five interdisciplinary trainees and practitioners from both audiences highly rated the workshop content (*M* = 4.1) and the overall session (*M* = 4.3). Didactic participants (*n* = 131) also reported high satisfaction (*M* = 4.5), increased knowledge and skills (*M* = 4.4), and improved interprofessional collaborative practice (*M* = 4.4) as a result of the training.

**Discussion:**

Our evaluation shows high satisfaction following interactive multimodal sessions on FSD. These adaptable resources can be used in multiple educational settings (didactic and workshop) and for multiple time frames to teach about FSD.

## Educational Objectives

By the end of this activity, learners will be able to:

1. Outline a patient-centered approach to evaluating female sexual dysfunction (FSD).
2. Identify biopsychosocial factors, medical conditions, and medications that can contribute to FSD.
3. Formulate a diagnostic plan for a patient with FSD based upon presenting history and symptoms.
4. Discuss the currently available treatment options for different types of FSD.

## Introduction

Sexual function is linked to overall well-being, quality of life, and relationship satisfaction in women.^1–3^ Sexual problems are common and are more prevalent among women compared to men.^4^ Female sexual dysfunction (FSD), defined by chronic sexual problems causing clinical distress, affects approximately one in eight women across the life span.^3,4^ The cause of FSD is often a complex interplay of health, interpersonal, sociocultural, and psychological factors.^5,6^

Though willing to discuss sexual problems with clinicians, patients are often hesitant to initiate the conversation. Patients’ barriers to discussing sexual function include fear of embarrassing their physicians or worry that their concerns will be dismissed.^7–9^ Additionally, physicians rarely screen for FSD or initiate conversations about sexual function.^7,8,10,11^ Clinician barriers to discussing sexual function include feelings of embarrassment, uncertainty about how to initiate discussions, and lack of knowledge and training with respect to treatment options.^12,13^ Best practices for treatment of FSD often involve a multidisciplinary team approach comprising nonpharmacologic and pharmacologic modalities. However, practitioners may lack familiarity with or access to treatments, many of which are newly approved or used off-label. A majority of trainees across diverse backgrounds recognize FSD as an important educational topic yet report insufficient exposure to it during training.^14^ Thus, FSD remains underidentified and undertreated in clinical practice.

Given the prevalence and impact of FSD, as well as the established limitations in knowledge and comfort regarding management of FSD among trainees and clinicians, there is a clear need for educational efforts in this domain. Despite this, few resources exist to equip primary health care professionals with the knowledge and skills to effectively diagnose and treat FSD.^15–19^ We searched for educational materials in the domain of FSD on PubMed and *MedEdPORTAL.* Most of the published educational content does not incorporate recently available pharmacologic therapies for FSD,^15–17,20^ focuses only on one aspect of treatment (e.g., diagnosis,^11,21^ treatment^18,22^), or is not open access.^19^ Furthermore, none of these materials are designed for live implementation (e.g., workshops) or include opportunity for either knowledge application or skills practice. While materials on general women's health topics and sexual history taking exist on *MedEdPORTAL,* none exist specifically related to FSD.^23,24^

Therefore, we developed a didactic session and a facilitated workshop that would provide primary health care professionals (e.g., trainees, physicians, nurses) with a comprehensive approach to diagnosis, screening, evaluation, and treatment of FSD. The workshop delivered relevant and practical content and was designed to encourage active learning via utilization of interactive elements (i.e., debrief of an observed patient-physician discussion, language drills, and case application of knowledge). The overall goal of the sessions was to increase health care practitioners’ knowledge about FSD and comfort initiating conversations about FSD in primary care.

## Methods

### Implementation

We developed this workshop as a tool kit for clinicians and other health professionals seeking to evaluate, treat, and counsel patients about FSD. While targeted to general internists, the content is easily translatable to clinicians in family medicine, medicine/pediatrics, and obstetrics and gynecology who could discuss FSD with their patients.

We developed this session in response to locally identified needs at the Penn State Health Milton S. Hershey Medical Center (PSHMC) and the University of Pittsburgh Medical Center (UPMC) for more knowledge and skill development in FSD. While both institutions were academic medical centers with research and education focused on women's health, faculty had heterogeneous experiences and comfort with evaluation and treatment of FSD. We piloted this session at both PSHMC and UPMC as 60-minute virtual clinical updates for their respective divisions of general internal medicine. The sessions were attended by 10 PSHMC faculty and 28 UPMC faculty in 2020.

We then modified the sessions based on informal feedback from UPMC and PSHMC and adapted them for the Society of General Internal Medicine (SGIM) and the Office of Women's Health for the Veterans Health Administration (VHA). We delivered a 60-minute didactic webinar at VHA in March 2021. Additionally, we were competitively selected to present the material as an interactive 90-minute virtual workshop at SGIM in April 2021. For the subsequent 90-minute workshop, we adapted the 60-minute didactic material to include more audience interaction (case-based small-group breakout sessions, frequent prompts to encourage large-group discussion, and opportunities for communication skills practice).

We include two versions of the session in this publication: the 60-minute didactic (Appendix A) and the 90-minute workshop (Appendix B). Both were designed to encourage audience participation, with increased time for reflection and skills application in the 90-minute workshop. Both started with an overview of the justification for why discussing FSD was important, followed by content on the *DSM-V* categories of FSD^25^ and etiologies of FSD. Next, we presented three clinical cases for each of the three types of FSD most commonly encountered in primary care (orgasmic disorder, genitopelvic pain or penetration disorder, and sexual interest/arousal disorder). The cases first highlighted an approach to evaluating FSD using the 5 A's framework, a published counseling model used to address sexual function with patients through the following steps: ask, advise, assess, assist, and arrange.^26,27^ Subsequent clinical cases highlighted evidence-based treatment options for FSD along with practical considerations of cost, clinical benefit, side effects, and patient preferences.

Necessary equipment included technology with the ability to display a PowerPoint in person or using a virtual platform and space or technology with the capability of dividinge the group into pairs and smaller groups either in person or with virtual breakout rooms. We created a shared virtual folder to distribute handouts (Appendices C and D) for our virtual session.

We outline additional implementation considerations for each session below.

### 60-Minute Didactic (Appendix A)

The recommended timeline was as follows:

• 0–5 minutes: introduction: overview of learning objectives, contextualization and definition of FSD.
• 6–10 minutes: the 5 A's framework and additional workup considerations for patients with FSD.
• 11–40 minutes: treatment of FSD overview and cases.
• 41–50 minutes: sexual devices overview.
• 51–60 minutes: take-home points, debrief, and Q&A.

#### Introduction (Appendix A, slides 1–8)

We began the workshop by identifying stated learning objectives and defining and contextualizing FSD.

#### 5A's framework and additional workup considerations (Appendix A, slides 9–12)

Next, we used a patient case to introduce the 5A's framework. We used prompts to encourage audience participation verbally or by chat (e.g., “Would you consider any additional workup?”).

#### Treatment of FSD overview and cases (Appendix A, slides 13–35)

Using patient cases to prompt discussion with the chat feature, we then reviewed pharmacologic and nonpharmacologic treatments for FSD in a brief didactic. The overview of treatment options in each case was evidence based, with practical considerations of cost, clinical benefit, side effects, and patient preferences.

#### Sexual devices overview (Appendix A, slides 36–43)

The last section of the session provided a brief overview of screening for the safe use of sexual devices.

#### Take-home points, debrief, and Q&A (Appendix A, slides 44–47)

We allotted time for sharing of experiences and open questions, as well as discussing resources for patients with FSD.

In future implementations, if increased time for discussion of cases within the 60-minute session is desired, we recommend removing the content on sexual devices (Appendix A, slides 36–43), which will provide an additional 10 minutes for case-based discussion. The article cited in Appendix A, slides 36–43, could then be given to participants as supplementary material for independent review.

### 90-Minute Workshop (Appendix B)

This workshop followed the same outline as above, with an additional debrief of an observed patient-physician discussion and two small-group discussions. The recommended timeline was as follows:

• 0–5 minutes: introduction and icebreaker/large-group discussion.
• 6–20 minutes: approach to FSD and the 5 A's framework.
• 21–35 minutes: overview of treatments for FSD and cases.
• 36–50 minutes: small-group discussion 1 on treatment of FSD cases.
• 51–65 minutes: large-group debrief of patient cases.
• 66–70 minutes: sexual devices overview.
• 71–75 minutes: small-group discussion 2 on language drills for sexual devices communication.
• 76–90 minutes: debrief, Q&A, and evaluation.

#### Introduction (Appendix B, slides 1–9)

We began the workshop by identifying participants’ barriers to discussing FSD in the clinic via group discussion: “What are the challenges to discussing sexual dysfunction with your female patients?”

#### Approach to FSD (Appendix B, slides 10 and 11, and Appendix E)

After an overview of categories of FSD, the 5A's framework was introduced. Workshop leaders demonstrated a physician-patient discussion about sexual function using a script (Appendix E). Next, we encouraged reflection on the observed discussion through prompts (e.g., “What did you notice?”). Finally, we instructed the group to work together for 2–3 minutes to identify which aspects of the 5A's framework had been seen in the observed patient-physician exchange.

#### Treatment of FSD overview and cases (Appendix B, slides 12–27, and Appendix C)

We then reviewed pharmacologic and nonpharmacologic treatments for FSD in a brief didactic. We divided participants into virtual breakout rooms (four to five participants in each room) to review and discuss effective treatment plans for one of three cases (Appendix C). Next, we reconvened as a large group to debrief these three case scenarios one at a time. We invited a representative from each small group to summarize the discussion from their small group, then opened discussion up to the larger group.

#### Sexual devices overview and language drills (Appendix B, slides 28–38, and Appendix D)

The last section of the workshop provided a brief overview of screening for the safe use of sexual devices. We divided participants into pairs and asked dyads to utilize language drills^28^ to practice sensitive language for screening/counseling regarding sexual devices (Appendix D). The language drills included two different scripts of a patient and physician discussing sexual devices. For the first case, one person in each pair was the physician and the other the patient. The pair then read through the script together. For the second case, the participants switched roles and read through the second script. After reading through both scripts, we prompted the group as a whole to reflect on the responses to the language used.

#### Wrap-up (Appendix B, slides 39–41, and Appendix F)

We allotted time for sharing of experiences and open questions, as well as discussing resources for patients with FSD. At this time, we also distributed the session evaluation (Appendix F).

For future implementations, we recommend that session learners and facilitators have prerequisite knowledge about obtaining a sexual history in women as well as experience in communication about sexual concerns. We also recommend that facilitators gain familiarity with local resources for patients with FSD in their area or medical system (e.g., relevant medications on formulary, referrals for specialists and pelvic floor physical therapy) to assist with discussion during the session.

### Evaluation

We evaluated the workshop at SGIM using a survey (Appendix F) administered to all participants immediately after the workshop. Two study authors (Sarah Merriam and Rachel S. Casas) developed the survey, which was adapted from a prior *MedEdPORTAL* publication.^29^ The survey was piloted by two authors (Juliana M. Kling and Holly N. Thomas) and refined based on feedback received. All surveys were anonymous, completion was voluntary, and participants were not required to answer all questions. The survey began with several demographic questions assessing participants’ gender, academic rank, and practice setting. We incorporated questions about baseline comfort and experience with FSD and about current practice patterns, as well as an assessment of overall rating of workshop quality, including open-ended questions about impact on practice and recommendations to improve the workshop in future iterations.

Because we presented as a part of the national Veterans Affairs Women's Health webinar series, that evaluation was dictated by the VHA Employee Education System. Voluntary surveys were disseminated electronically and included occupational category, perceived overall satisfaction and overall content, nine standard evaluation questions, and four program-specific evaluations tied to workshop objectives. All questions used a 5-point Likert scale (1 = *strongly disagree,* 5 = *strongly agree*). Learner assessment of objectives occurred informally during small-group discussions and debriefs for the cases where participants were asked to identify contributing factors to FSD, formulate diagnostic plans, and discuss treatment options.

## Results

### Setting and Participants

Twenty-seven general internists attended the SGIM workshop, and 220 women's health physicians, physician assistants, nurses, pharmacists, psychologists, and social workers attended the VHA didactic.

### Participation and Response Rates

We collected four evaluations from the SGIM workshop (response rate: 15%). All respondents (*n* = 4) identified as female, were early in their career (one medical student, one resident, one clinical instructor, and one assistant professor), and infrequently discussed sexual dysfunction with female or LGBTQI patients (100% <6x/year). Respondents represented regions across the United States (50% Mid-Atlantic, 25% California/Hawaii, 25% Midwest). Open-ended responses about workshop quality (*n* = 2) included “Excellent” and “Great job of addressing an important topic.”

We collected 131 evaluations from the national VHA didactic (response rate: 60%). Attendees represented a variety of occupational categories (40% physician, 27% nurse, 8% social worker, 3% psychologist, 2% pharmacist, 2% physician assistants, 1% physical therapist, 18% not identified).

### Satisfaction With Curriculum

In both the SGIM and VHA sessions, respondents (*n* = 135) across a spectrum of health occupations and prior experience rated the sessions highly with regard to content (*M* = 4.1) and overall evaluation of the session (*M* = 4.3). VHA didactic participants were highly satisfied with the webinar, found that it advanced interprofessional care and collaborative practice, and felt it was an effective training environment (Table 1). Similarly, they rated accomplishment of session-specific objectives highly (Table 2).

**Table 1.**
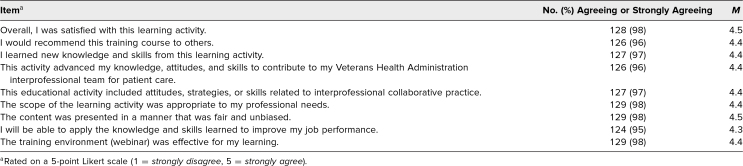
Reported Agreement With Workshop Evaluation (*N* = 131)

**Table 2.**
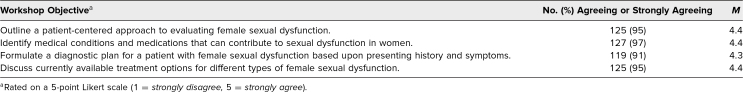
Reported Agreement With Workshop-Specific Learning Objectives (*N* = 131)

In the SGIM workshop, we prompted participants to identify one change in clinical practice that they would pursue based upon the session; all respondents (*n* = 4) identified an action related to initiating conversations about sexual function and device use. Suggestions for improvement included providing a summary handout with specific treatment recommendations (e.g., pharmacologic brands with dosing guidance, nonpharmacologic treatments) in future iterations.

## Discussion

Female sexual function is linked to overall well-being and relationship satisfaction but is underrecognized and undertreated. Evaluation and treatment of FSD in primary care is limited in part due to lack of training and knowledge in this area. This workshop supports primary health care professionals by improving their ability to diagnose and treat FSD and their skills in patient-centered, effective communication.

We developed these sessions based upon identified knowledge and skill deficits at our institutions but implemented the session on national platforms, as there is a widespread gap in FSD training for primary health care professionals. One of our key reflections during the development of the sessions was that there remains a dearth of pharmacologic options for FSD that are widely available and covered by insurance.^30^ This was one of the key challenges identified by participants during the sessions and remains an area where more viable options are needed. We note that the pharmacology treatment section of these resources can evolve and hope that the section will require frequent updating as new treatment options arrive.

While we implemented this session across multiple local and national audiences, we saw some common responses during and after implementation. As this can be an uncomfortable topic to discuss, it sometimes took a while for participants to actively join in the conversation. For this reason, we encouraged participation, particularly at the beginning of the session, through anonymous polling and through the chat function of our virtual platform. Sessions conducted in a face-to-face setting could utilize think-pair-share to facilitate interactive discussion in lieu of chat functions. We used a debrief of an observed patient-physician discussion and language drills to give participants the chance to reflect on and practice using language to discuss FSD without having to come up with what to say on their own. Our goal with these sessions was to normalize discussing FSD with colleagues and patients, so that everyone would be comfortable having these conversations. We found that participants were thankful to have an opportunity to discuss a sensitive topic and to feel equipped with language to discuss FSD with patients. For sessions with high group safety and/or higher baseline participant skill with respect to this content area, facilitators could increase active participation by asking learners to demonstrate communication skills themselves through role-play. Notably, the format for both session lengths is adaptable to smaller groups if participants are engaged in discussion or to larger groups if breakout sessions for cases are used.

There were a number of limitations to this project. We provided adaptable resources that could be used in multiple educational settings (virtual and in person) and available curricular space. The sessions were successfully implemented among colleagues who know each other well and those meeting for the first time.

With respect to implementation, we recognize that the exercises included in these sessions may not be at the correct level for all learners and that full skill building in FSD counseling may require practice outside of these sessions. Most participants in each session identified as women's health clinicians, but a more general audience may require broader training with respect to sexual history taking in order to implement the communication skills related to FSD.^31^ Participants with less baseline knowledge and comfort regarding sexual health and communication could consider reviewing available resources on this topic, such as the provider's guide from the National Coalition for Sexual Health.^32^ Though language drills have been demonstrated to improve participant comfort with sensitive language,^28,33^ we have no direct evidence that this occurred in our sessions. We also recognize that providing a safe space for learners to practice new language and skills is essential to higher-level learning and change in practice.

Our evaluation showed high satisfaction and intention to change practice related to treatment/evaluation of FSD among participants following the session. Our evaluation, however, was limited to self-report, and we did not directly assess learners’ knowledge and skills outlined in the learning objectives. Additionally, response rate was low for the 90-minute workshop, and therefore, any conclusions drawn from this survey are limited and may not accurately reflect the perceptions of the majority of participants. It is unknown if our intervention led to any changes in behavior for our participants or in clinical outcomes in patient care. The evaluation was cross-sectional and did not allow for comparisons of learners pre-/postintervention. We also acknowledge that evaluation of the VHA session was limited by the requirement to use a standardized assessment tool.

Another limitation to generalizability of these materials could be for primary health care professionals working in areas with limited resources for patients with FSD (e.g., lack of access to pelvic floor physical therapy) or for clinicians working outside the United States, where available treatment options may differ. Finally, our sessions apply predominately to cisgender women.

Future iterations of these sessions on FSD could include dedicated skill practice and direct assessment of the skills. While we used observed patient-physician discussions and language drills to provide examples of discussing FSD with patients, offering dedicated skill practice (e.g., role-play or work with standardized patients) with feedback might be more effective in enhancing communication skills in this domain. Future assessments could ask participants to reflect directly on the impact of language drills on their comfort with discussing FSD, which could then be analyzed qualitatively. The learning objectives could be directly assessed by asking participants to write down their own responses to questions posed in the presentation's clinical vignettes, which map directly to our learning objectives. Alternatively, using one or more of the clinical vignettes included in Appendix A or B, participants could be asked after the session to write open-ended responses to the following questions:

• Please describe how you would approach evaluating FSD.
• What factors may contribute to FSD in this patient?
• Given the history and symptoms presented in this case, what additional diagnostic workup would you consider for this patient?
• What pharmacologic and nonpharmacologic treatment options might benefit this patient?

Higher levels of assessment could include pre/post knowledge questions addressing conditions, diagnosis, and treatment of FSD and/or attitudinal questions about self-reported comfort with communication around FSD. Communication skills could be further assessed through objective structured clinical exercise evaluation. Finally, application of skills and change in clinical practice could be elucidated through follow-up surveys or chart review of participants.

Evaluation of these sessions could also be improved through changes in survey distribution. Because the survey instrument for the 90-minute workshop was brief, we suspect that the low response rate was related to the timing of survey distribution (at the end of the session) and to the virtual environment. If conducting this training virtually in future sessions, we would recommend making a statement at the outset of the training indicating that a link to a survey will be distributed, and then releasing the link in advance of the question-and-answer period at the end of the session. If delivering a live session, we recommend distributing the survey in paper format prior to the end of the session and reserving time for survey completion to encourage participation. If future trainings are bound to a standard evaluation form as at the VHA, we recommend also disseminating the survey in Appendix F that is specific to the session. One limitation of this approach might be a less robust response rate, given multiple surveys.

In the future, it would be valuable to implement these sessions with an interdepartmental audience (e.g., obstetrics and gynecology, family medicine, internal medicine) to provide a platform for discussion of approaches to FSD by each of these specialties. We have adapted (but not yet studied) a simplified version of this session for medical students, with a focus on physiology and pathophysiology. We hope that sessions like these can become part of a larger standardized women's health curriculum for primary care trainees and practicing health care professionals, particularly in areas or institutions where local experts are lacking. Future iterations of this content could broaden scope to include diagnosis and treatment of FSD in transgender women.

Overall, these results demonstrate that a diverse audience of health care professionals reported high satisfaction and increased knowledge of FSD following the workshops. These easily generalizable materials can be adapted to a variety of educational settings and may help close existing education gaps for health care professionals caring for women.

## Appendices


60-Minute Didactic.pptx90-Minute Workshop.pptxDiscussion Cases.docxSexual Devices Language Drills.docxRole-Play Script.docxEvaluation.docx
*All appendices are peer reviewed as integral parts of the Original Publication.*
