# Supplementary material for: Female Sexual Dysfunction: A Primer for Primary Care Health Professionals
Source: MedEdPORTAL. 2023 Apr 25;19:11312. doi: 10.15766/mep_2374-8265.11312 (PMC10126124; doi:10.15766/mep_2374-8265.11312)
Supplement: Supplementary file 1 — 60-Minute Didactic.pptx90-Minute Workshop.pptxDiscussion Cases.docxSexual Devices Language Drills.docxRole-Play Script.docxEvaluation.docx [file mep_2374-8265.11312-s001.zip › A. 60-Minute Didactic.pptx]

## Slide 1
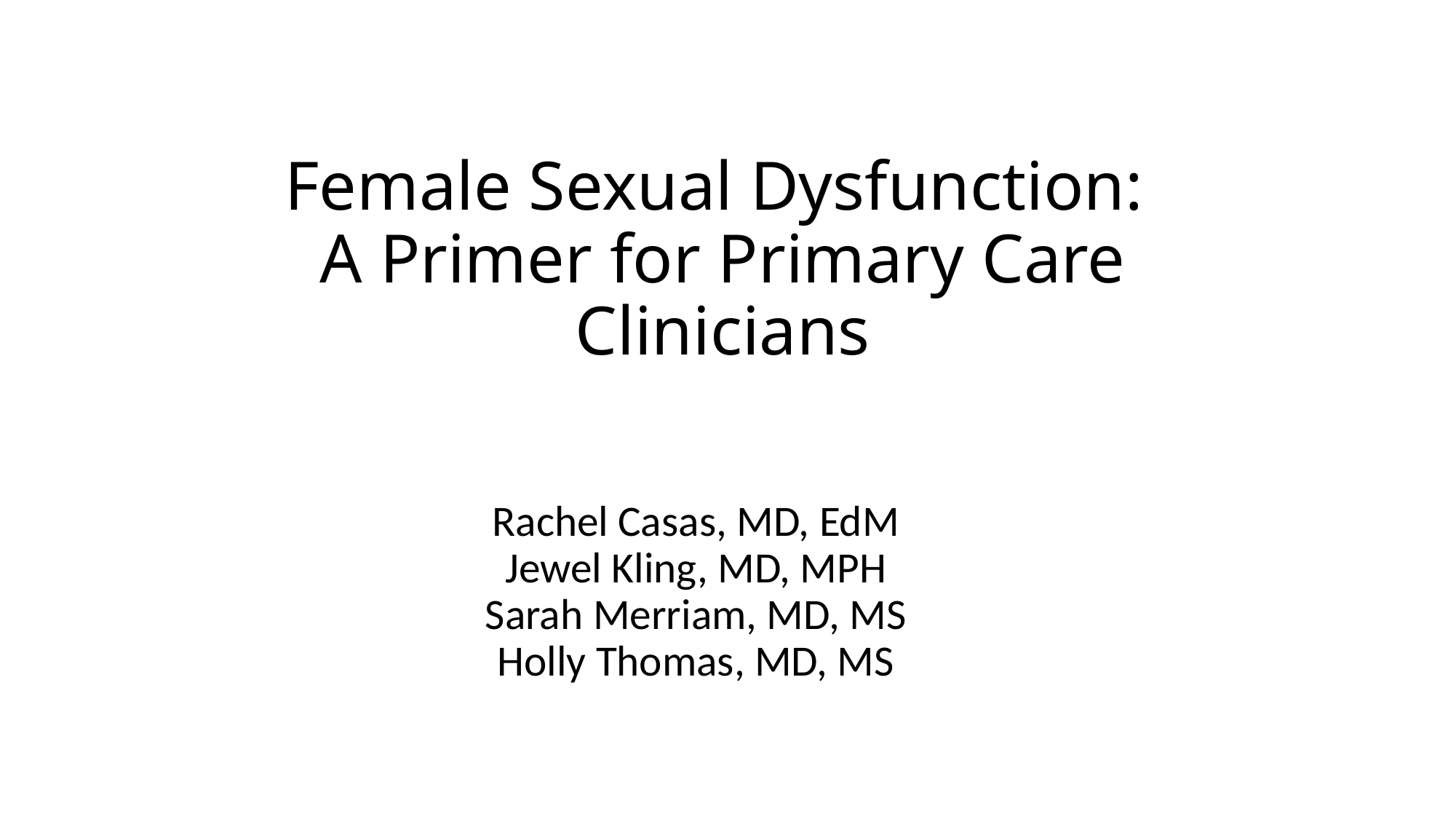

Female Sexual Dysfunction:
A Primer for Primary Care Clinicians
Rachel Casas, MD, EdM
Jewel Kling, MD, MPH
Sarah Merriam, MD, MS
Holly Thomas, MD, MS

## Slide 2
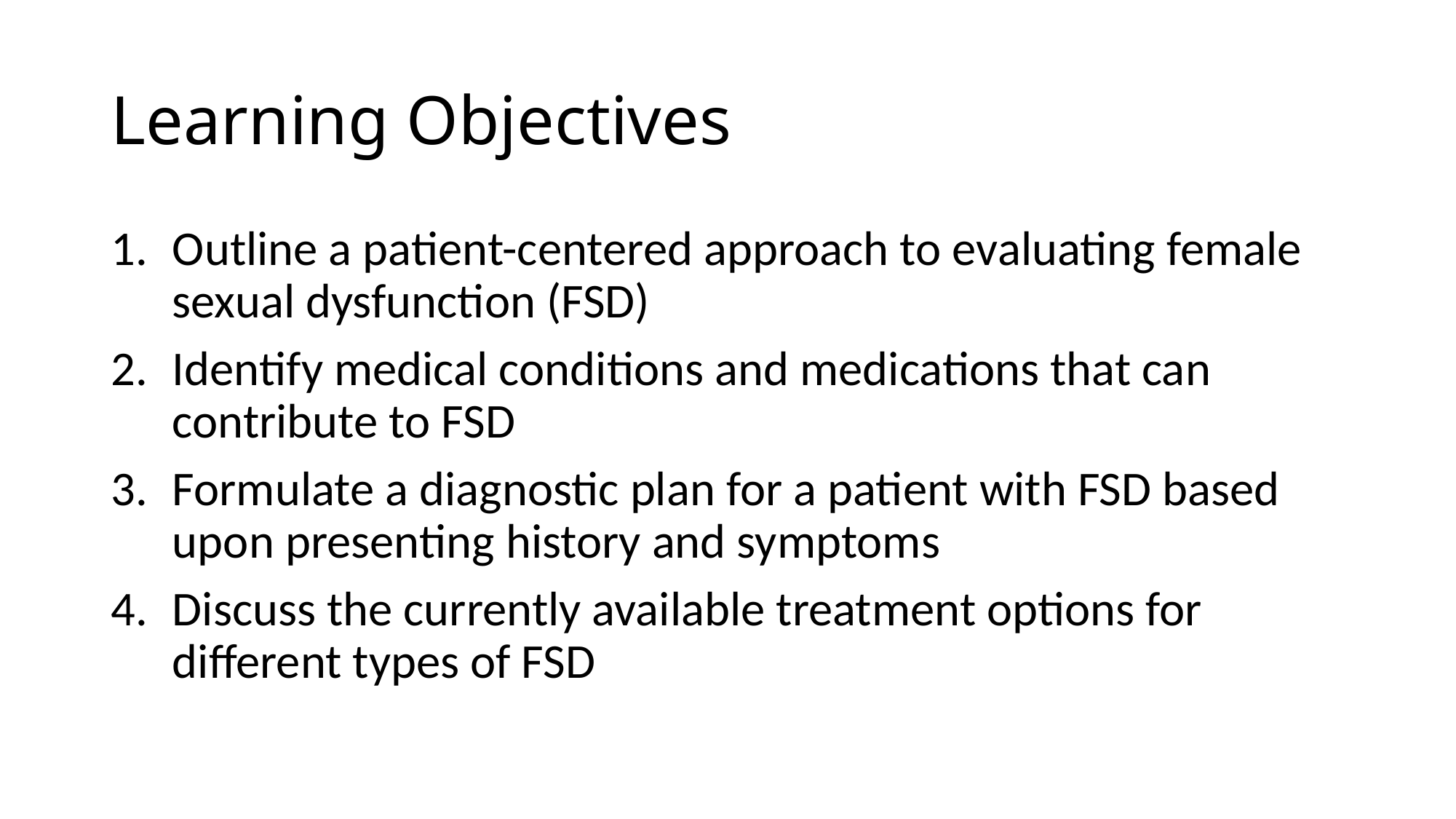

# Learning Objectives
Outline a patient-centered approach to evaluating female sexual dysfunction (FSD)
Identify medical conditions and medications that can contribute to FSD
Formulate a diagnostic plan for a patient with FSD based upon presenting history and symptoms
Discuss the currently available treatment options for different types of FSD

## Slide 3
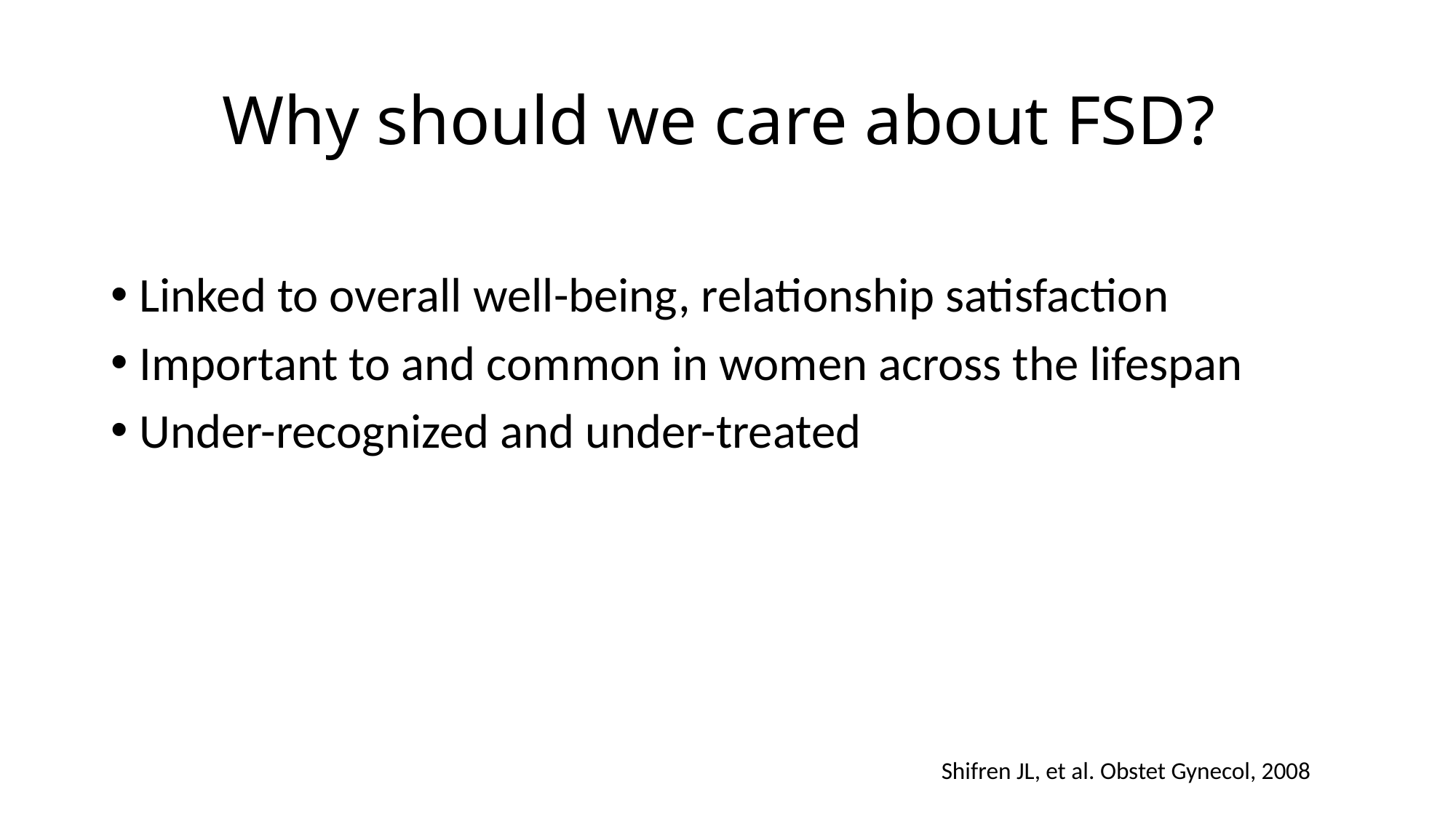

# Why should we care about FSD?
Linked to overall well-being, relationship satisfaction
Important to and common in women across the lifespan
Under-recognized and under-treated
Shifren JL, et al. Obstet Gynecol, 2008

## Slide 4
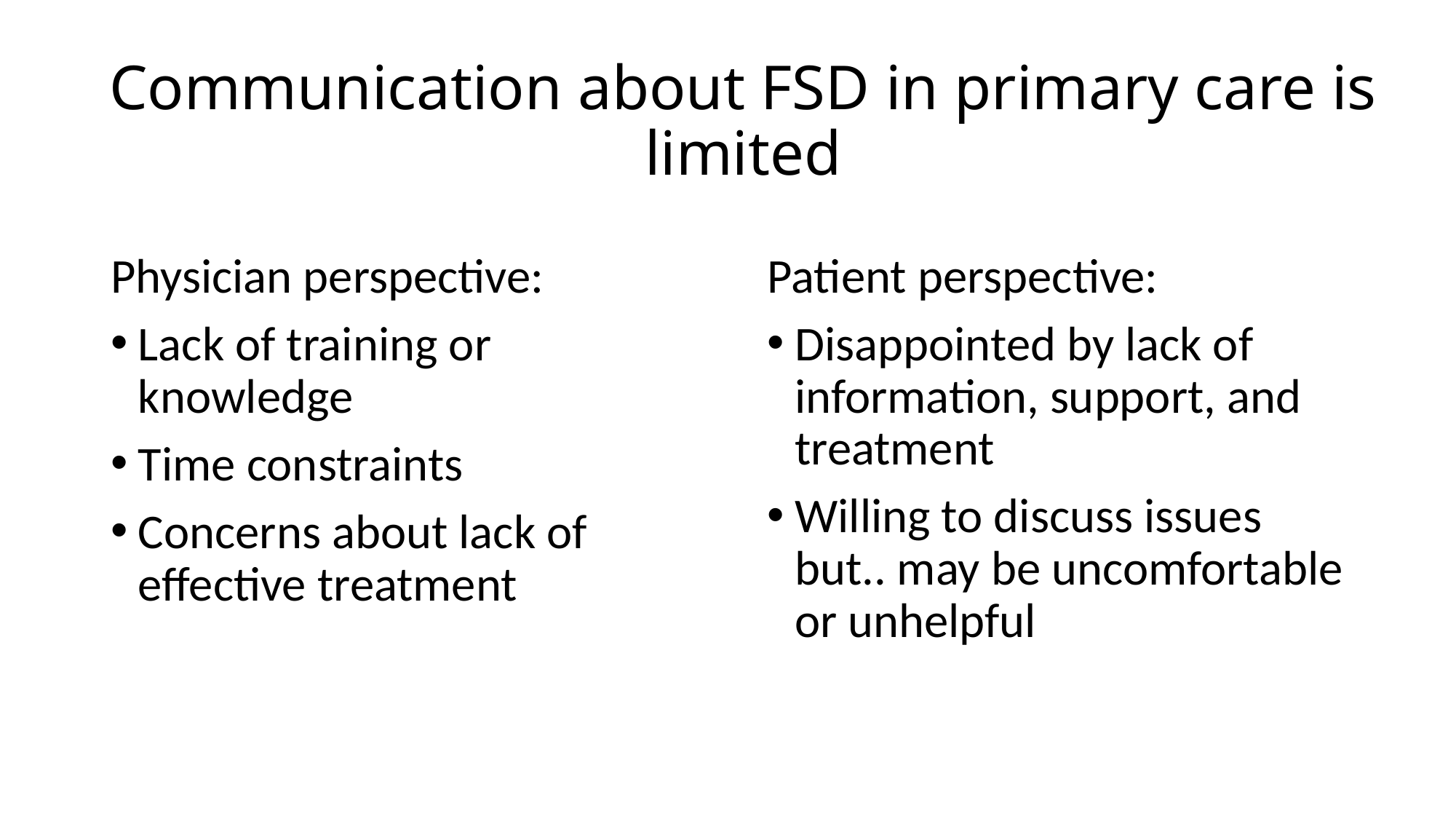

# Communication about FSD in primary care is limited
Physician perspective:
Lack of training or knowledge
Time constraints
Concerns about lack of effective treatment
Patient perspective:
Disappointed by lack of information, support, and treatment
Willing to discuss issues but.. may be uncomfortable or unhelpful

## Slide 5
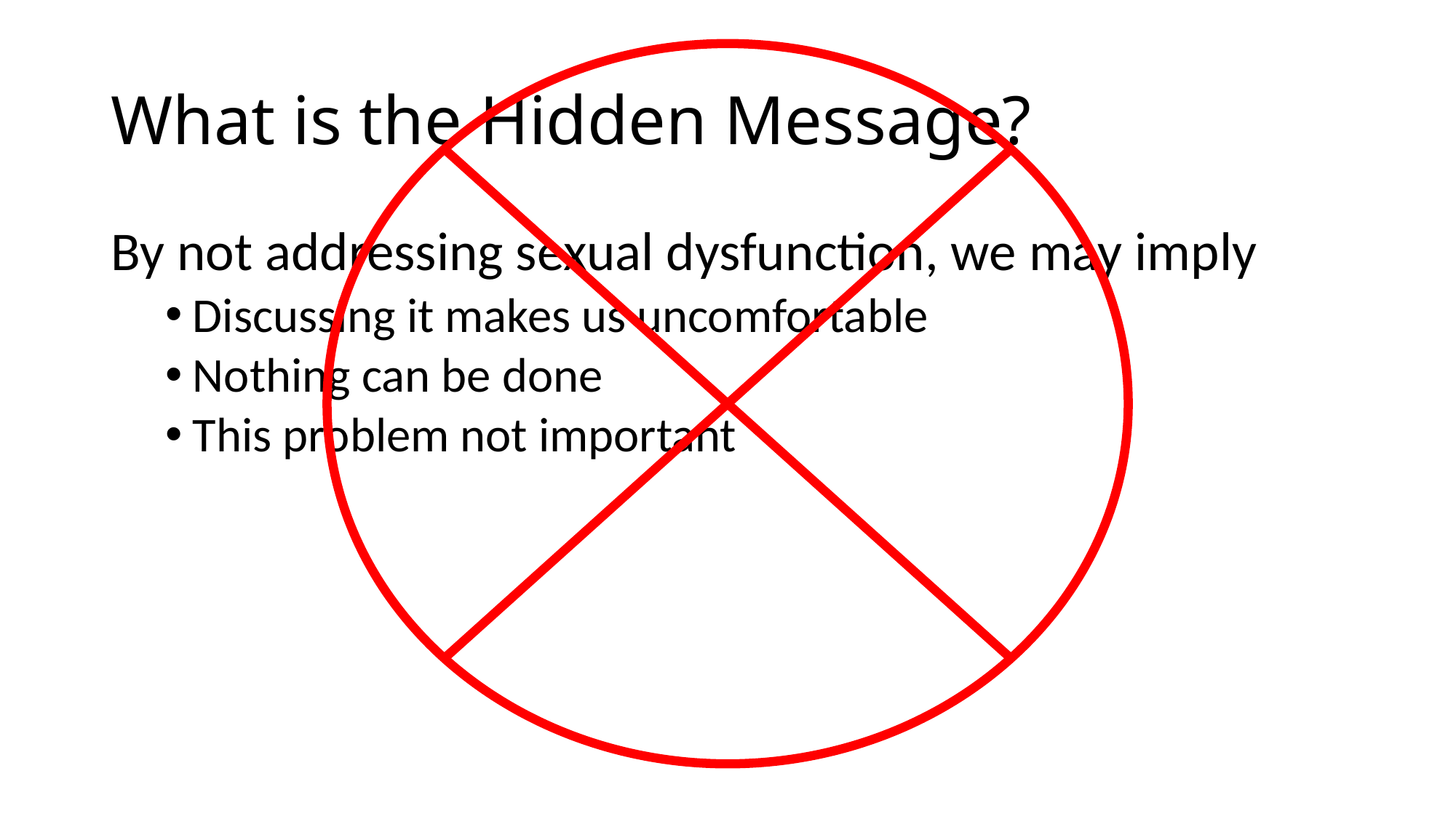

# What is the Hidden Message?
By not addressing sexual dysfunction, we may imply
Discussing it makes us uncomfortable
Nothing can be done
This problem not important

## Slide 6
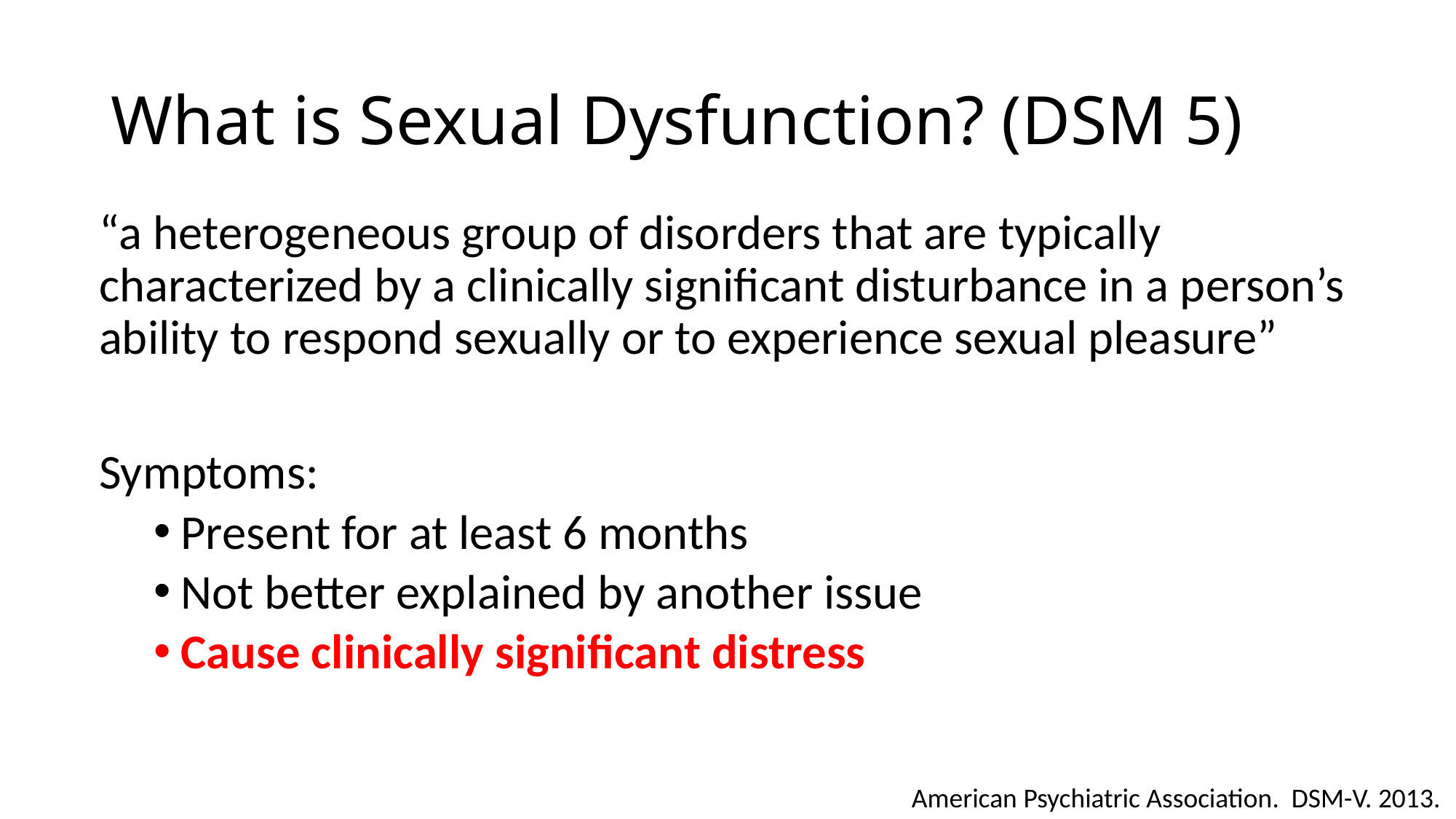

# What is Sexual Dysfunction? (DSM 5)
“a heterogeneous group of disorders that are typically characterized by a clinically significant disturbance in a person’s ability to respond sexually or to experience sexual pleasure”
Symptoms:
Present for at least 6 months
Not better explained by another issue
Cause clinically significant distress
American Psychiatric Association. DSM-V. 2013.

## Slide 7
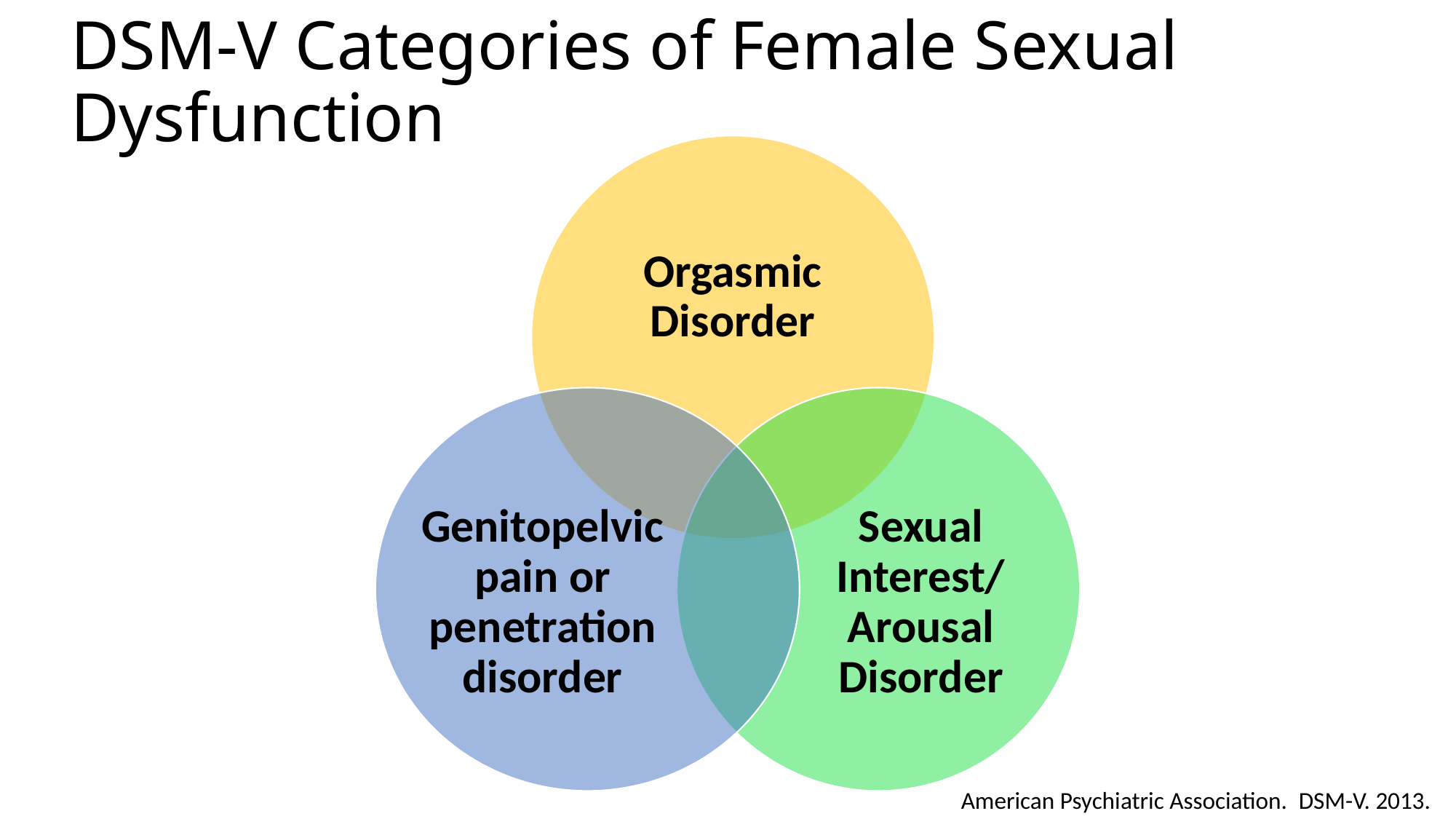

# DSM-V Categories of Female Sexual Dysfunction
Orgasmic Disorder
Genitopelvic pain or penetration disorder
Sexual Interest/ Arousal Disorder
American Psychiatric Association.  DSM-V. 2013.

## Slide 8
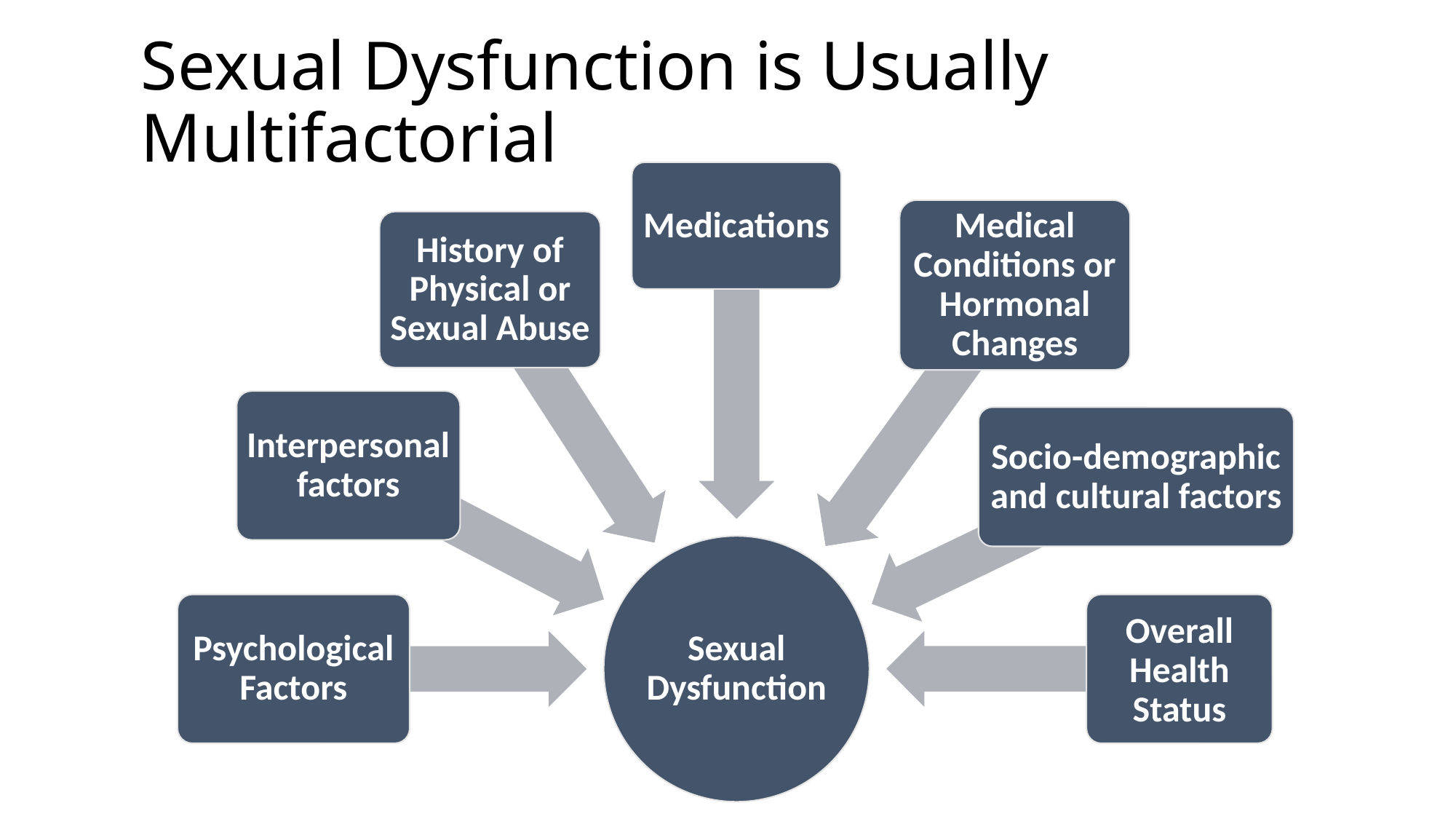

# Sexual Dysfunction is Usually Multifactorial
Medications
Medical Conditions or Hormonal Changes
History of Physical or Sexual Abuse
Interpersonal factors
Socio-demographic and cultural factors
Sexual Dysfunction
Psychological Factors
Overall Health Status

## Slide 9
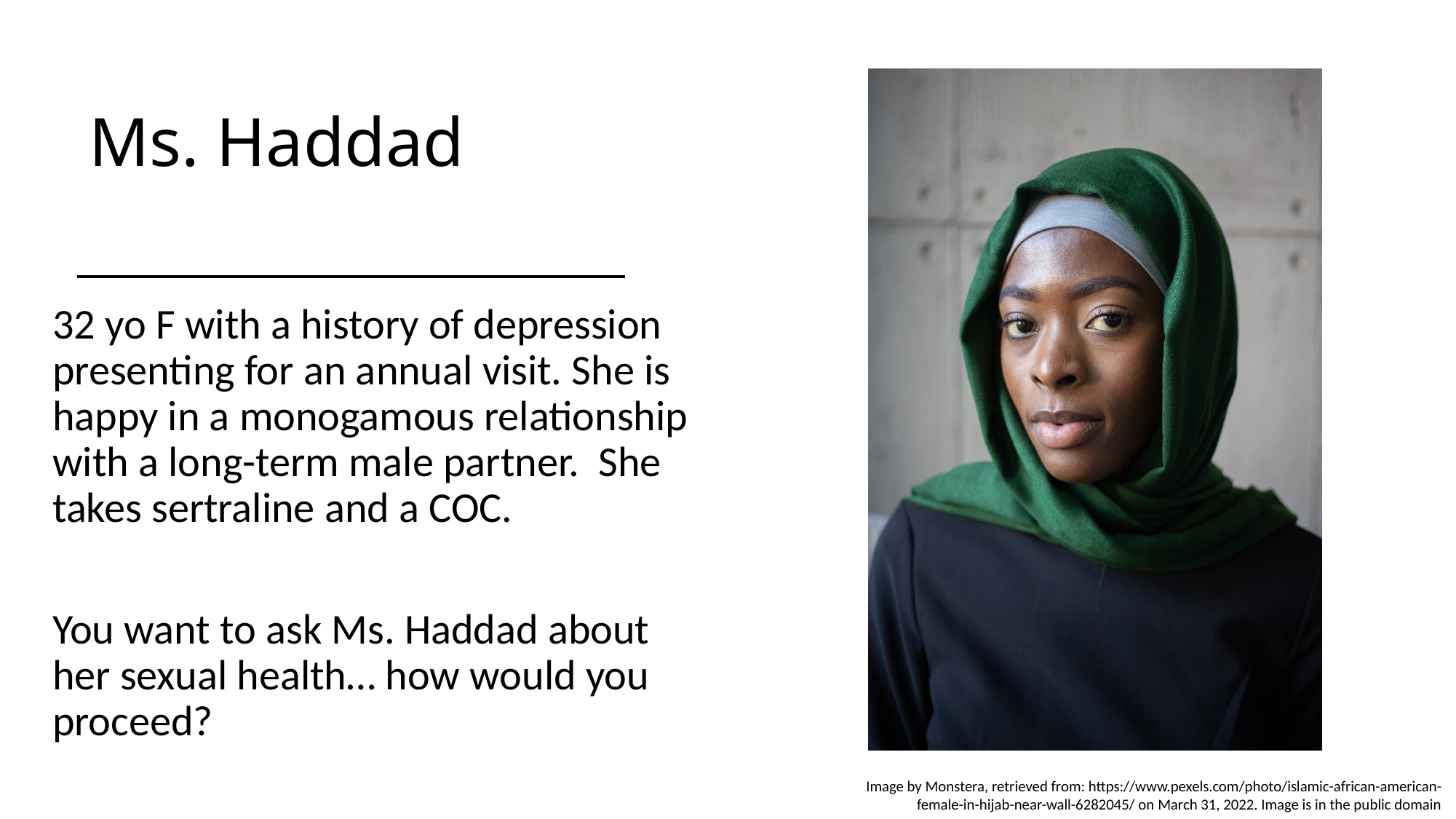

# Ms. Haddad
32 yo F with a history of depression presenting for an annual visit. She is happy in a monogamous relationship with a long-term male partner. She takes sertraline and a COC.
You want to ask Ms. Haddad about her sexual health… how would you proceed?
Image by Monstera, retrieved from: https://www.pexels.com/photo/islamic-african-american-female-in-hijab-near-wall-6282045/ on March 31, 2022. Image is in the public domain

## Slide 10
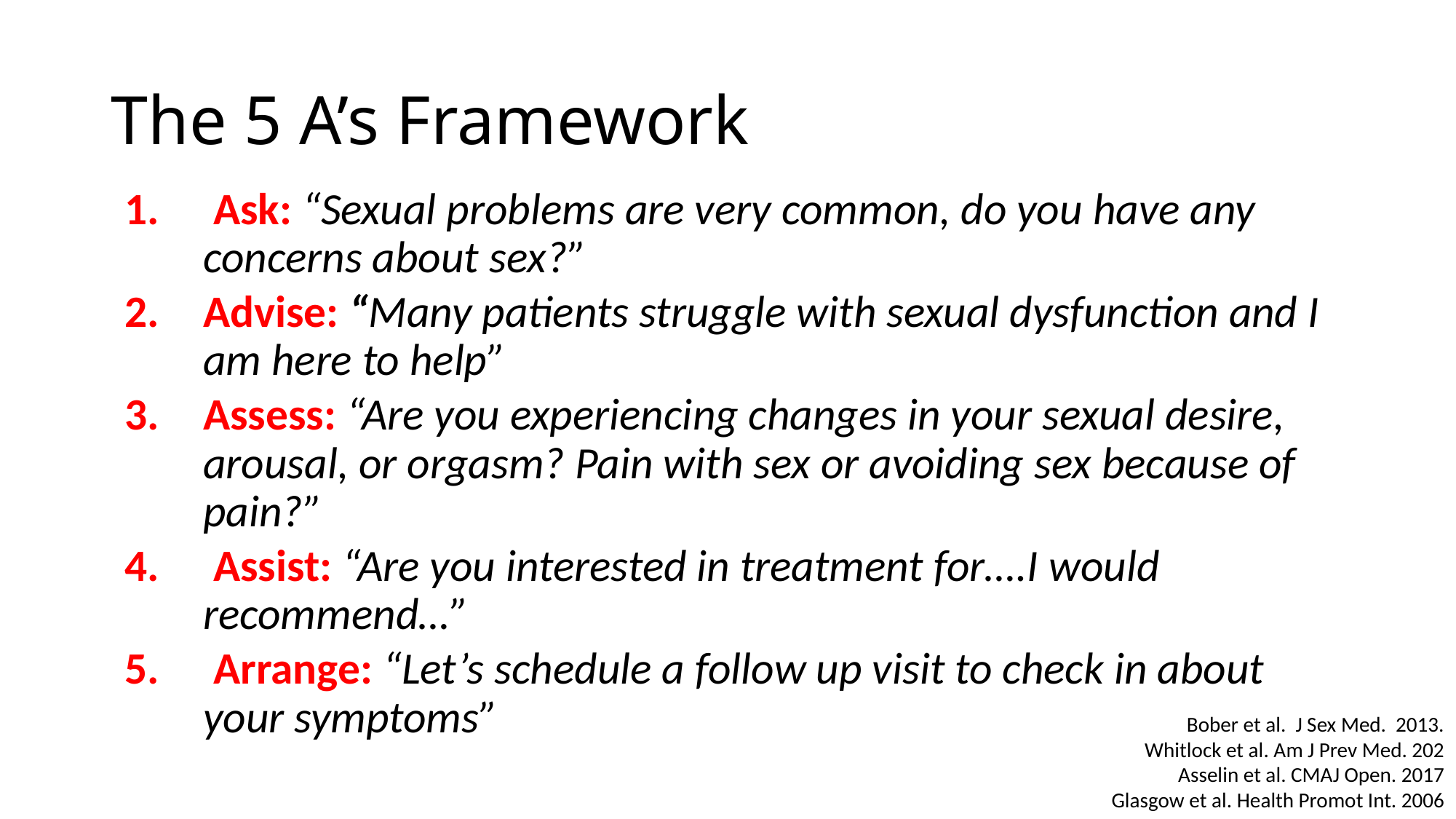

# The 5 A’s Framework
 Ask: “Sexual problems are very common, do you have any concerns about sex?”
Advise: “Many patients struggle with sexual dysfunction and I am here to help”
Assess: “Are you experiencing changes in your sexual desire, arousal, or orgasm? Pain with sex or avoiding sex because of pain?”
 Assist: “Are you interested in treatment for….I would recommend…”
 Arrange: “Let’s schedule a follow up visit to check in about your symptoms”
Bober et al. J Sex Med. 2013.
Whitlock et al. Am J Prev Med. 202
Asselin et al. CMAJ Open. 2017
Glasgow et al. Health Promot Int. 2006

## Slide 11
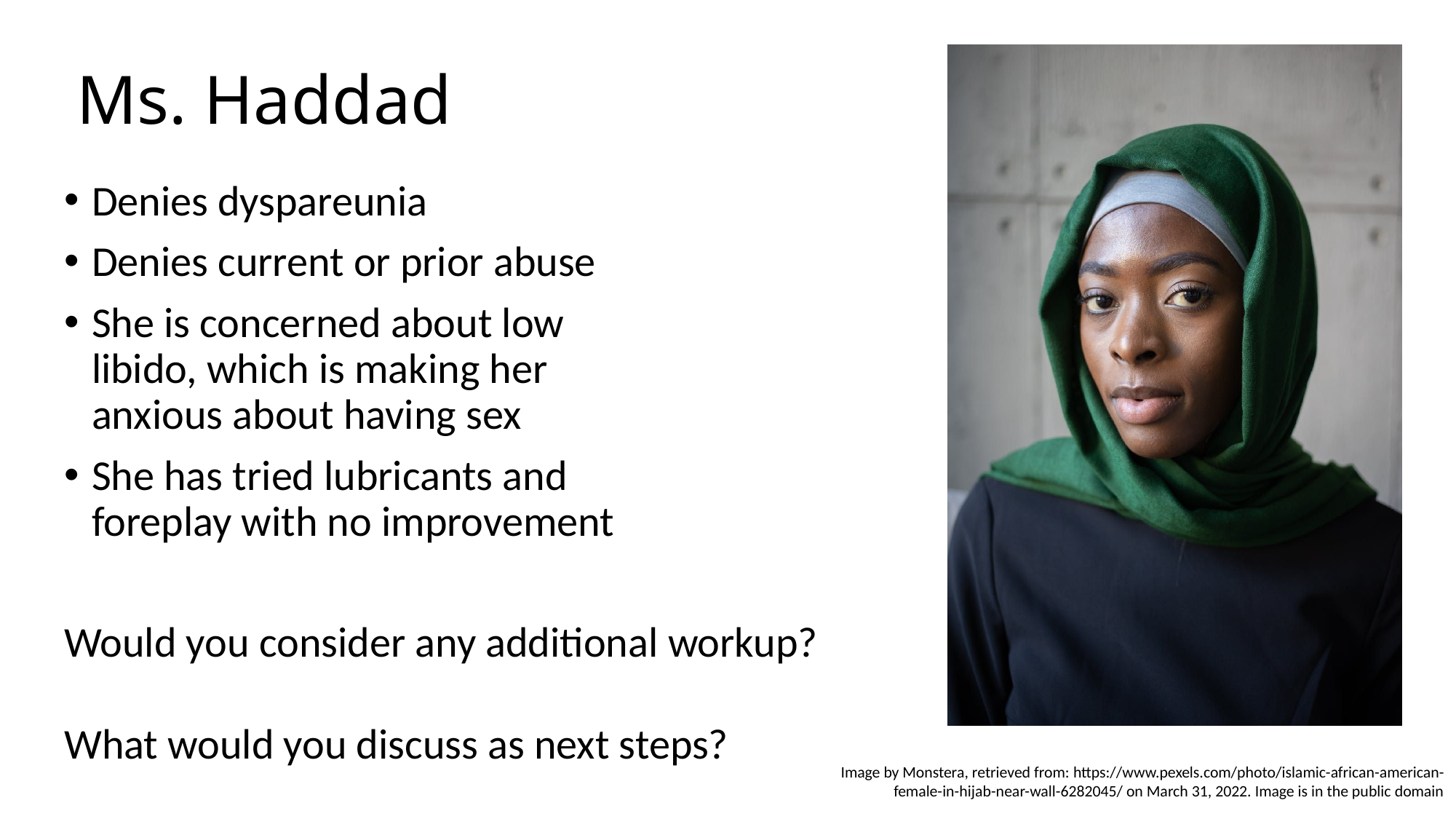

# Ms. Haddad
Denies dyspareunia
Denies current or prior abuse
She is concerned about low libido, which is making her anxious about having sex
She has tried lubricants and foreplay with no improvement
Would you consider any additional workup?
What would you discuss as next steps?
Image by Monstera, retrieved from: https://www.pexels.com/photo/islamic-african-american-female-in-hijab-near-wall-6282045/ on March 31, 2022. Image is in the public domain

## Slide 12
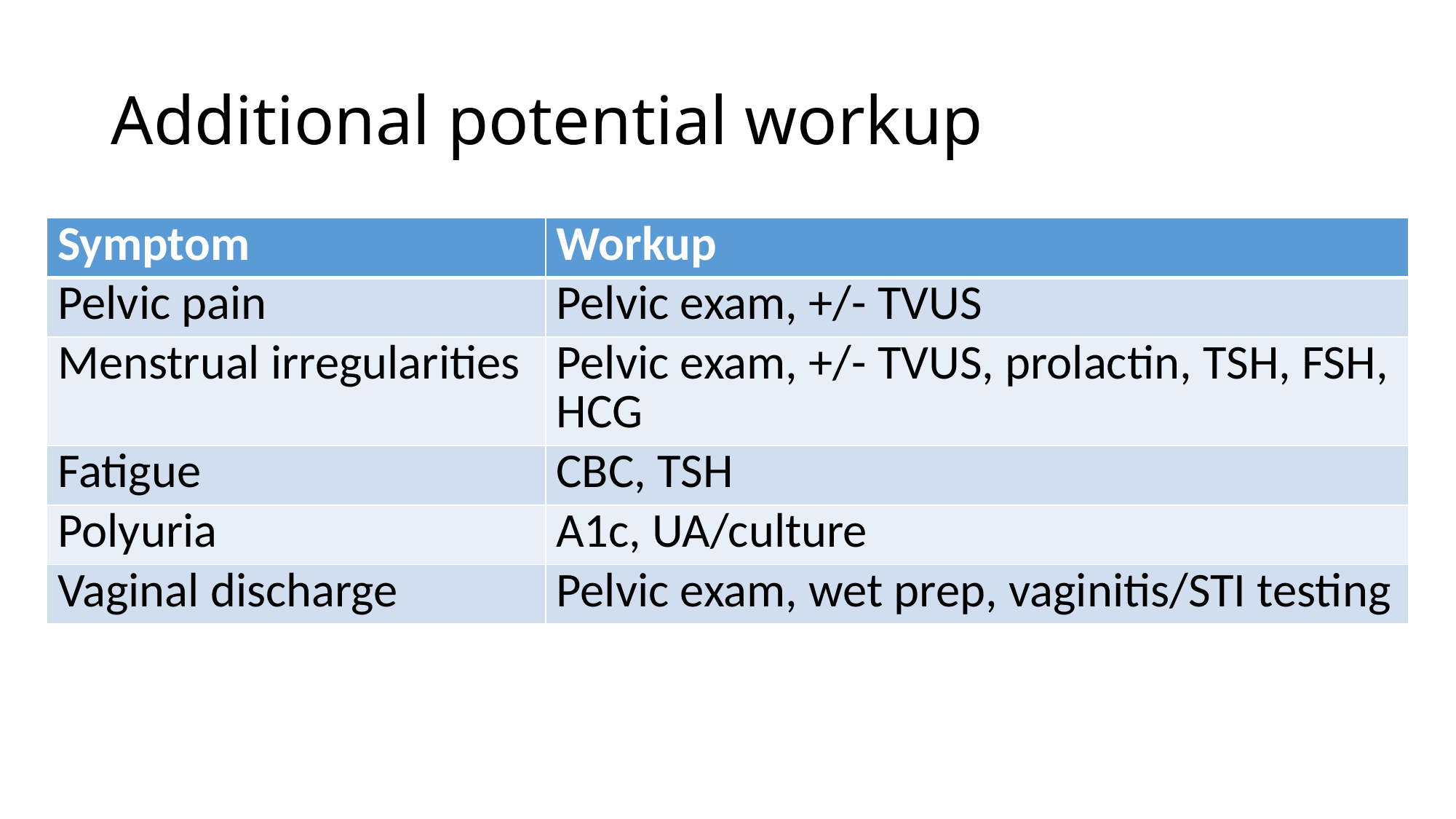

# Additional potential workup
| Symptom | Workup |
| --- | --- |
| Pelvic pain | Pelvic exam, +/- TVUS |
| Menstrual irregularities | Pelvic exam, +/- TVUS, prolactin, TSH, FSH, HCG |
| Fatigue | CBC, TSH |
| Polyuria | A1c, UA/culture |
| Vaginal discharge | Pelvic exam, wet prep, vaginitis/STI testing |

## Slide 13
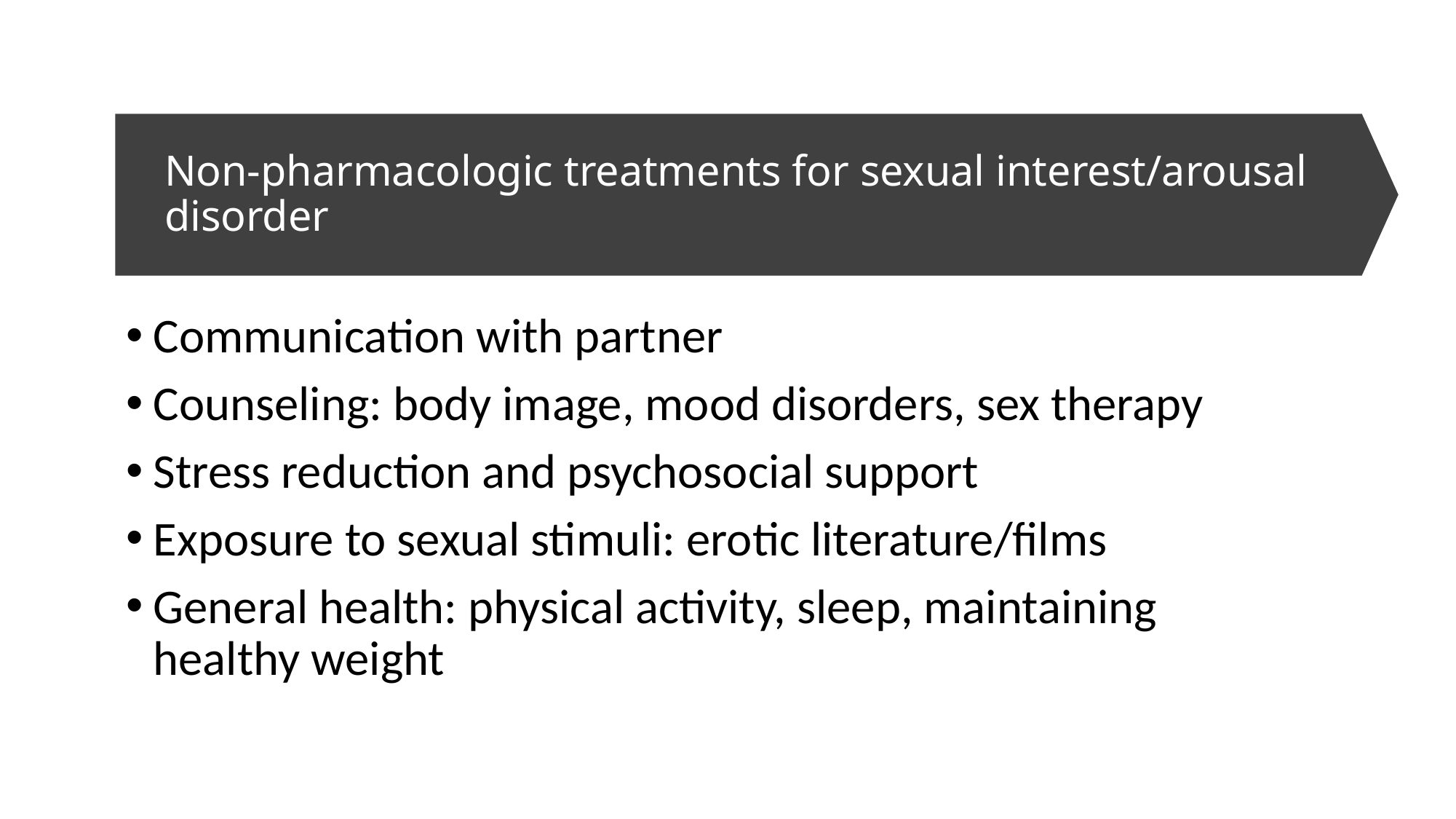

# Non-pharmacologic treatments for sexual interest/arousal disorder
Communication with partner
Counseling: body image, mood disorders, sex therapy
Stress reduction and psychosocial support
Exposure to sexual stimuli: erotic literature/films
General health: physical activity, sleep, maintaining healthy weight

## Slide 14
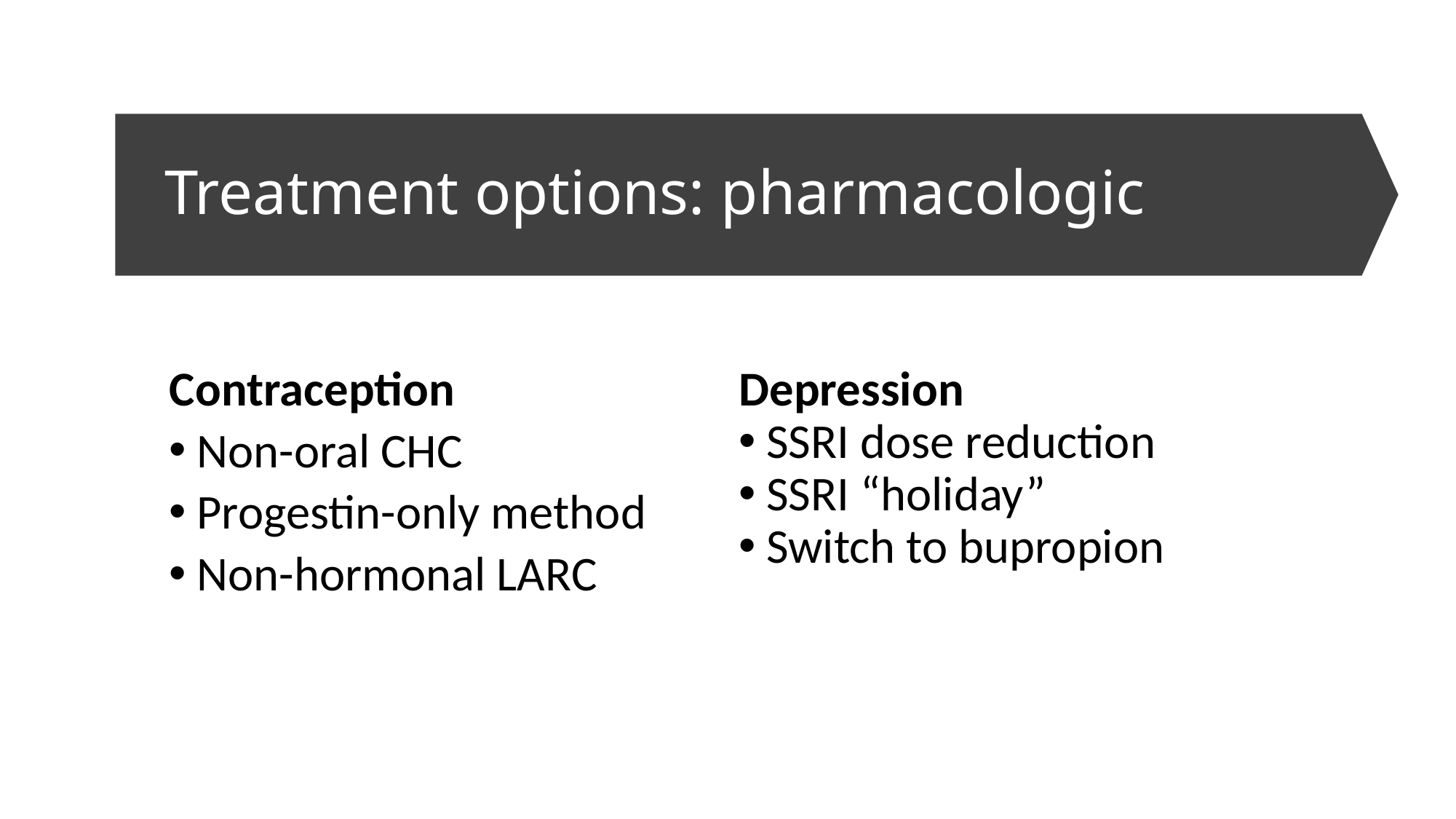

# Treatment options: pharmacologic
Contraception
Non-oral CHC
Progestin-only method
Non-hormonal LARC
Depression
SSRI dose reduction
SSRI “holiday”
Switch to bupropion

## Slide 15
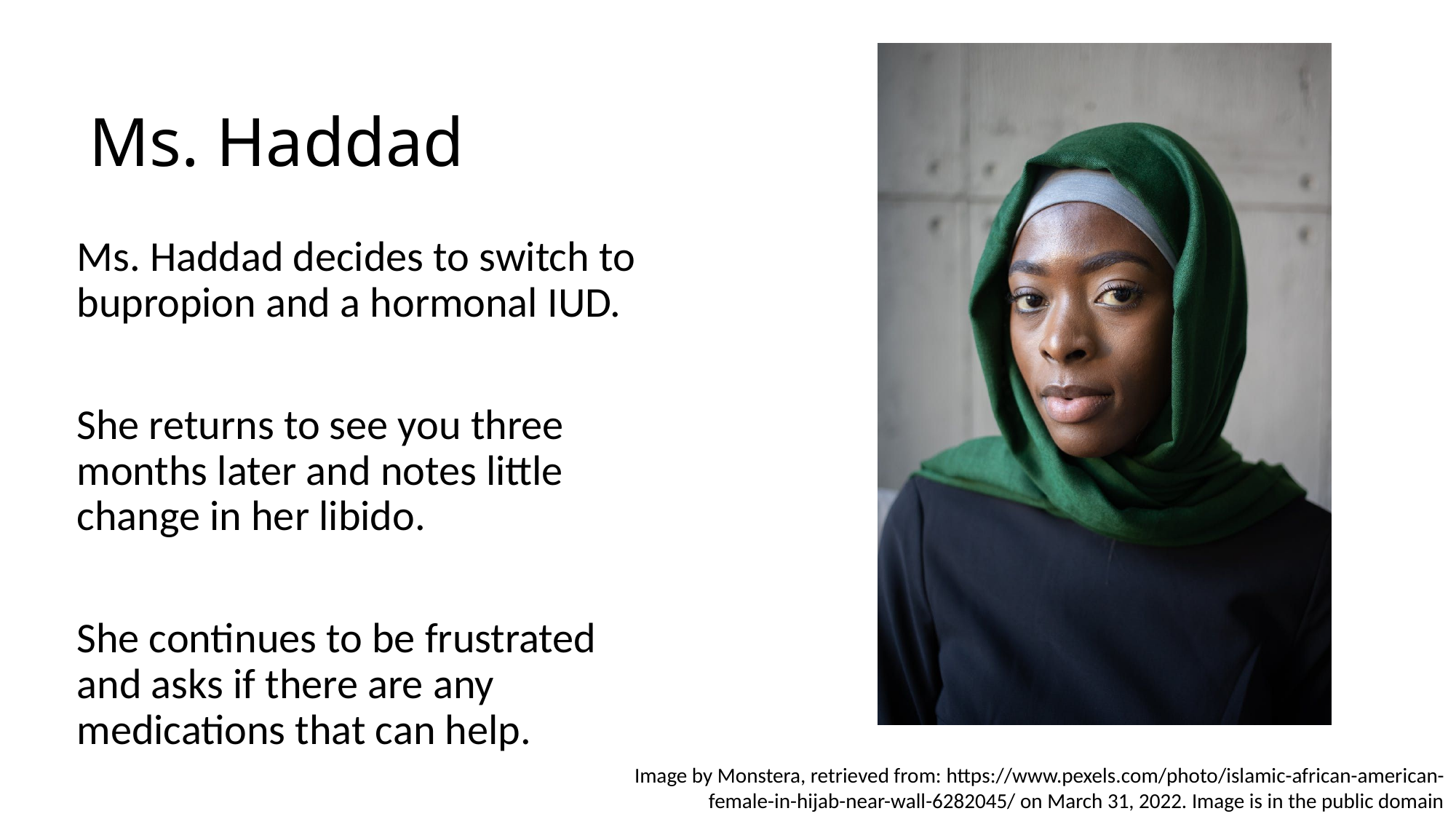

# Ms. Haddad
Ms. Haddad decides to switch to bupropion and a hormonal IUD.
She returns to see you three months later and notes little change in her libido.
She continues to be frustrated and asks if there are any medications that can help.
Image by Monstera, retrieved from: https://www.pexels.com/photo/islamic-african-american-female-in-hijab-near-wall-6282045/ on March 31, 2022. Image is in the public domain

## Slide 16
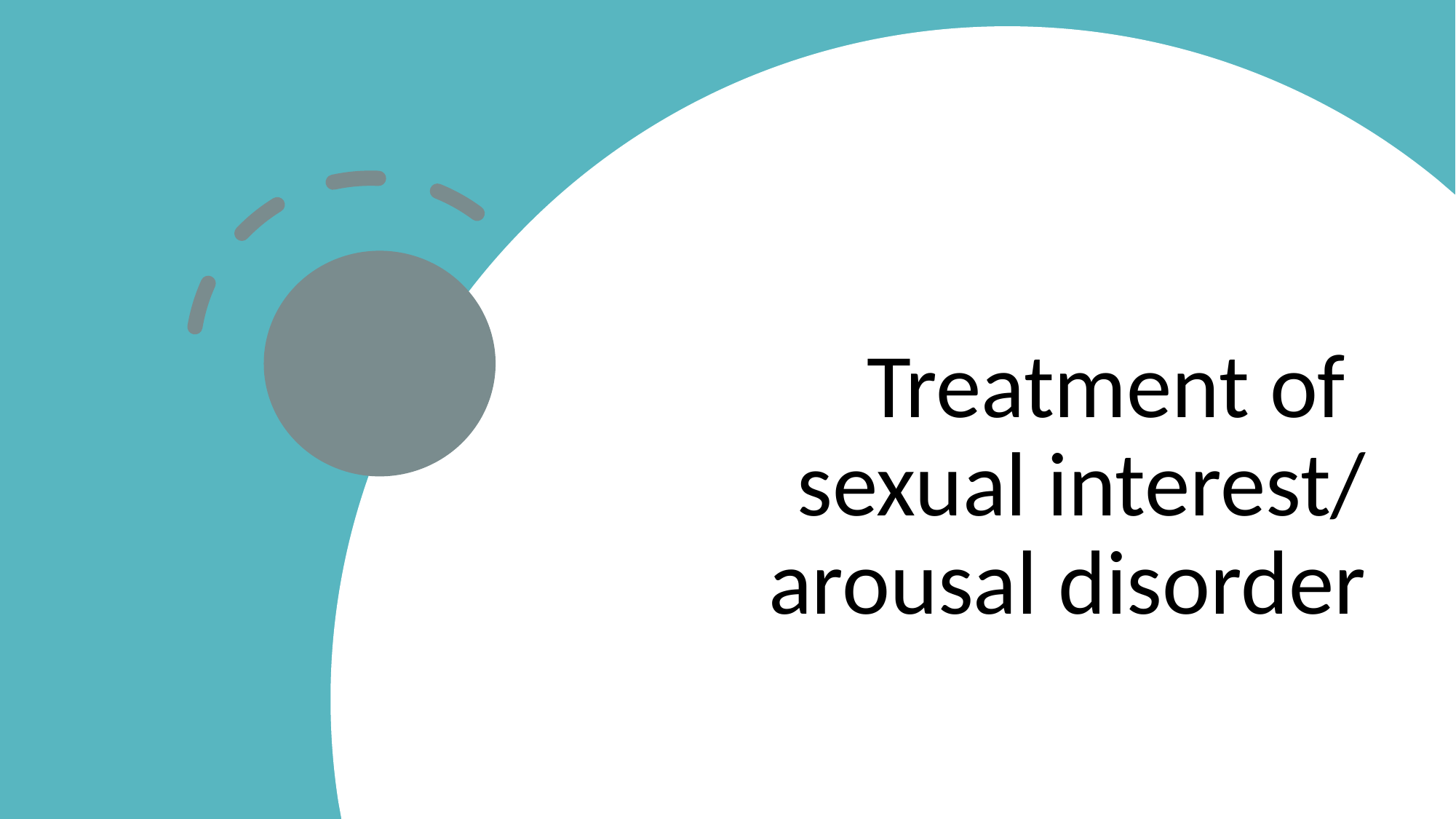

# Treatment of  sexual interest/ arousal disorder

## Slide 17
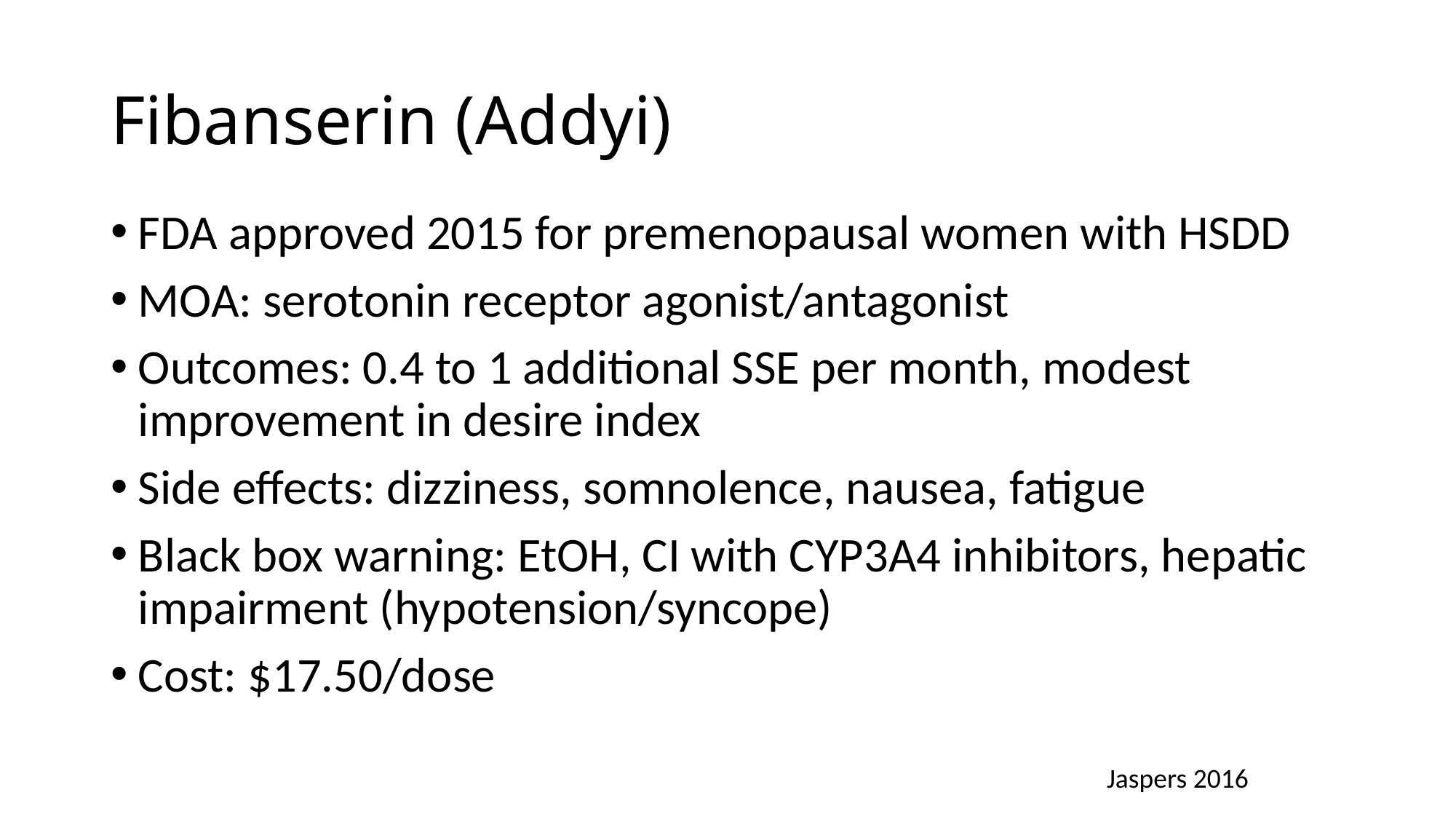

# Fibanserin (Addyi)
FDA approved 2015 for premenopausal women with HSDD
MOA: serotonin receptor agonist/antagonist
Outcomes: 0.4 to 1 additional SSE per month, modest improvement in desire index
Side effects: dizziness, somnolence, nausea, fatigue
Black box warning: EtOH, CI with CYP3A4 inhibitors, hepatic impairment (hypotension/syncope)
Cost: $17.50/dose
Jaspers 2016

## Slide 18
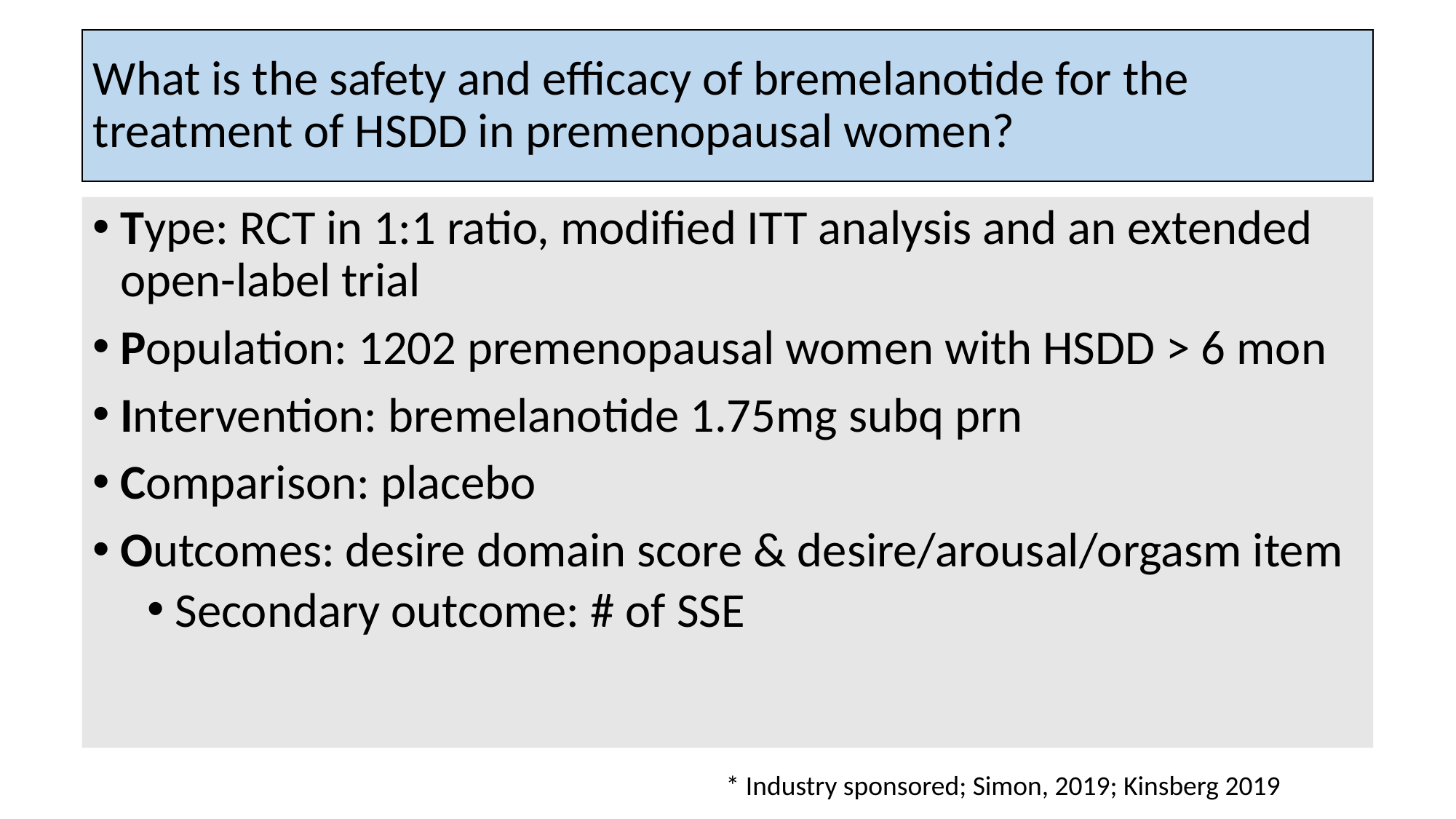

What is the safety and efficacy of bremelanotide for the treatment of HSDD in premenopausal women?
# Bremalenotide
Type: RCT in 1:1 ratio, modified ITT analysis and an extended open-label trial
Population: 1202 premenopausal women with HSDD > 6 mon
Intervention: bremelanotide 1.75mg subq prn
Comparison: placebo
Outcomes: desire domain score & desire/arousal/orgasm item
Secondary outcome: # of SSE
* Industry sponsored; Simon, 2019; Kinsberg 2019

## Slide 19
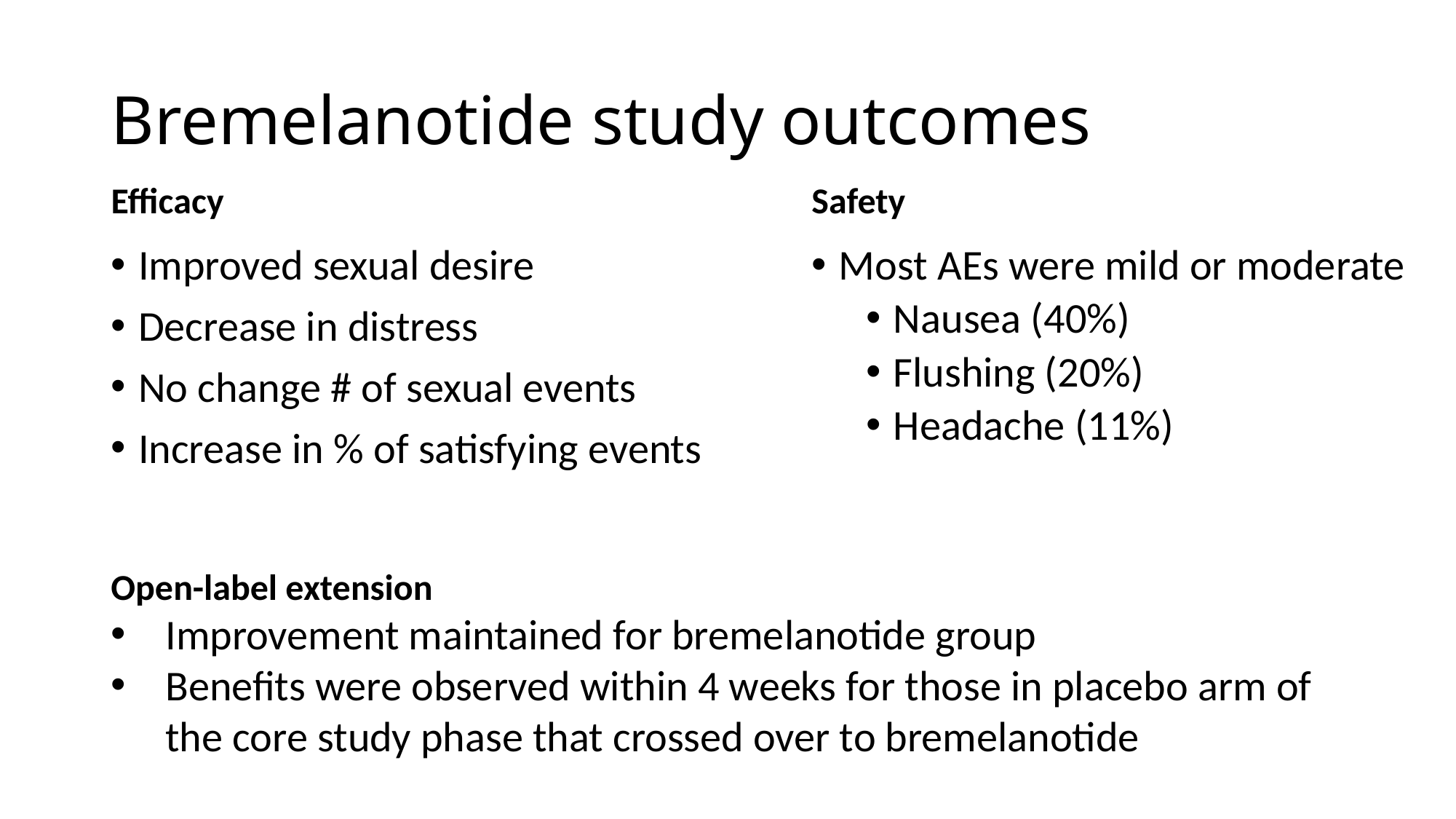

# Bremelanotide study outcomes
Efficacy
Safety
Improved sexual desire
Decrease in distress
No change # of sexual events
Increase in % of satisfying events
Most AEs were mild or moderate
Nausea (40%)
Flushing (20%)
Headache (11%)
Open-label extension
Improvement maintained for bremelanotide group
Benefits were observed within 4 weeks for those in placebo arm of the core study phase that crossed over to bremelanotide

## Slide 20
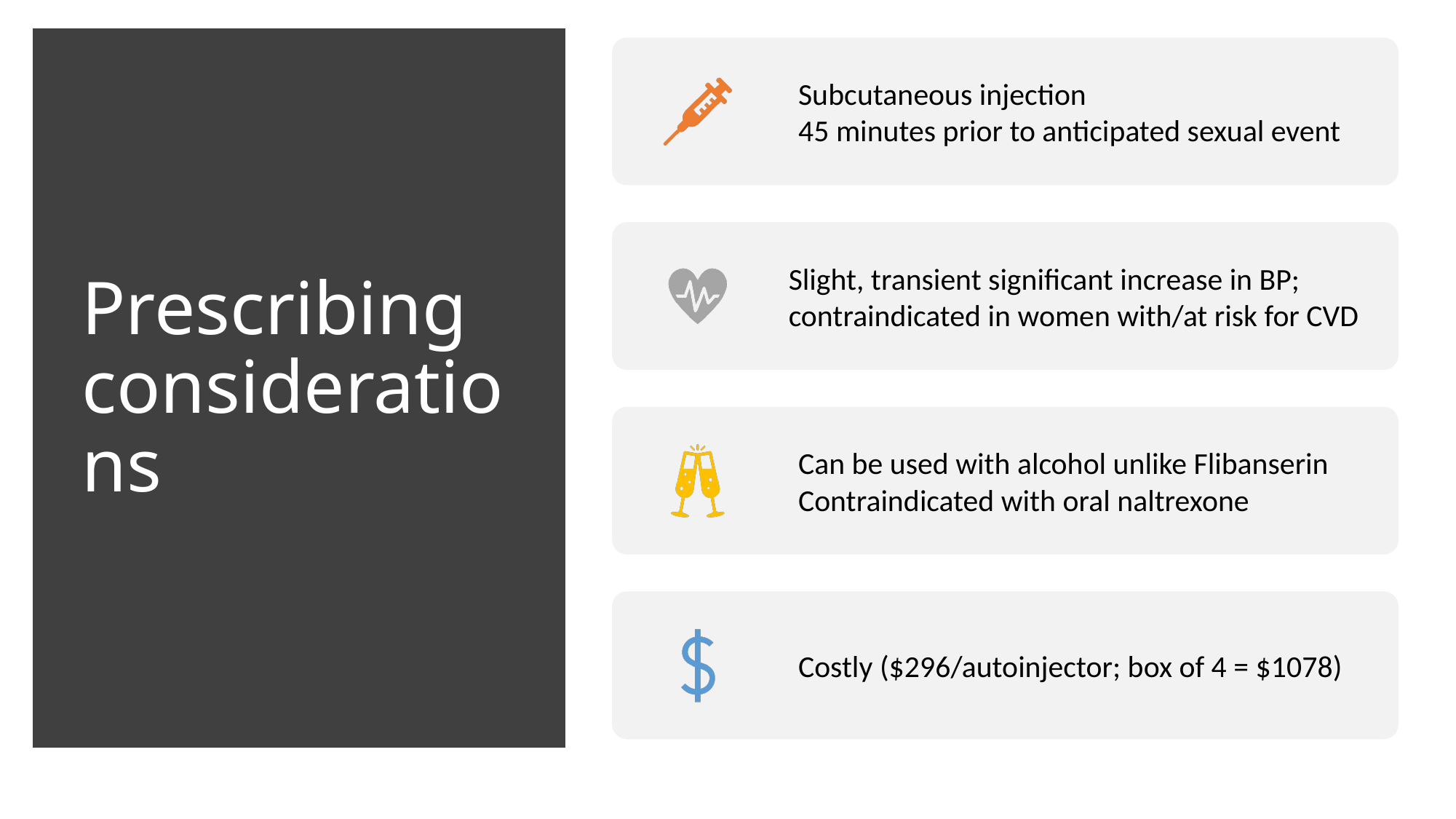

# Prescribing considerations

## Slide 21
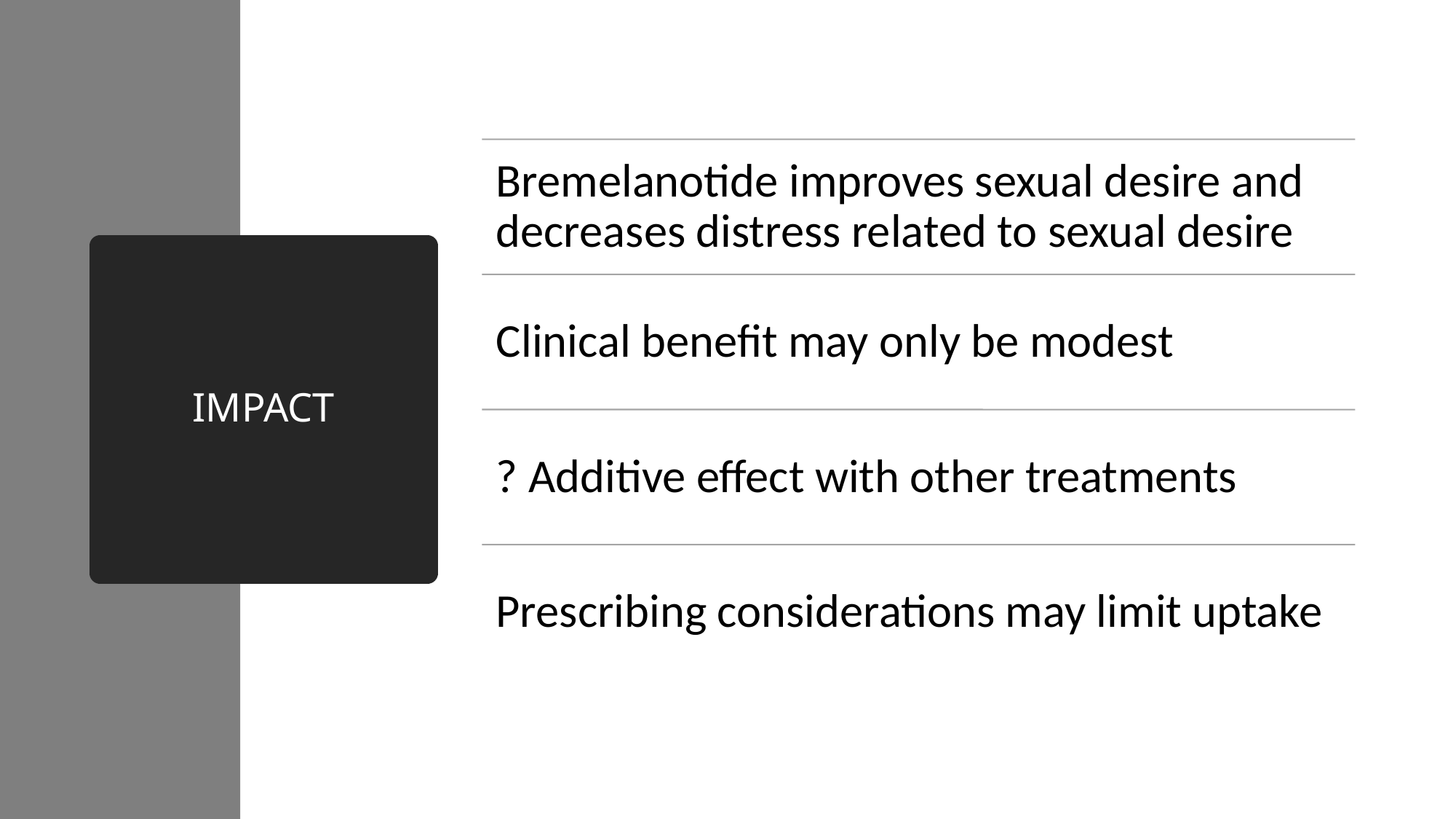

# IMPACT

## Slide 22
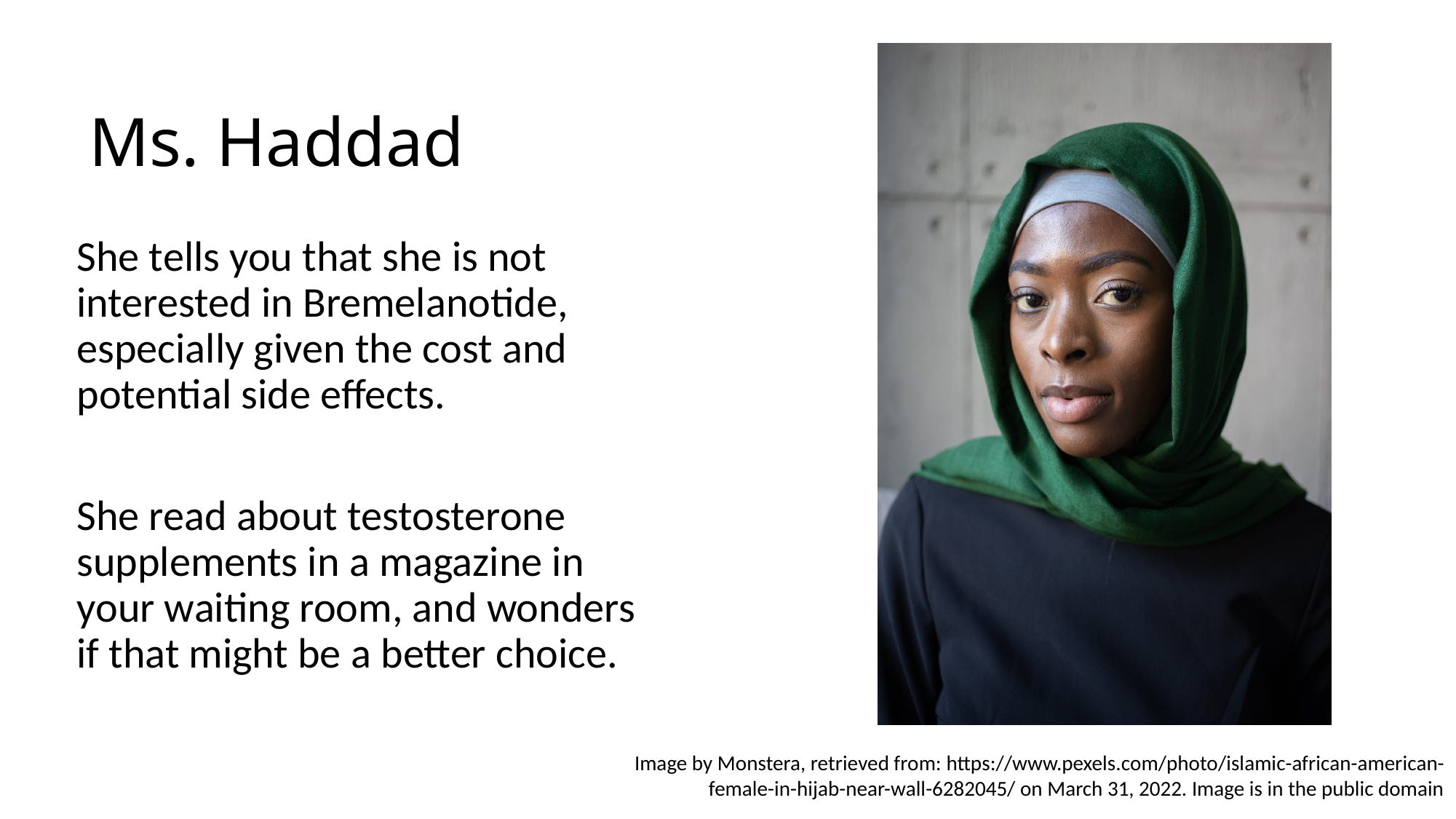

# Ms. Haddad
She tells you that she is not interested in Bremelanotide, especially given the cost and potential side effects.
She read about testosterone supplements in a magazine in your waiting room, and wonders if that might be a better choice.
Image by Monstera, retrieved from: https://www.pexels.com/photo/islamic-african-american-female-in-hijab-near-wall-6282045/ on March 31, 2022. Image is in the public domain

## Slide 23
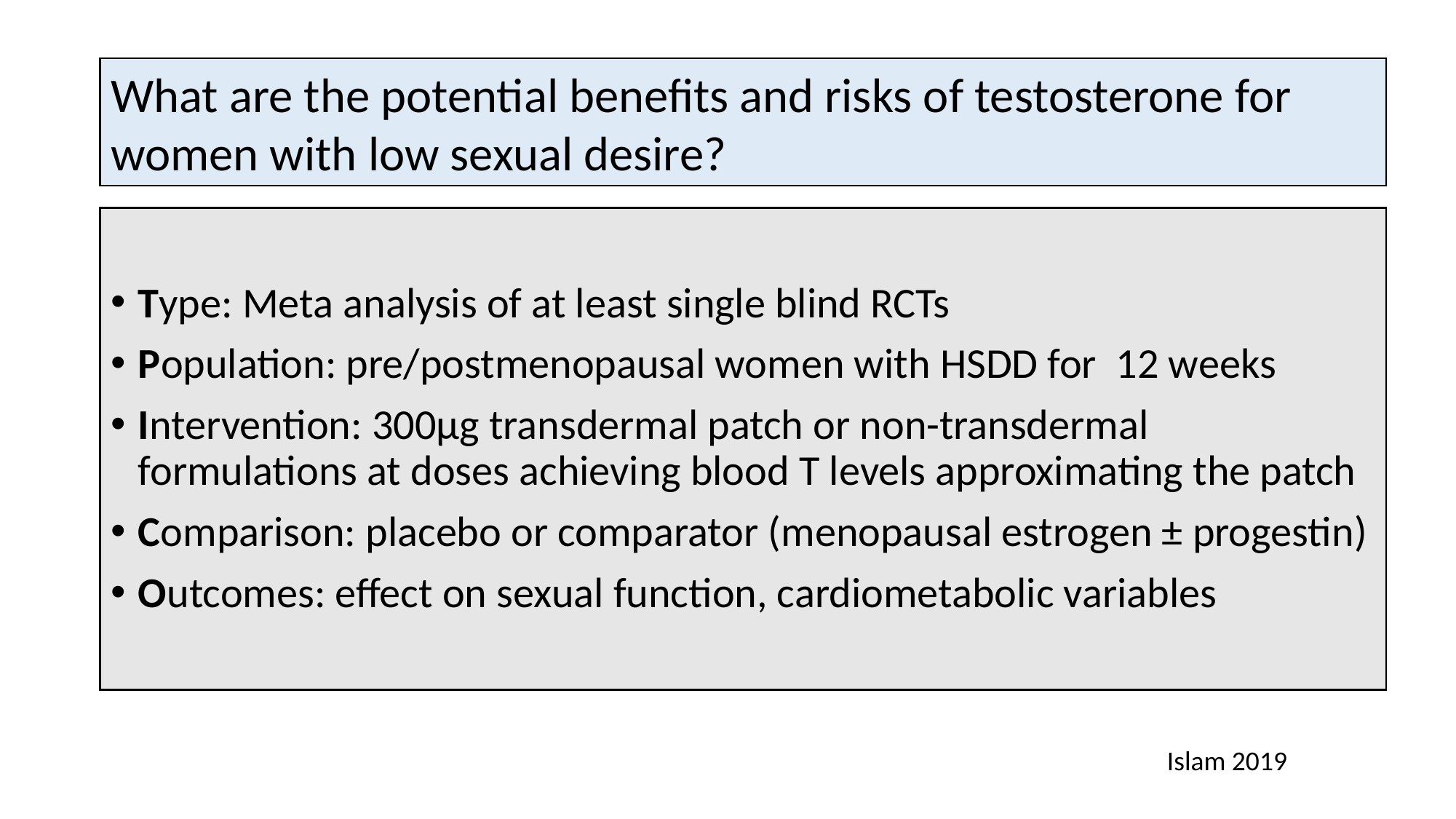

# Testosterone
What are the potential benefits and risks of testosterone for women with low sexual desire?
Islam 2019

## Slide 24
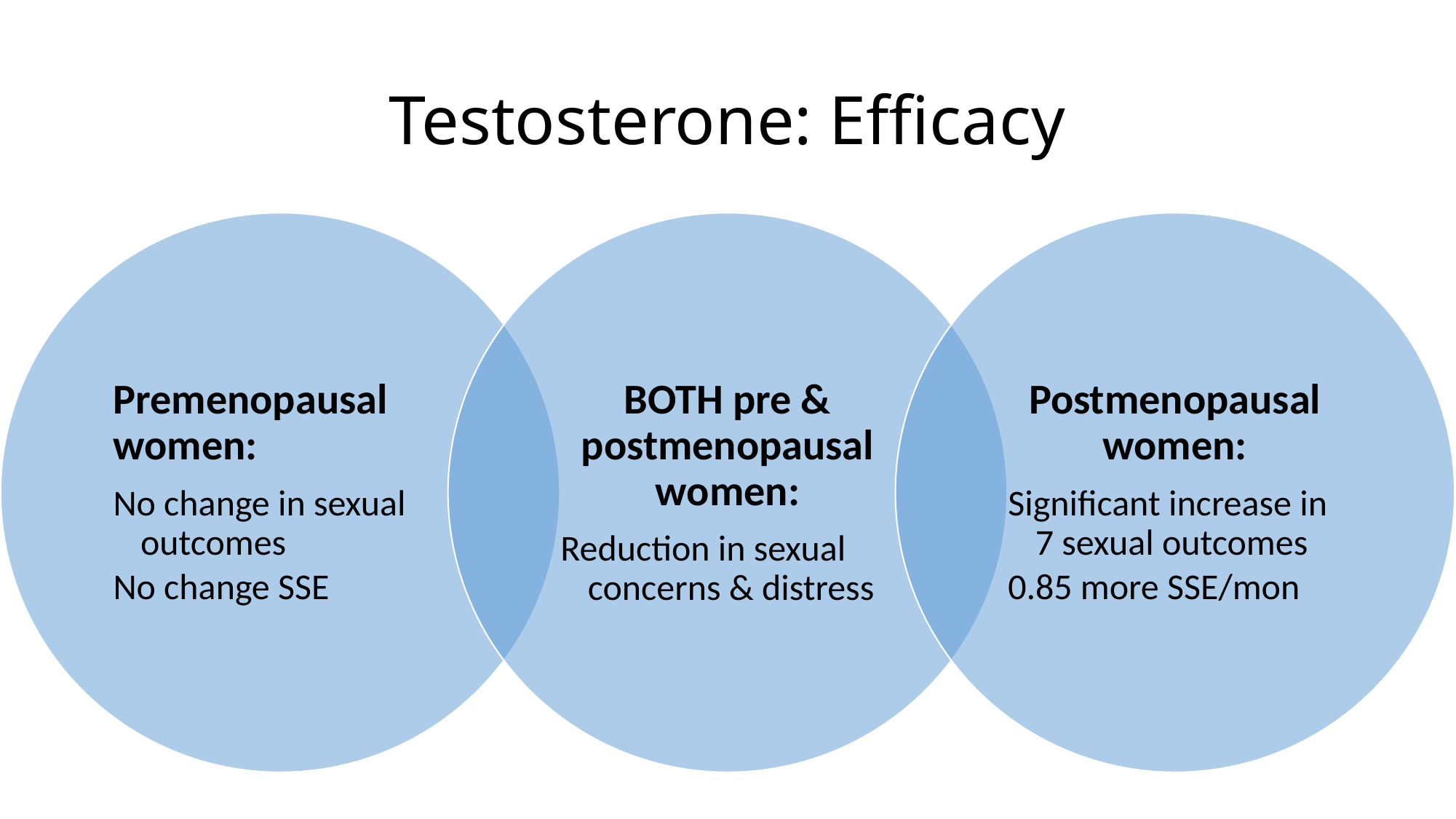

# Testosterone: Efficacy

## Slide 25
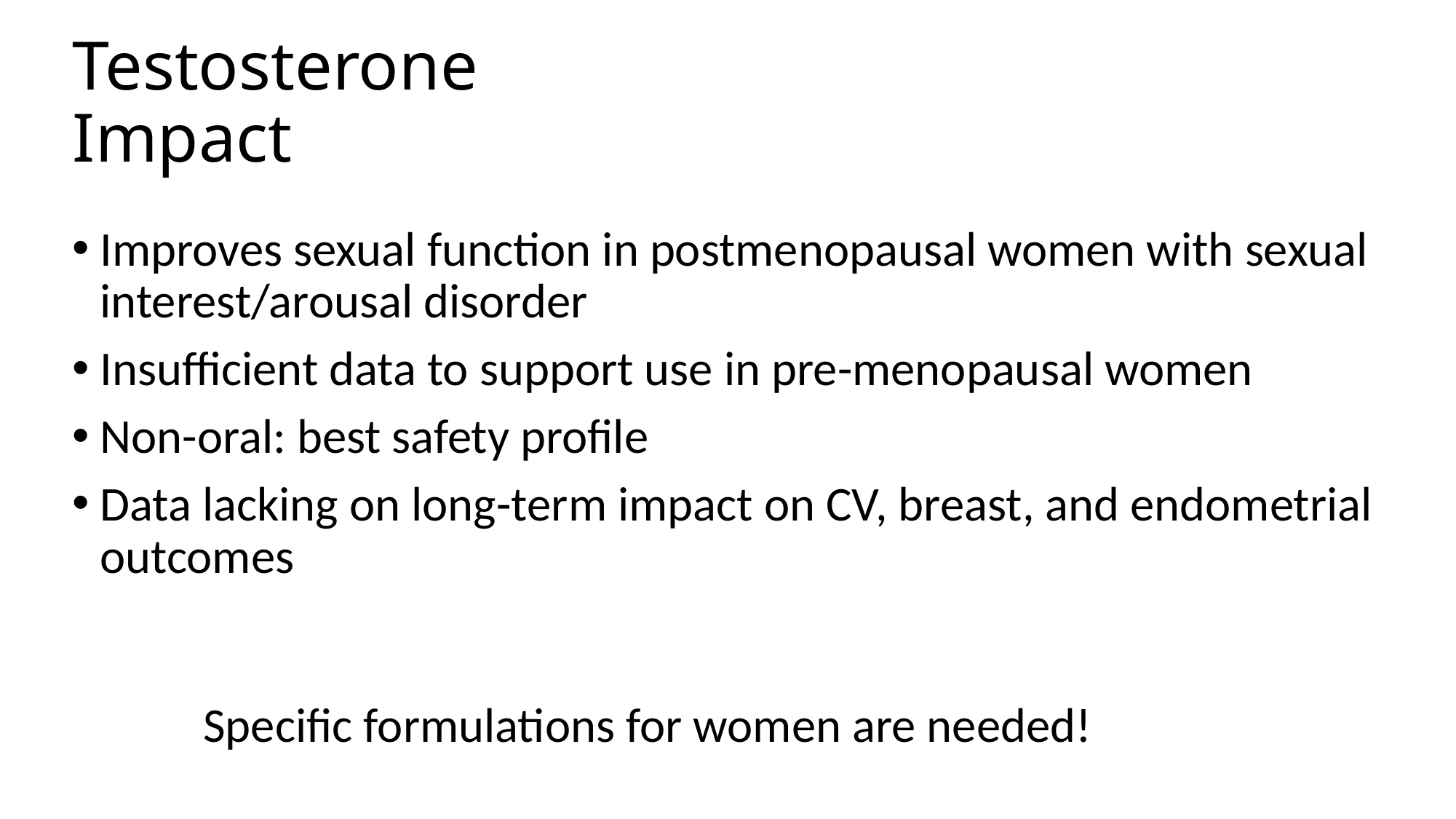

# Testosterone Impact
Improves sexual function in postmenopausal women with sexual interest/arousal disorder
Insufficient data to support use in pre-menopausal women
Non-oral: best safety profile
Data lacking on long-term impact on CV, breast, and endometrial outcomes
Specific formulations for women are needed!

## Slide 26
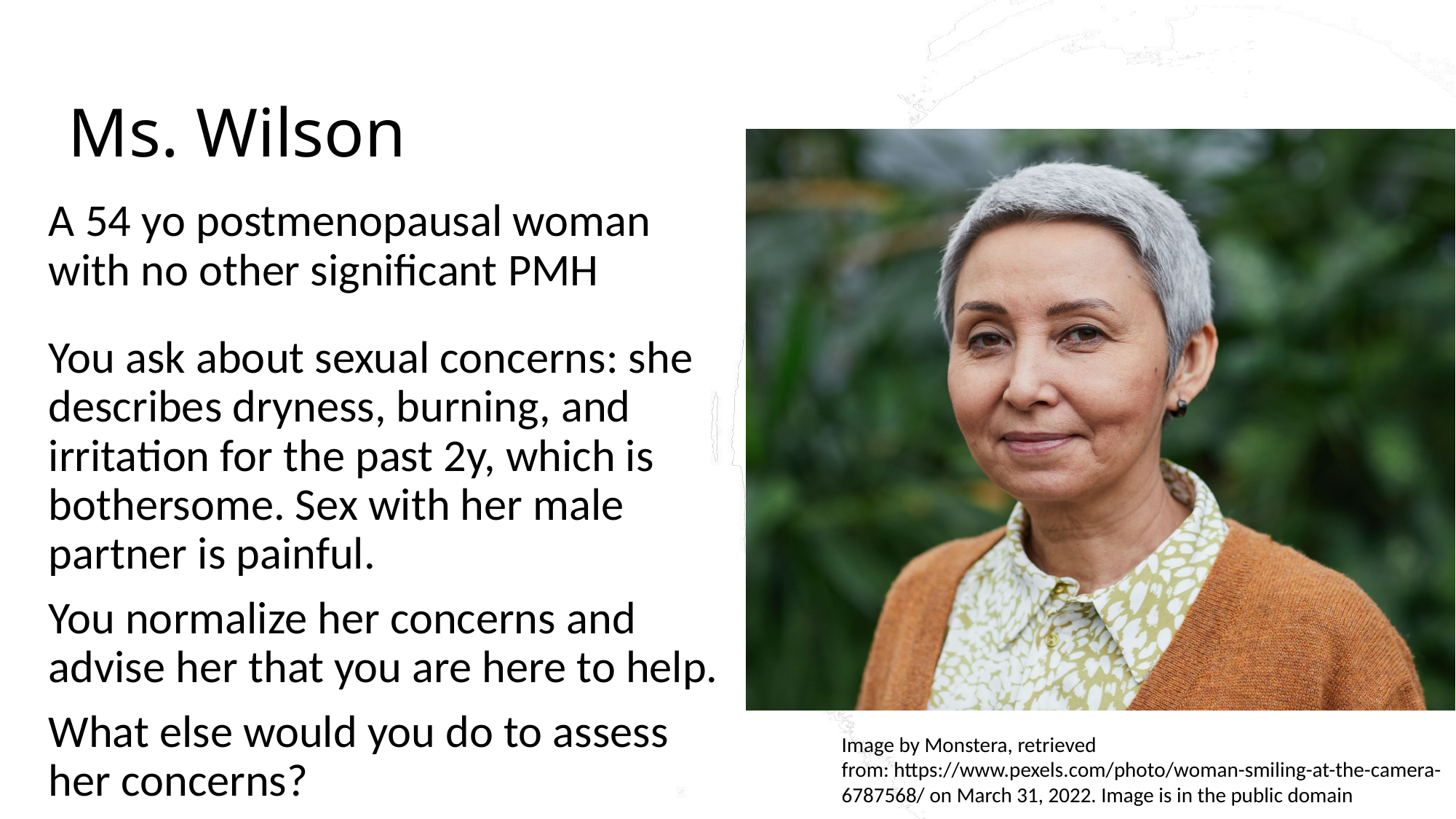

# Ms. Wilson
A 54 yo postmenopausal woman with no other significant PMH
You ask about sexual concerns: she describes dryness, burning, and irritation for the past 2y, which is bothersome. Sex with her male partner is painful.
You normalize her concerns and advise her that you are here to help.
What else would you do to assess her concerns?
Image by Monstera, retrieved from: https://www.pexels.com/photo/woman-smiling-at-the-camera-6787568/ on March 31, 2022. Image is in the public domain

## Slide 27
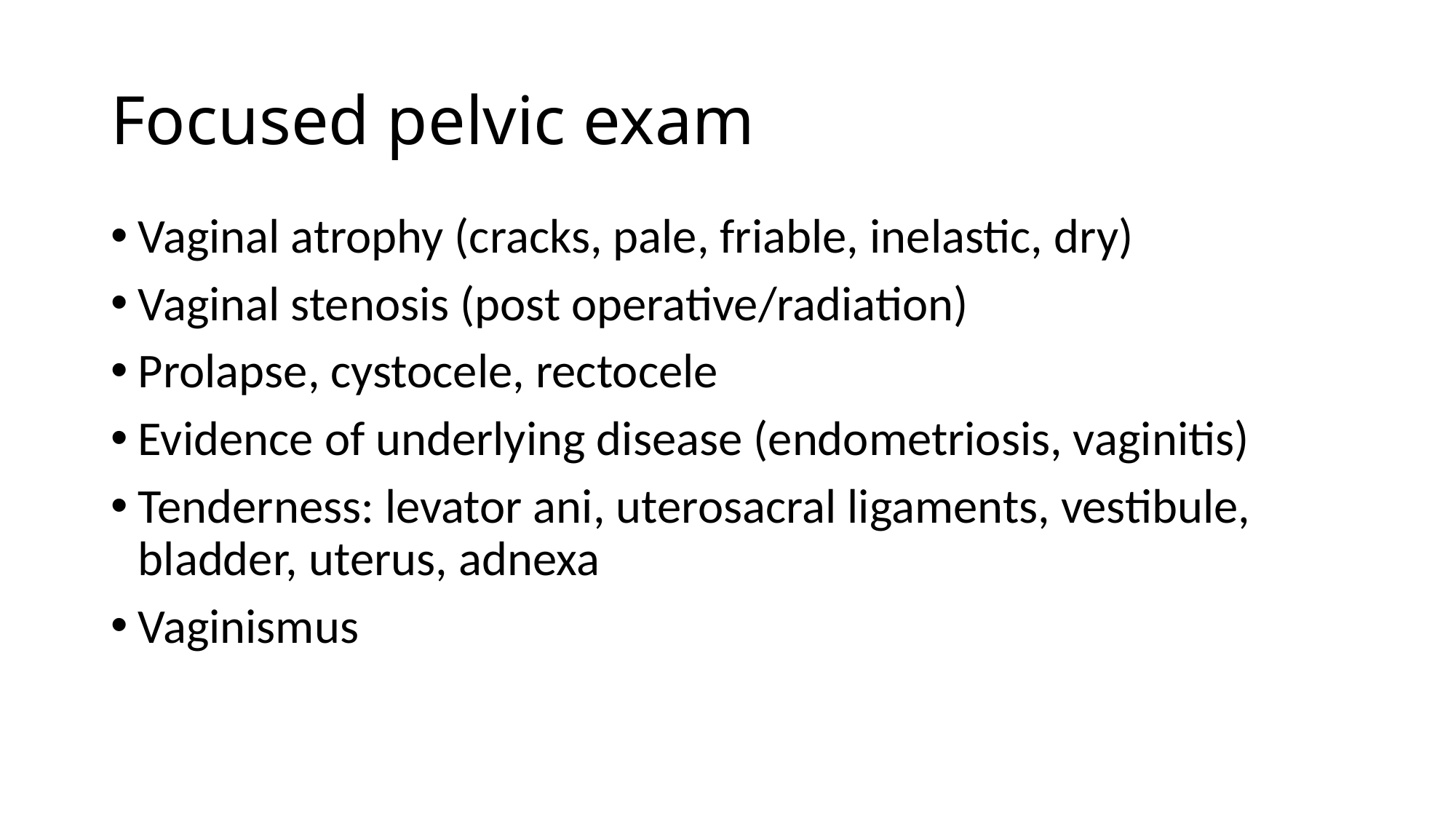

# Focused pelvic exam
Vaginal atrophy (cracks, pale, friable, inelastic, dry)
Vaginal stenosis (post operative/radiation)
Prolapse, cystocele, rectocele
Evidence of underlying disease (endometriosis, vaginitis)
Tenderness: levator ani, uterosacral ligaments, vestibule, bladder, uterus, adnexa
Vaginismus

## Slide 28
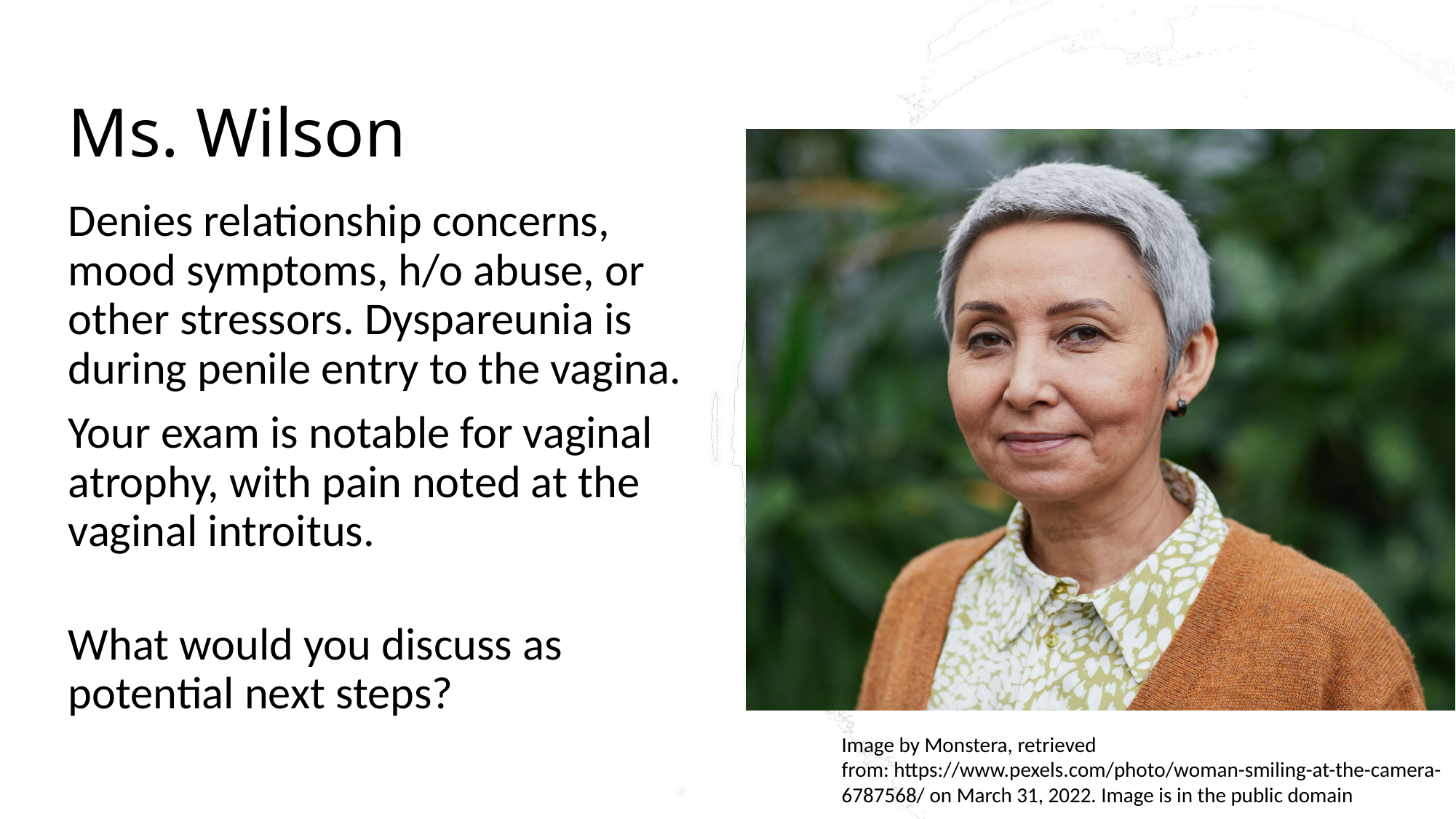

# Ms. Wilson
Denies relationship concerns, mood symptoms, h/o abuse, or other stressors. Dyspareunia is during penile entry to the vagina.
Your exam is notable for vaginal atrophy, with pain noted at the vaginal introitus.
What would you discuss as potential next steps?
Image by Monstera, retrieved from: https://www.pexels.com/photo/woman-smiling-at-the-camera-6787568/ on March 31, 2022. Image is in the public domain

## Slide 29
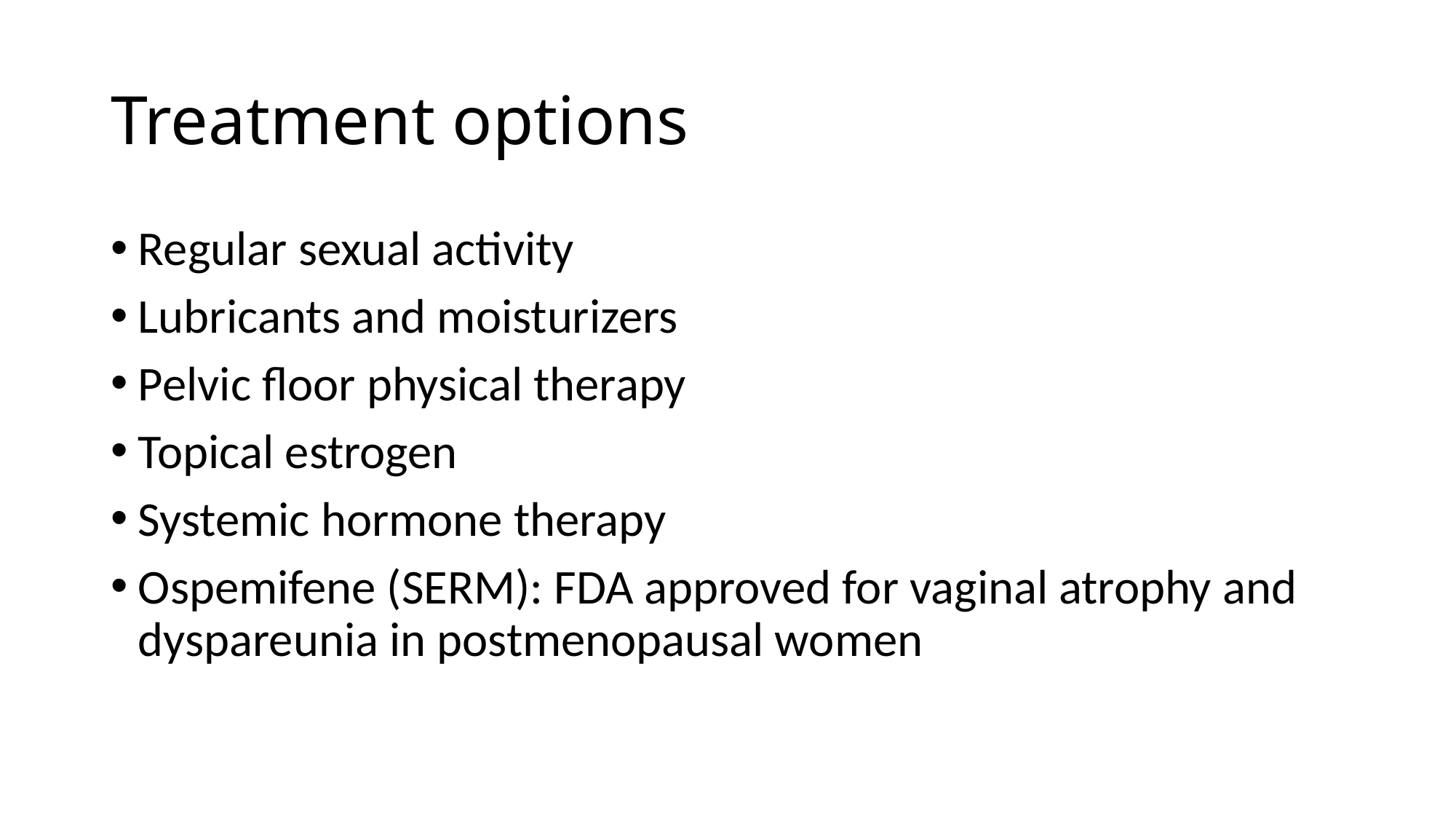

# Treatment options
Regular sexual activity
Lubricants and moisturizers
Pelvic floor physical therapy
Topical estrogen
Systemic hormone therapy
Ospemifene (SERM): FDA approved for vaginal atrophy and dyspareunia in postmenopausal women

## Slide 30
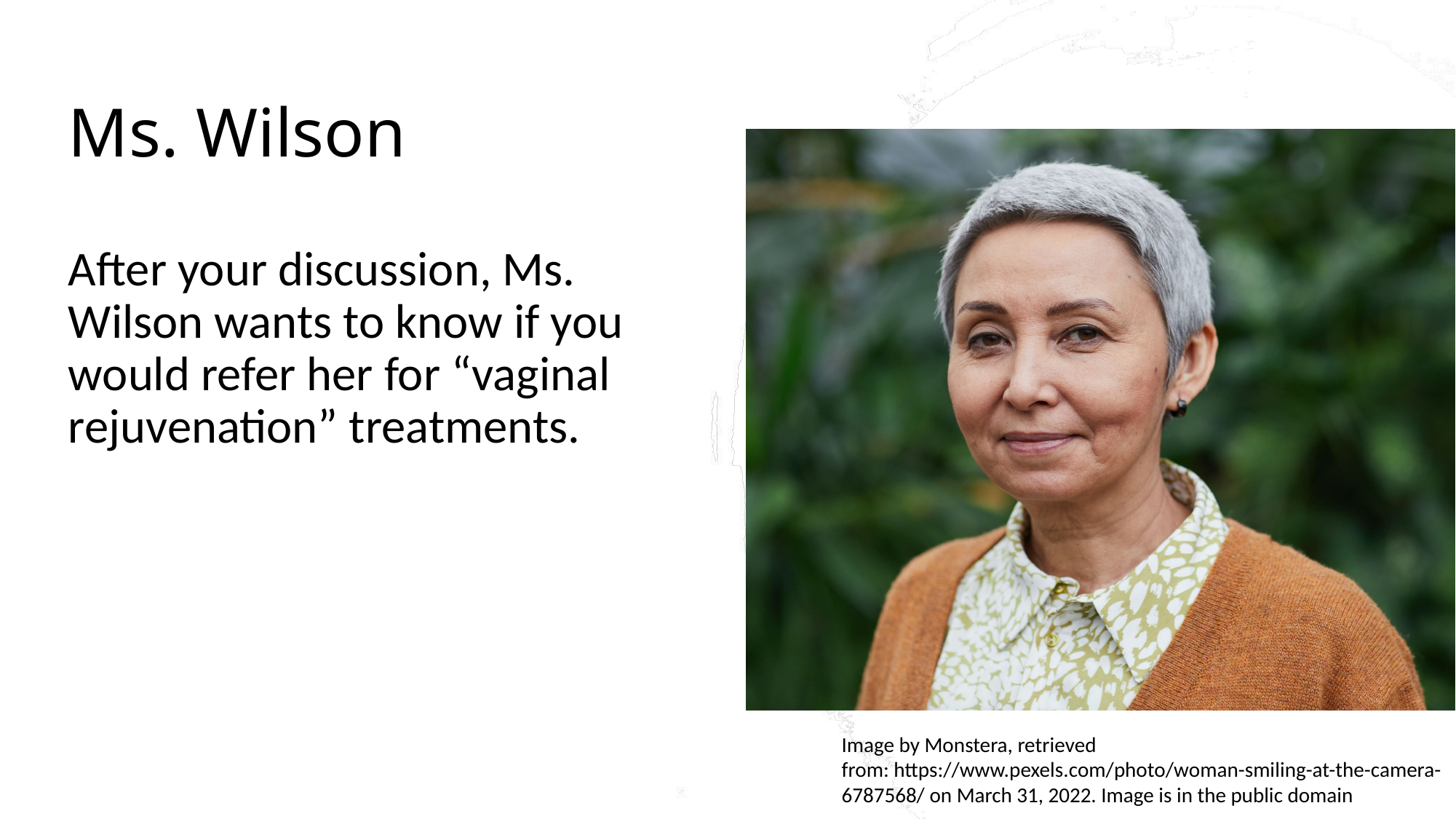

# Ms. Wilson
After your discussion, Ms. Wilson wants to know if you would refer her for “vaginal rejuvenation” treatments.
Image by Monstera, retrieved from: https://www.pexels.com/photo/woman-smiling-at-the-camera-6787568/ on March 31, 2022. Image is in the public domain

## Slide 31
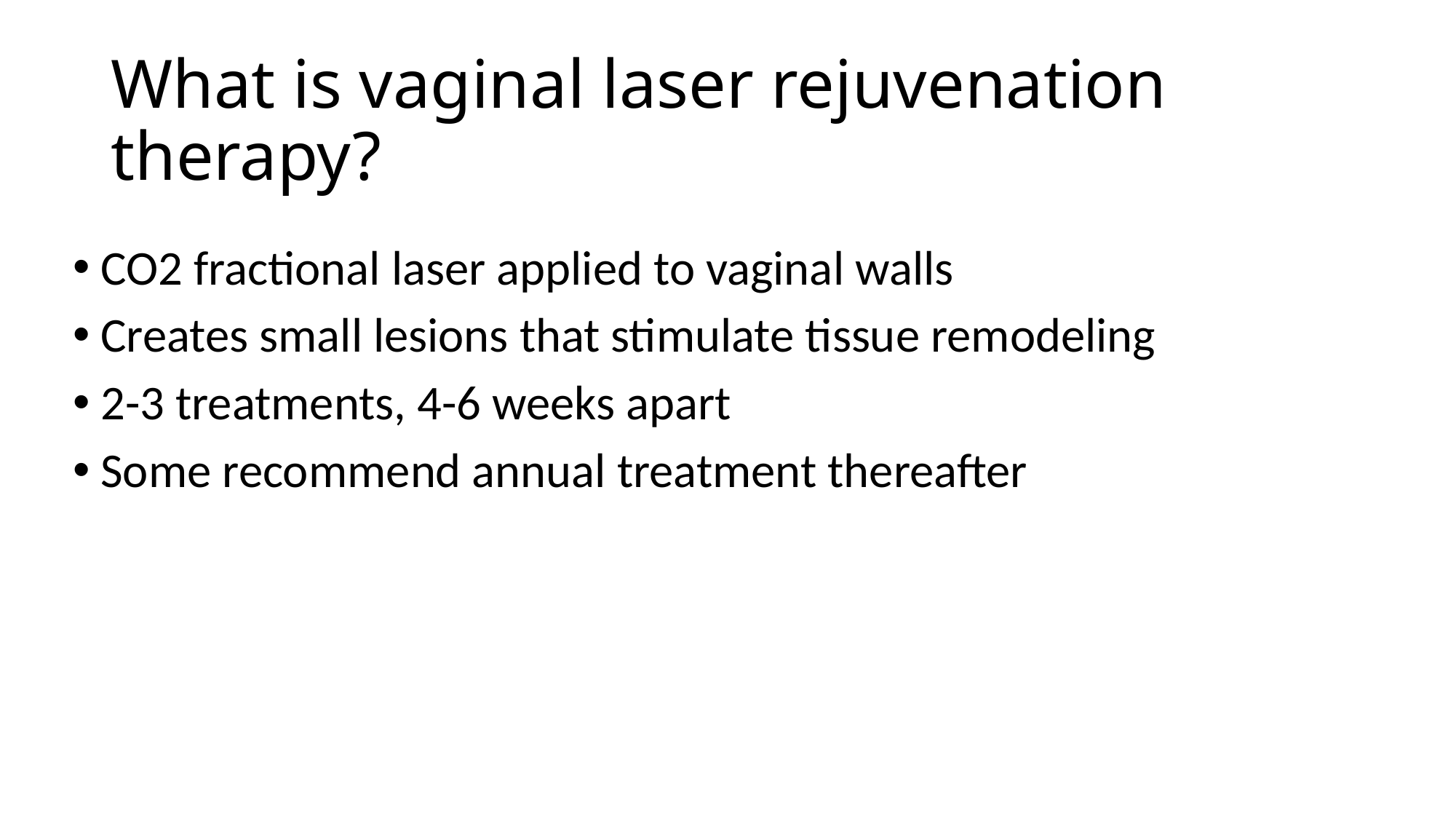

# What is vaginal laser rejuvenation therapy?
CO2 fractional laser applied to vaginal walls
Creates small lesions that stimulate tissue remodeling
2-3 treatments, 4-6 weeks apart
Some recommend annual treatment thereafter

## Slide 32
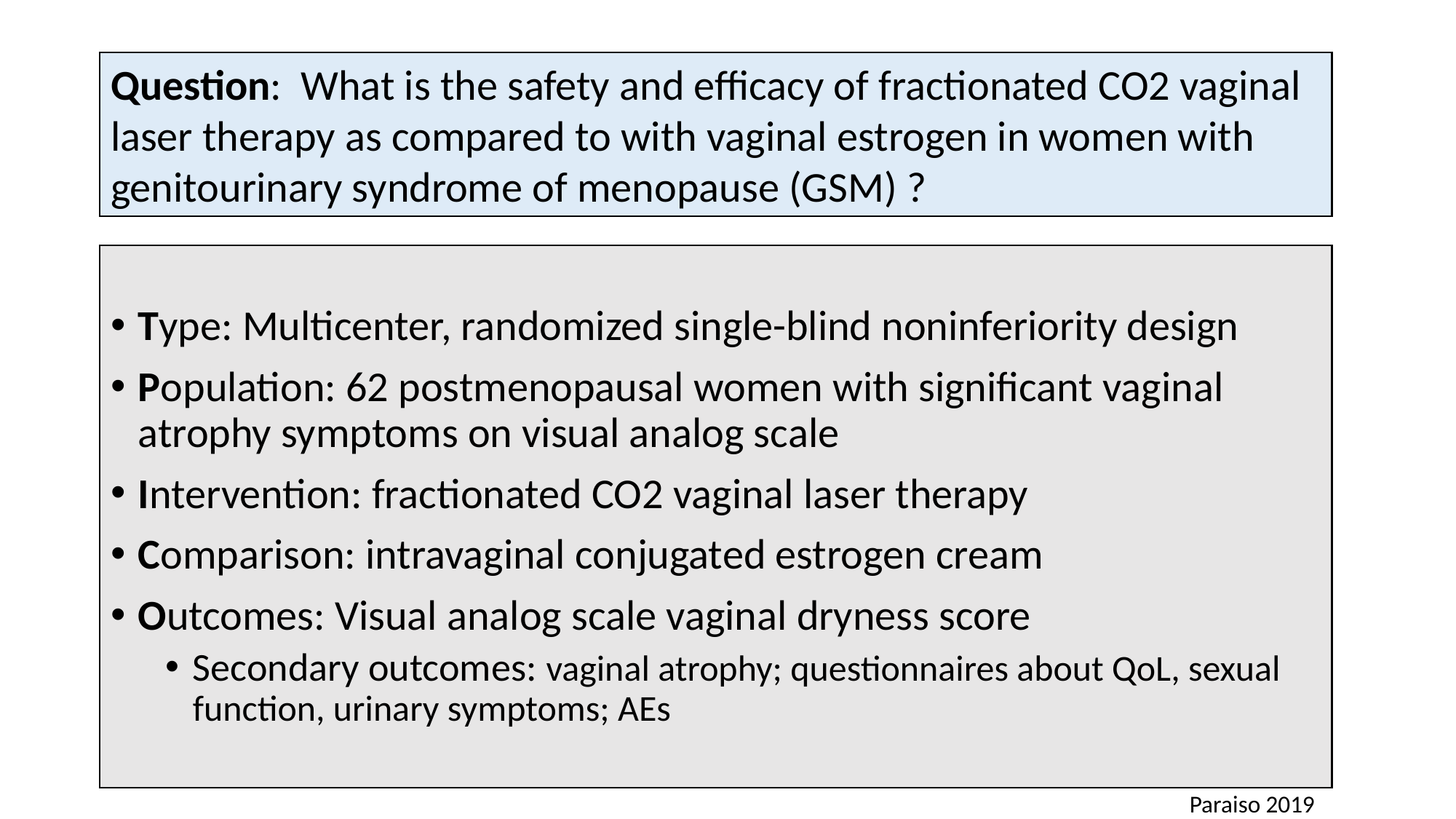

Question: What is the safety and efficacy of fractionated CO2 vaginal laser therapy as compared to with vaginal estrogen in women with genitourinary syndrome of menopause (GSM) ?
Type: Multicenter, randomized single-blind noninferiority design
Population: 62 postmenopausal women with significant vaginal atrophy symptoms on visual analog scale
Intervention: fractionated CO2 vaginal laser therapy
Comparison: intravaginal conjugated estrogen cream
Outcomes: Visual analog scale vaginal dryness score
Secondary outcomes: vaginal atrophy; questionnaires about QoL, sexual function, urinary symptoms; AEs
Paraiso 2019

## Slide 33
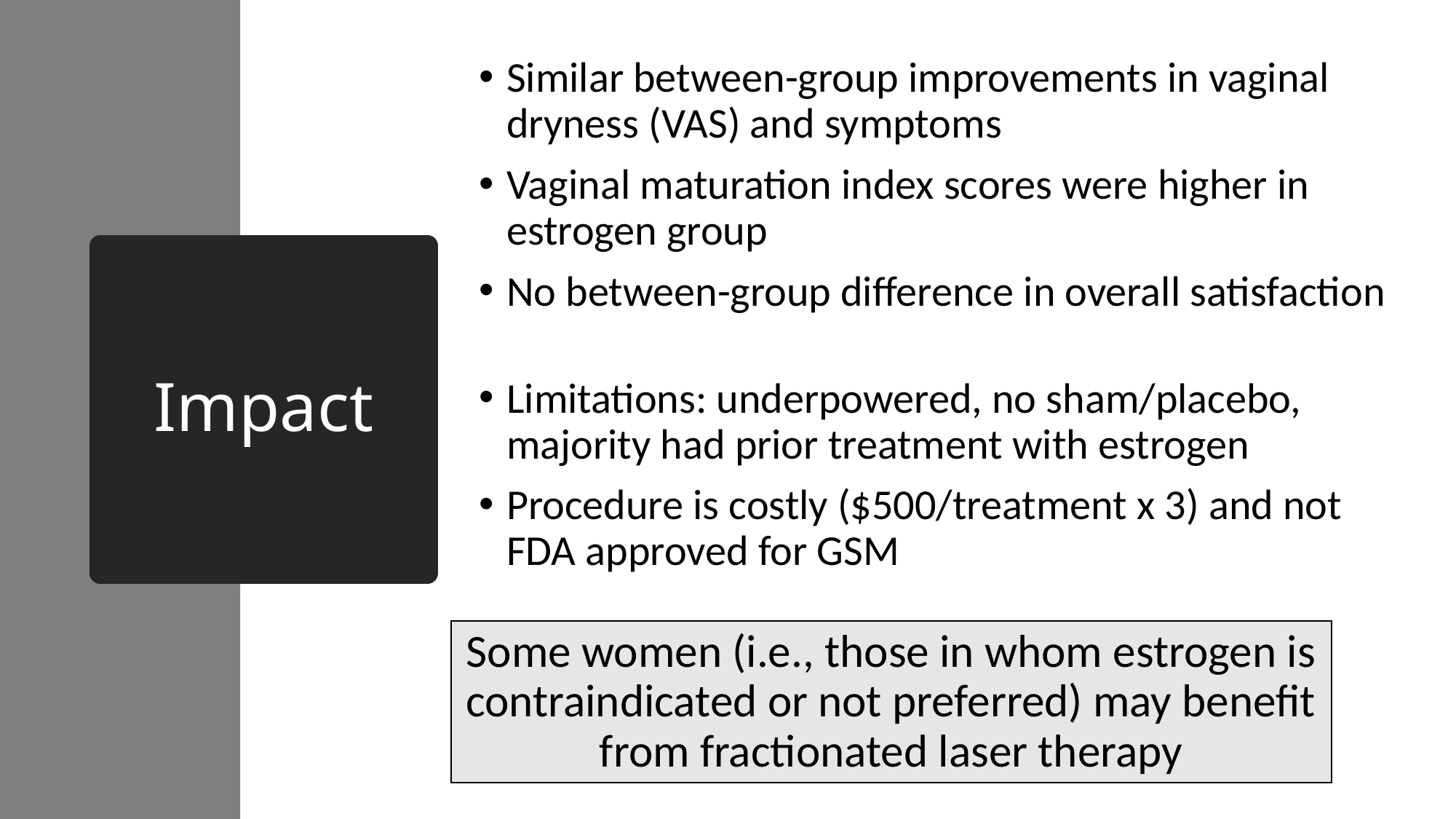

Similar between-group improvements in vaginal dryness (VAS) and symptoms
Vaginal maturation index scores were higher in estrogen group
No between-group difference in overall satisfaction
Limitations: underpowered, no sham/placebo, majority had prior treatment with estrogen
Procedure is costly ($500/treatment x 3) and not FDA approved for GSM
# Impact
Some women (i.e., those in whom estrogen is contraindicated or not preferred) may benefit from fractionated laser therapy

## Slide 34
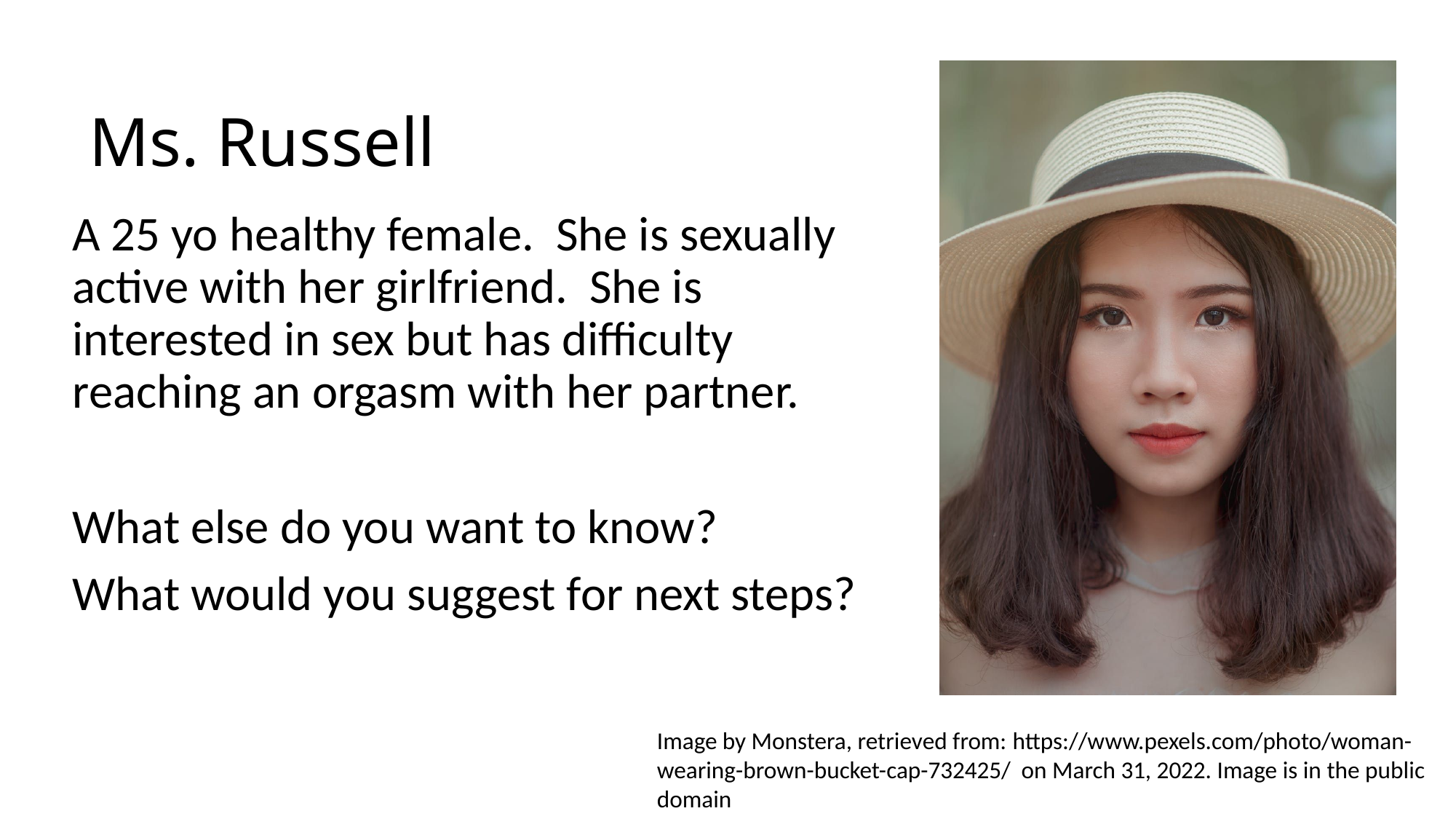

# Ms. Russell
A 25 yo healthy female. She is sexually active with her girlfriend. She is interested in sex but has difficulty reaching an orgasm with her partner.
What else do you want to know?
What would you suggest for next steps?
Image by Monstera, retrieved from: https://www.pexels.com/photo/woman-wearing-brown-bucket-cap-732425/ on March 31, 2022. Image is in the public domain

## Slide 35
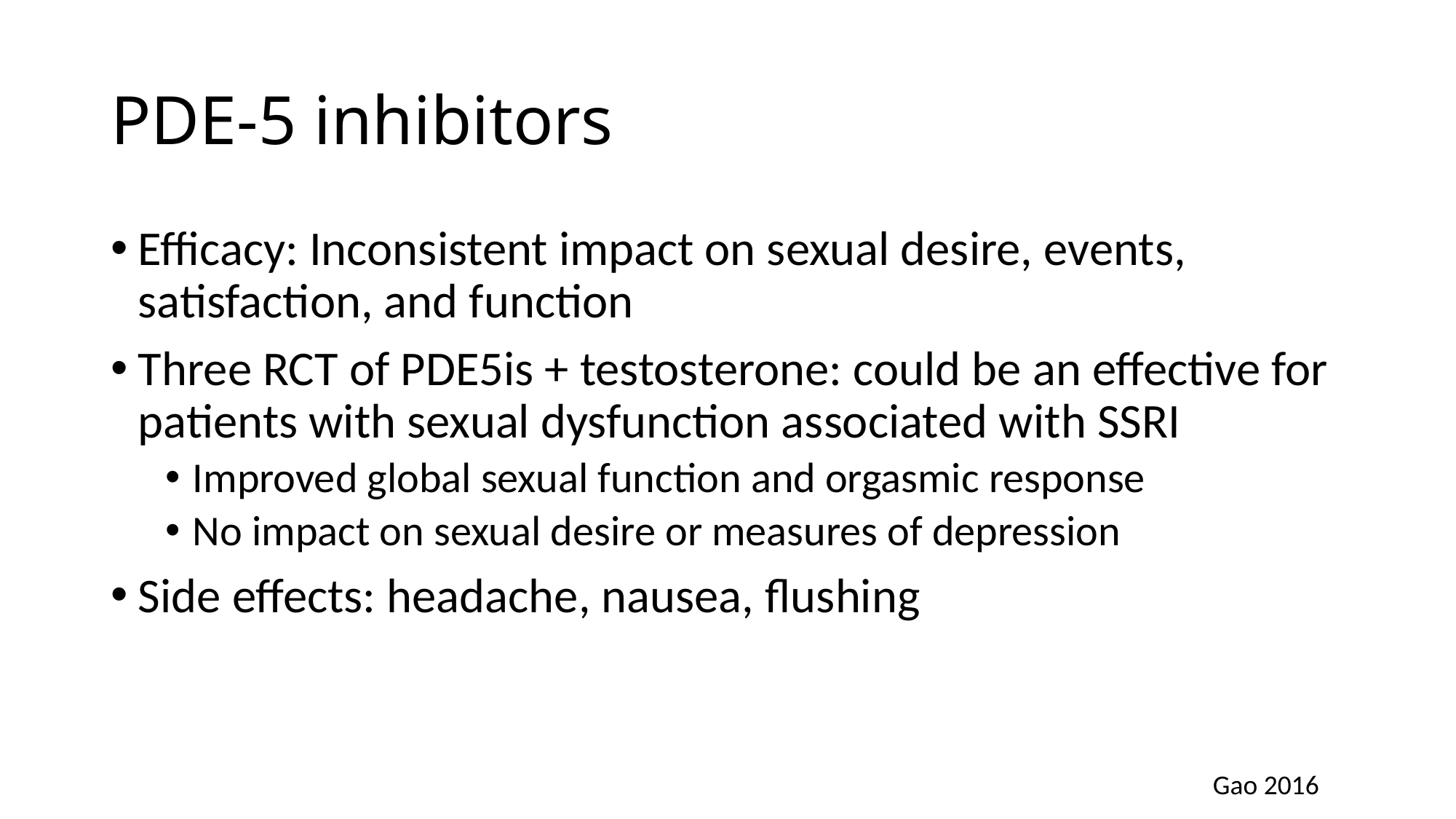

# PDE-5 inhibitors
Efficacy: Inconsistent impact on sexual desire, events, satisfaction, and function
Three RCT of PDE5is + testosterone: could be an effective for patients with sexual dysfunction associated with SSRI
Improved global sexual function and orgasmic response
No impact on sexual desire or measures of depression
Side effects: headache, nausea, flushing
Gao 2016

## Slide 36
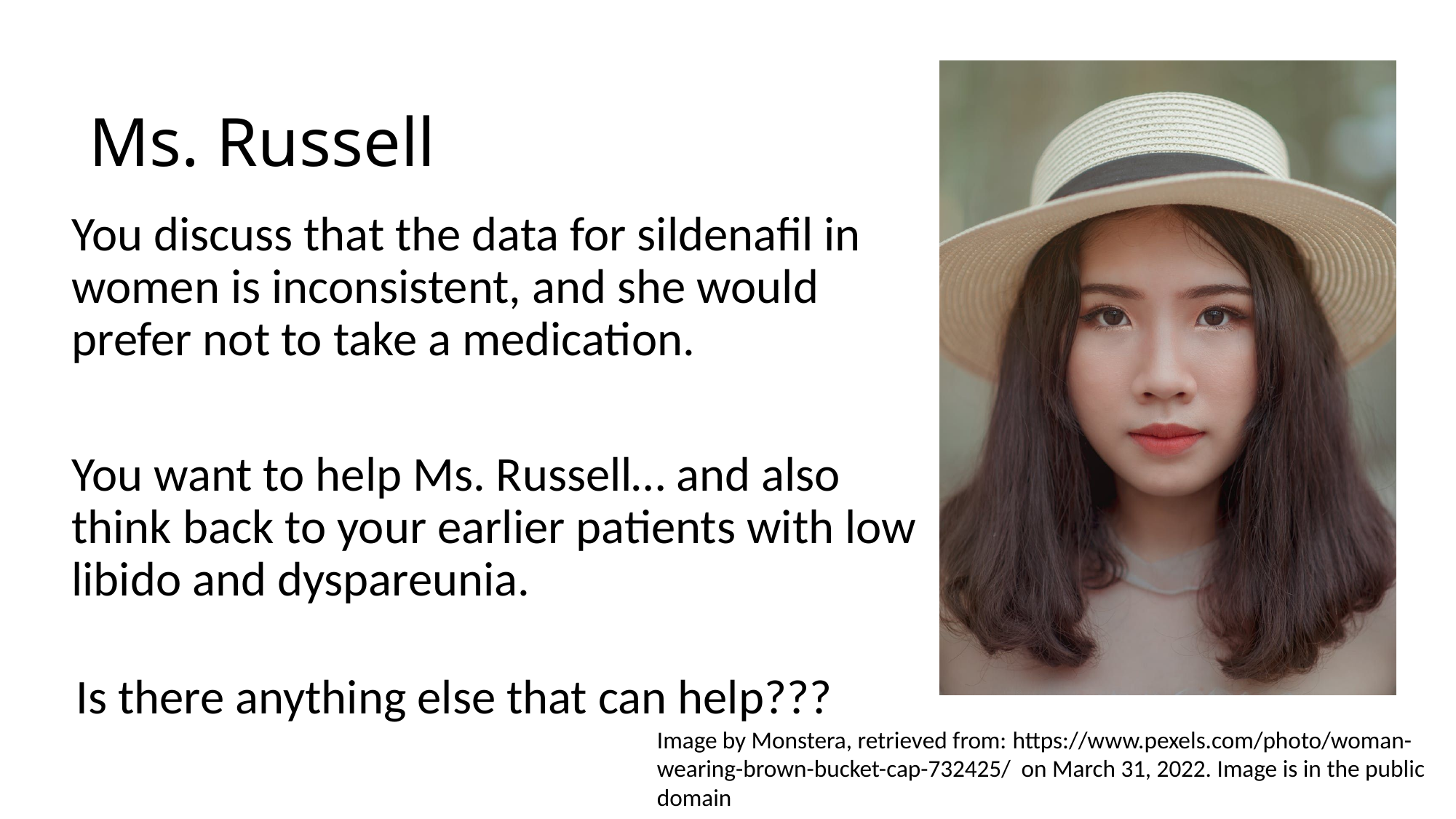

# Ms. Russell
You discuss that the data for sildenafil in women is inconsistent, and she would prefer not to take a medication.
You want to help Ms. Russell… and also think back to your earlier patients with low libido and dyspareunia.
Is there anything else that can help???
Image by Monstera, retrieved from: https://www.pexels.com/photo/woman-wearing-brown-bucket-cap-732425/ on March 31, 2022. Image is in the public domain

## Slide 37
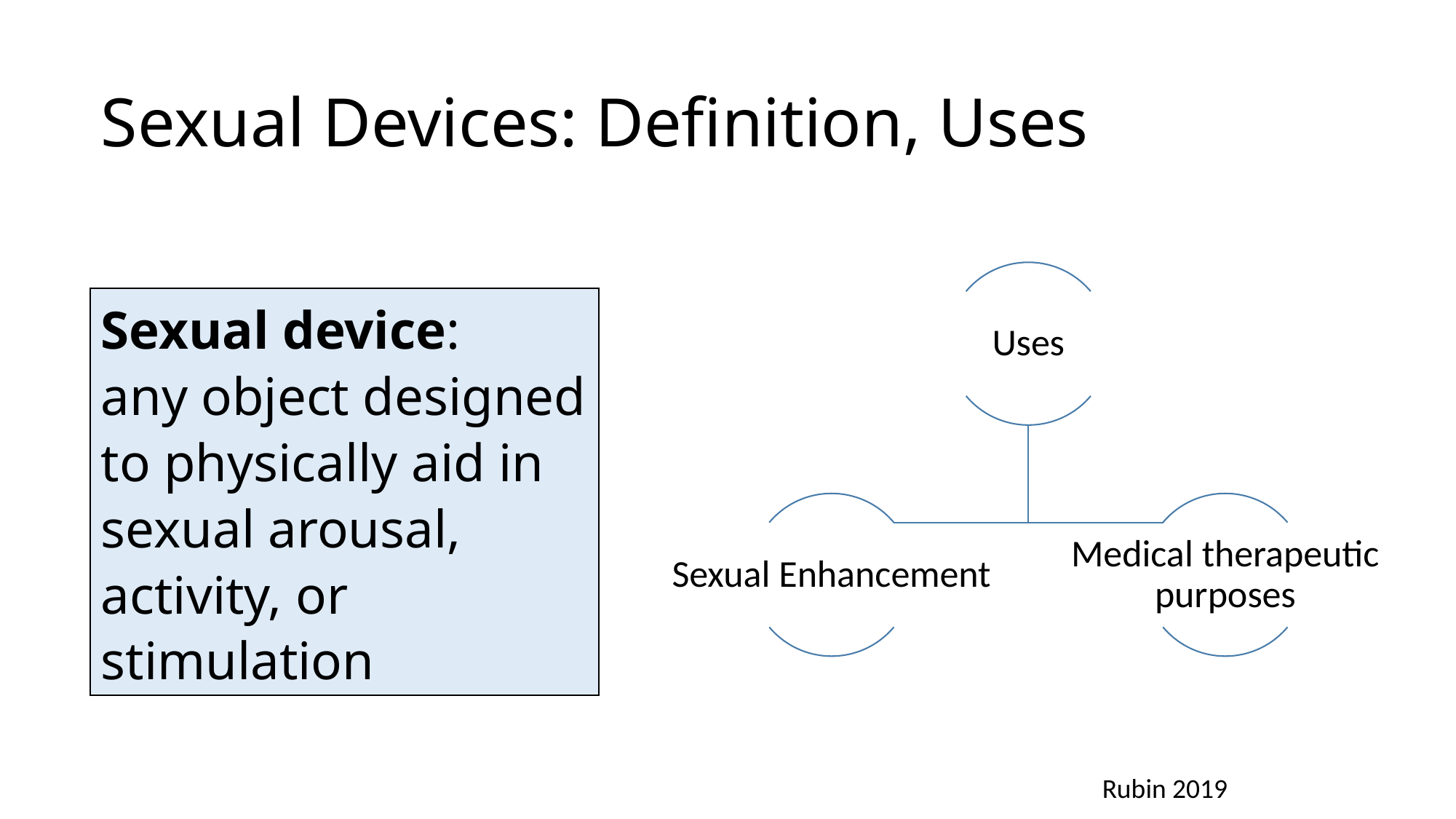

# Sexual Devices: Definition, Uses
Sexual device:
any object designed to physically aid in sexual arousal, activity, or stimulation
Rubin 2019

## Slide 38
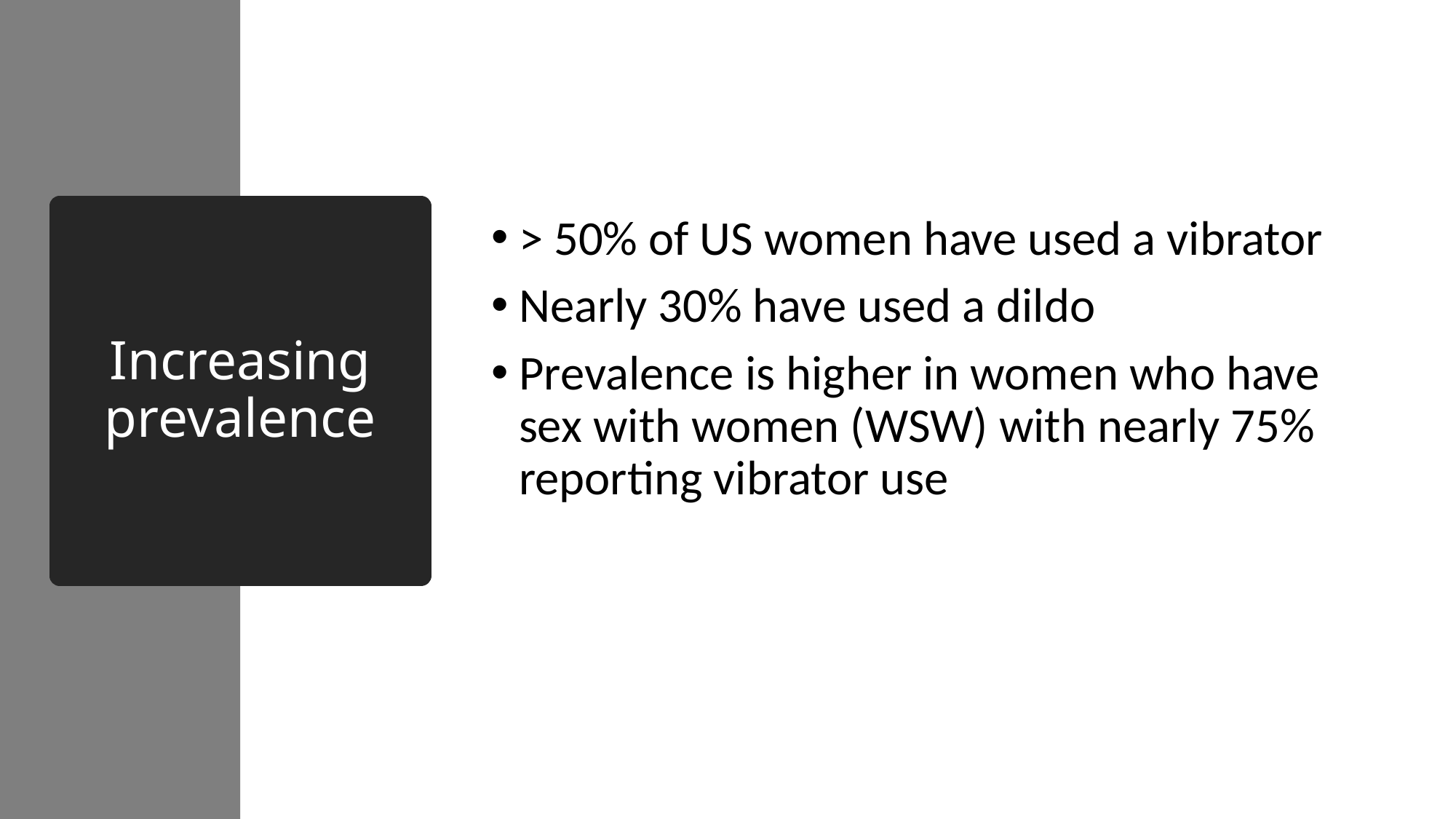

# Increasing prevalence
> 50% of US women have used a vibrator
Nearly 30% have used a dildo
Prevalence is higher in women who have sex with women (WSW) with nearly 75% reporting vibrator use

## Slide 39
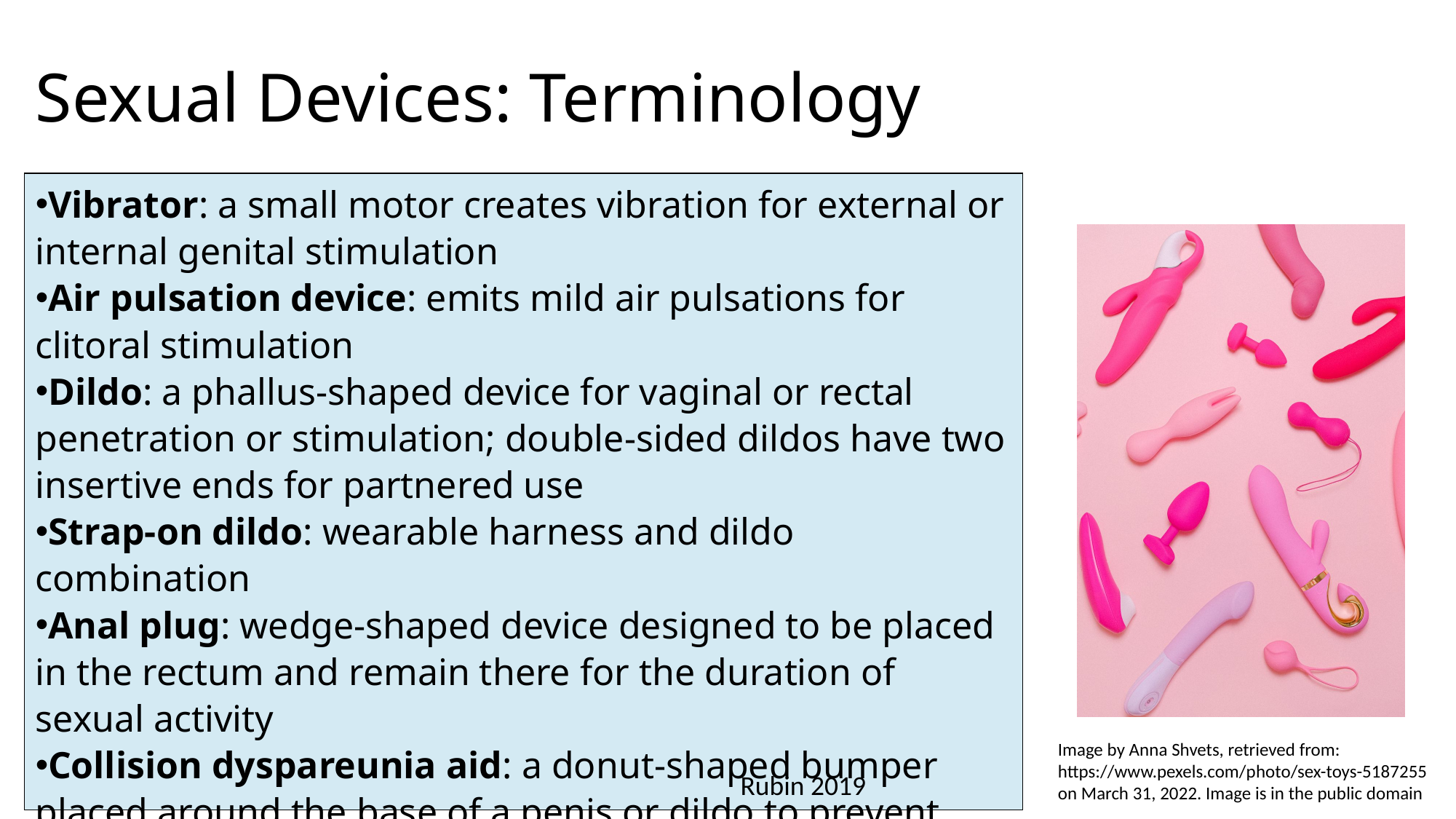

# Sexual Devices: Terminology
Vibrator: a small motor creates vibration for external or internal genital stimulation
Air pulsation device: emits mild air pulsations for clitoral stimulation
Dildo: a phallus-shaped device for vaginal or rectal penetration or stimulation; double-sided dildos have two insertive ends for partnered use
Strap-on dildo: wearable harness and dildo combination
Anal plug: wedge-shaped device designed to be placed in the rectum and remain there for the duration of sexual activity
Collision dyspareunia aid: a donut-shaped bumper placed around the base of a penis or dildo to prevent deep penetration; aka “penile ring”
Image by Anna Shvets, retrieved from: https://www.pexels.com/photo/sex-toys-5187255 on March 31, 2022. Image is in the public domain
Rubin 2019

## Slide 40
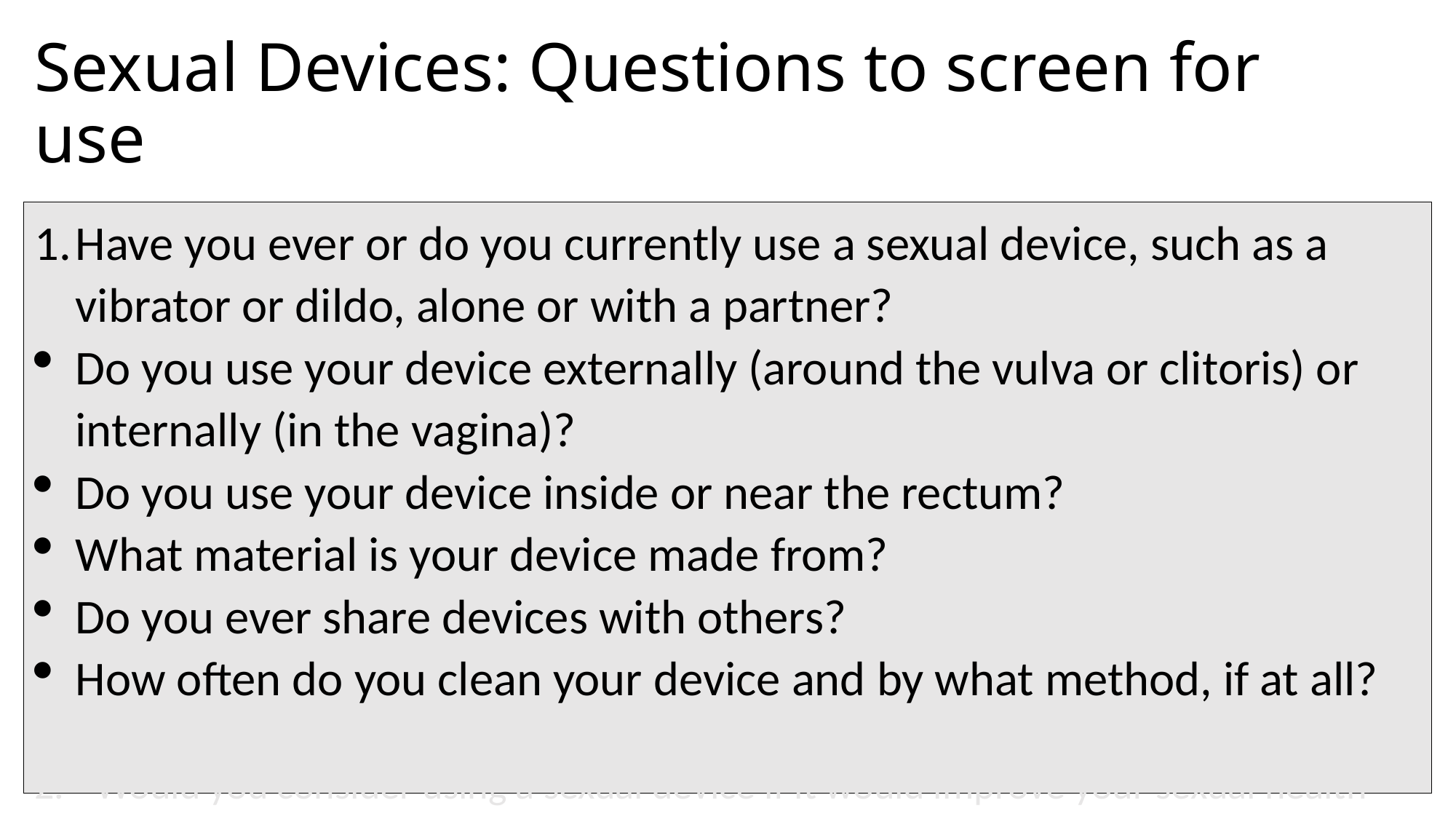

# Sexual Devices: Questions to screen for use
Have you ever or do you currently use a sexual device, such as a vibrator or dildo, alone or with a partner?
Do you use your device externally (around the vulva or clitoris) or internally (in the vagina)?
Do you use your device inside or near the rectum?
What material is your device made from?
Do you ever share devices with others?
How often do you clean your device and by what method, if at all?
Would you consider using a sexual device if it would improve your sexual health and wellness?

## Slide 41
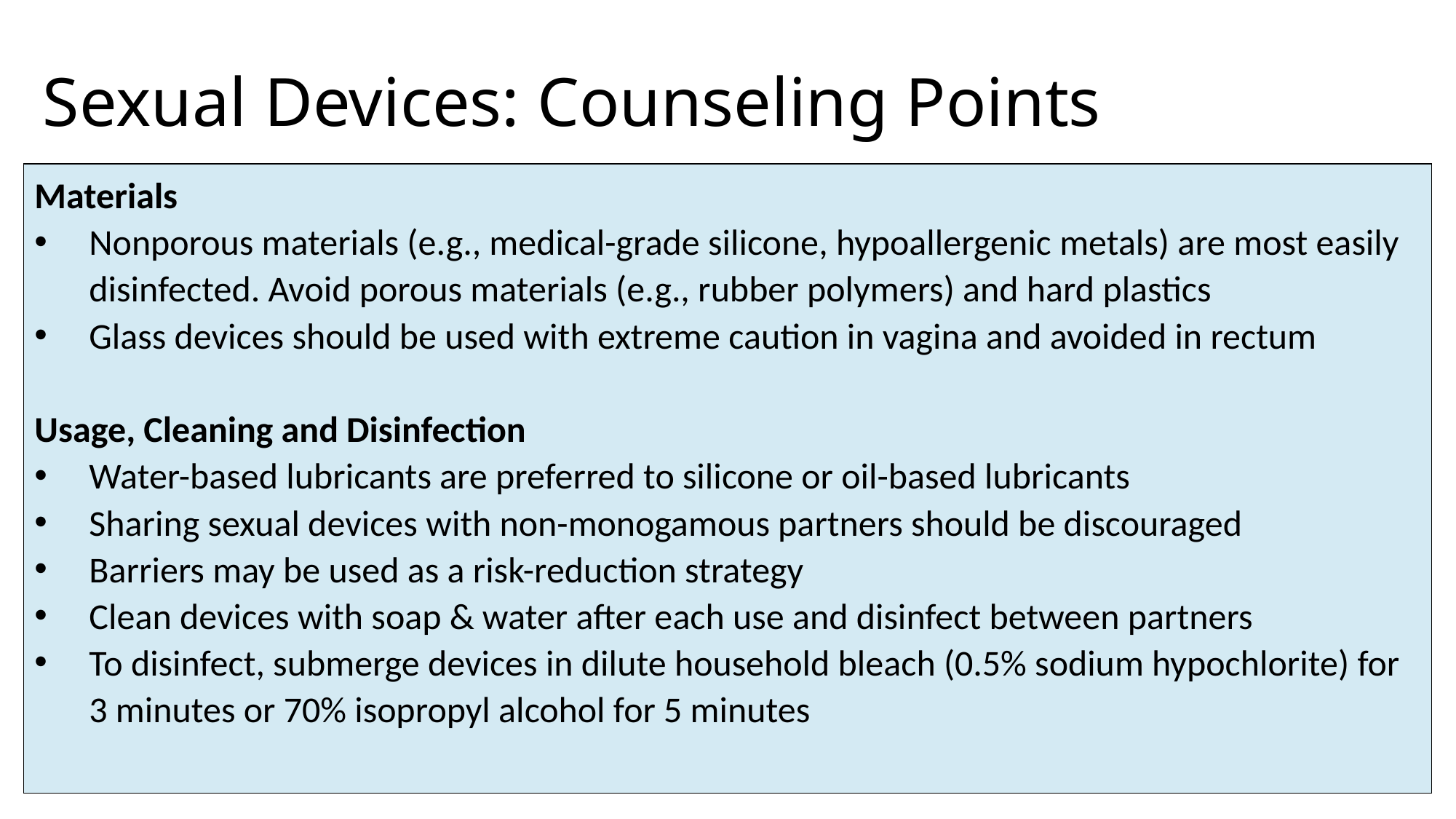

# Sexual Devices: Counseling Points
Materials
Nonporous materials (e.g., medical-grade silicone, hypoallergenic metals) are most easily disinfected. Avoid porous materials (e.g., rubber polymers) and hard plastics
Glass devices should be used with extreme caution in vagina and avoided in rectum
Usage, Cleaning and Disinfection
Water-based lubricants are preferred to silicone or oil-based lubricants
Sharing sexual devices with non-monogamous partners should be discouraged
Barriers may be used as a risk-reduction strategy
Clean devices with soap & water after each use and disinfect between partners
To disinfect, submerge devices in dilute household bleach (0.5% sodium hypochlorite) for 3 minutes or 70% isopropyl alcohol for 5 minutes

## Slide 42
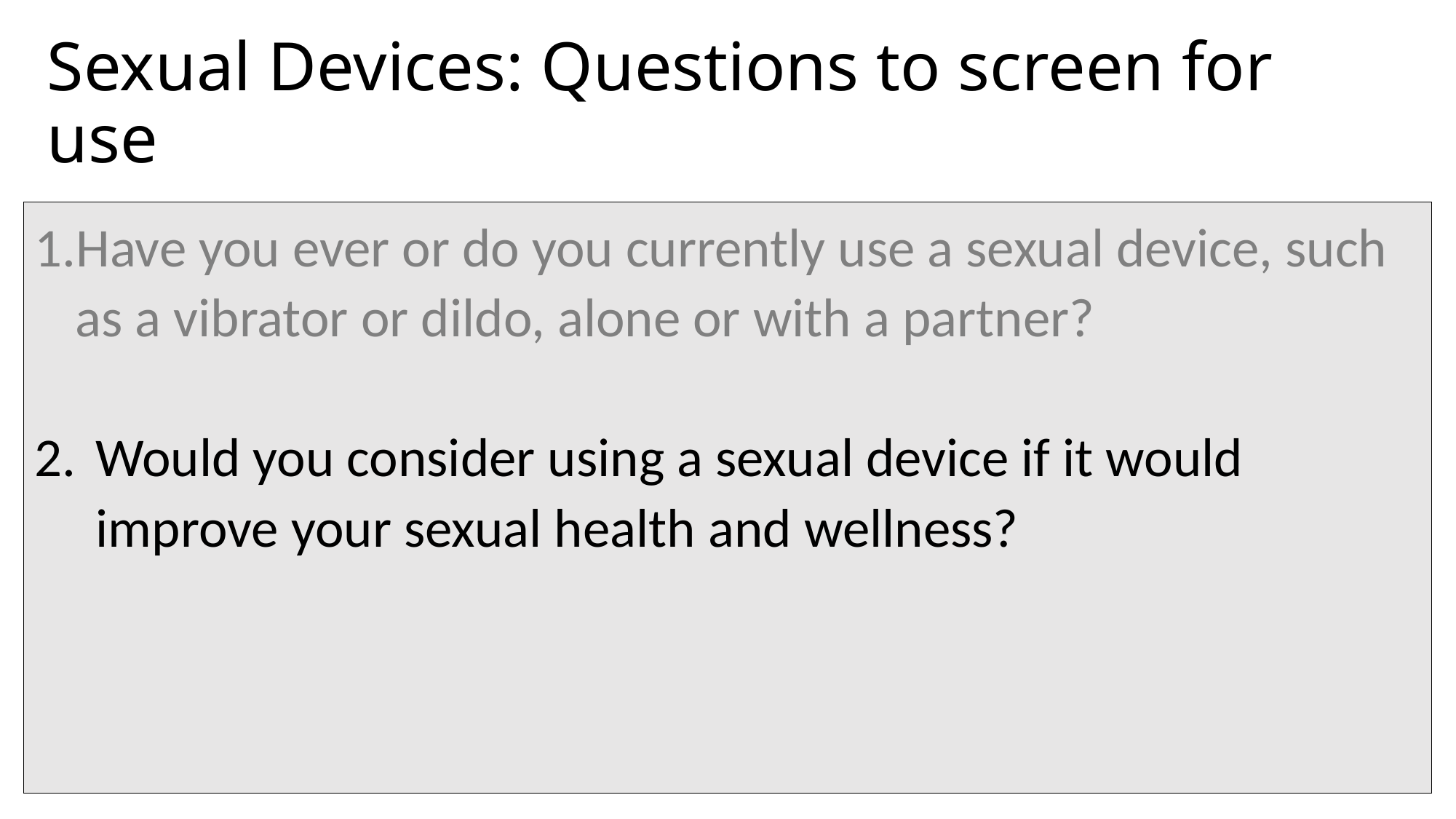

# Sexual Devices: Questions to screen for use
Have you ever or do you currently use a sexual device, such as a vibrator or dildo, alone or with a partner?
Would you consider using a sexual device if it would improve your sexual health and wellness?

## Slide 43
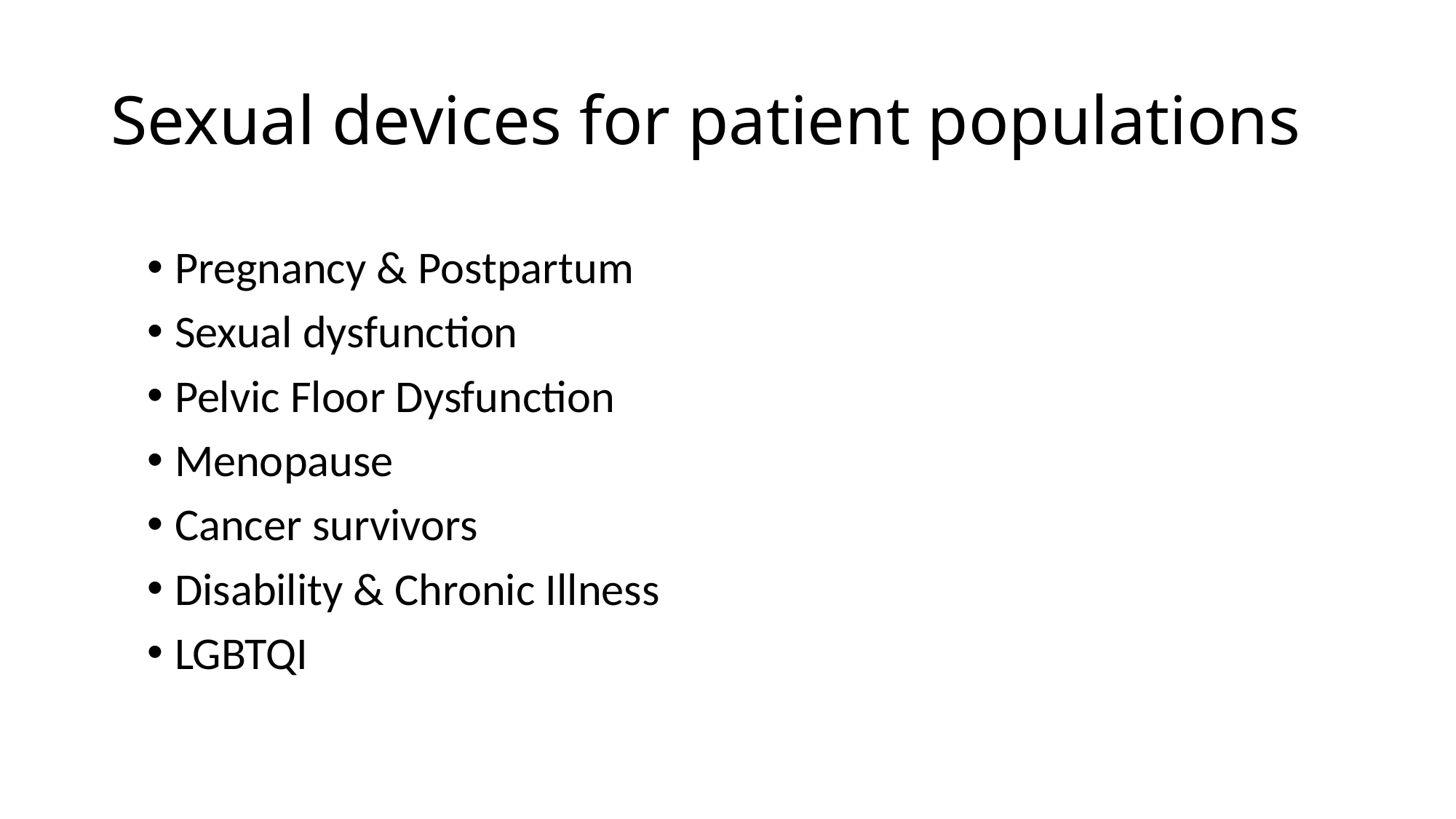

# Sexual devices for patient populations
Pregnancy & Postpartum
Sexual dysfunction
Pelvic Floor Dysfunction
Menopause
Cancer survivors
Disability & Chronic Illness
LGBTQI

## Slide 44
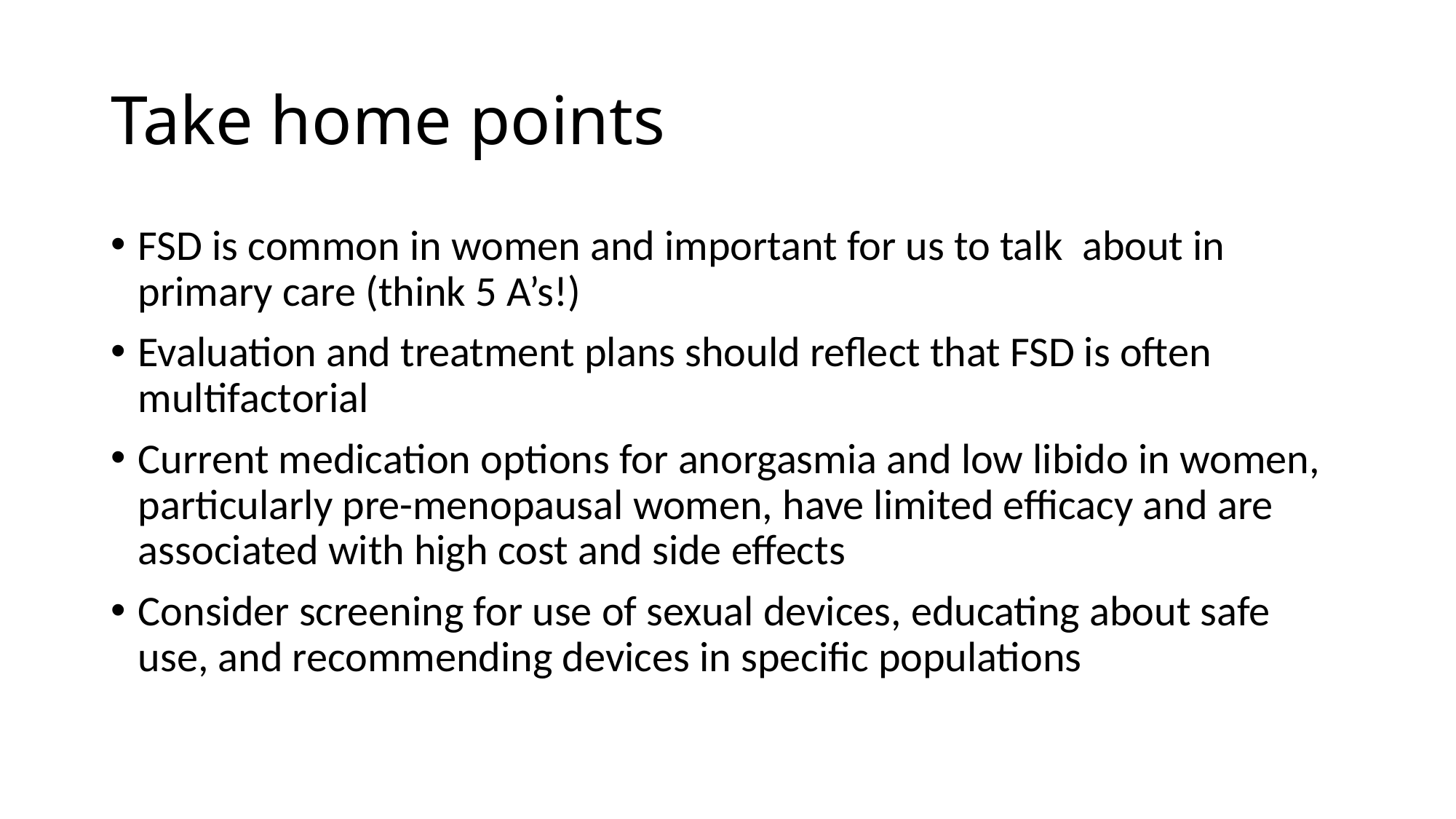

# Take home points
FSD is common in women and important for us to talk about in primary care (think 5 A’s!)
Evaluation and treatment plans should reflect that FSD is often multifactorial
Current medication options for anorgasmia and low libido in women, particularly pre-menopausal women, have limited efficacy and are associated with high cost and side effects
Consider screening for use of sexual devices, educating about safe use, and recommending devices in specific populations

## Slide 45
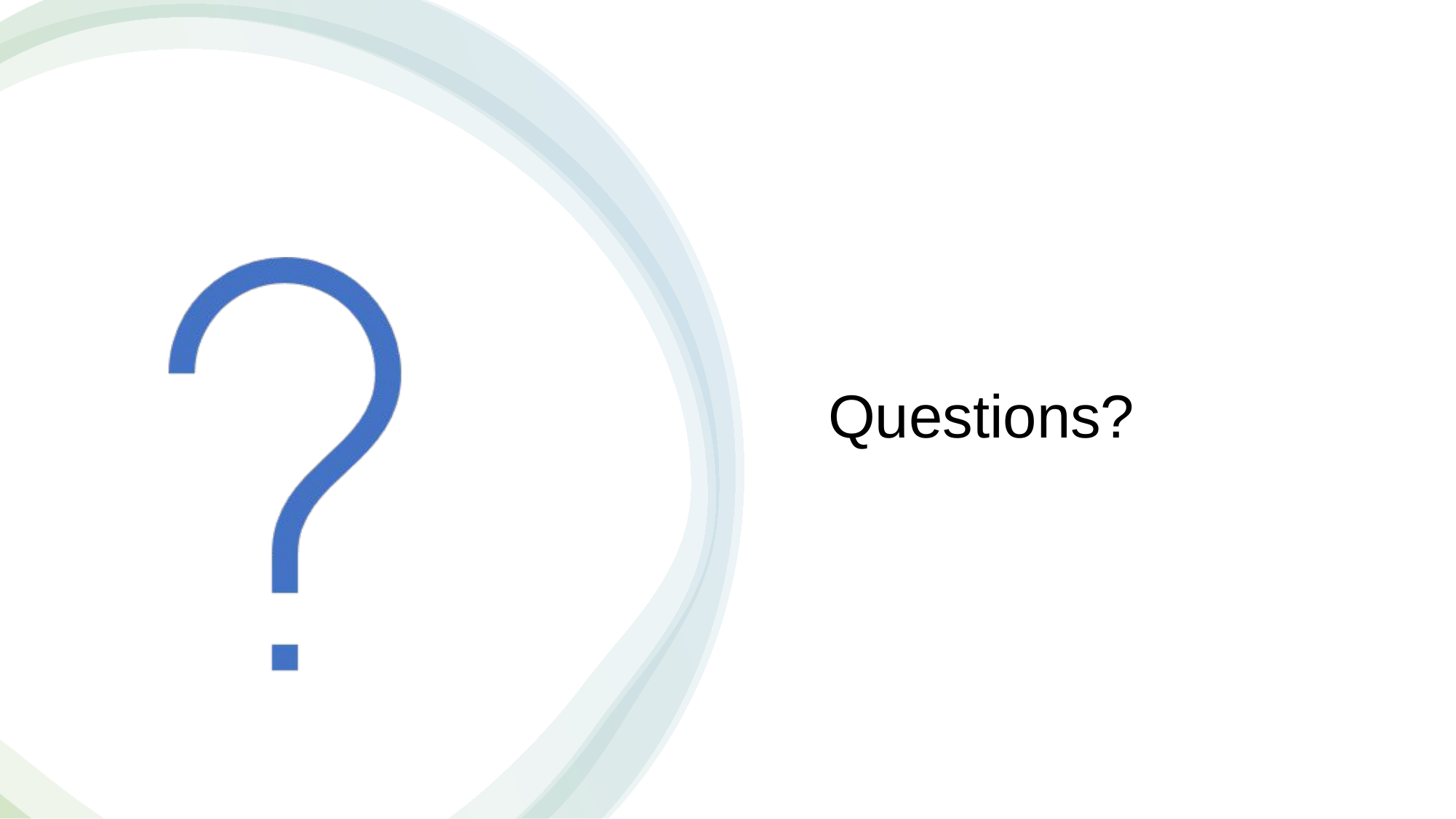

# Questions?

## Slide 46
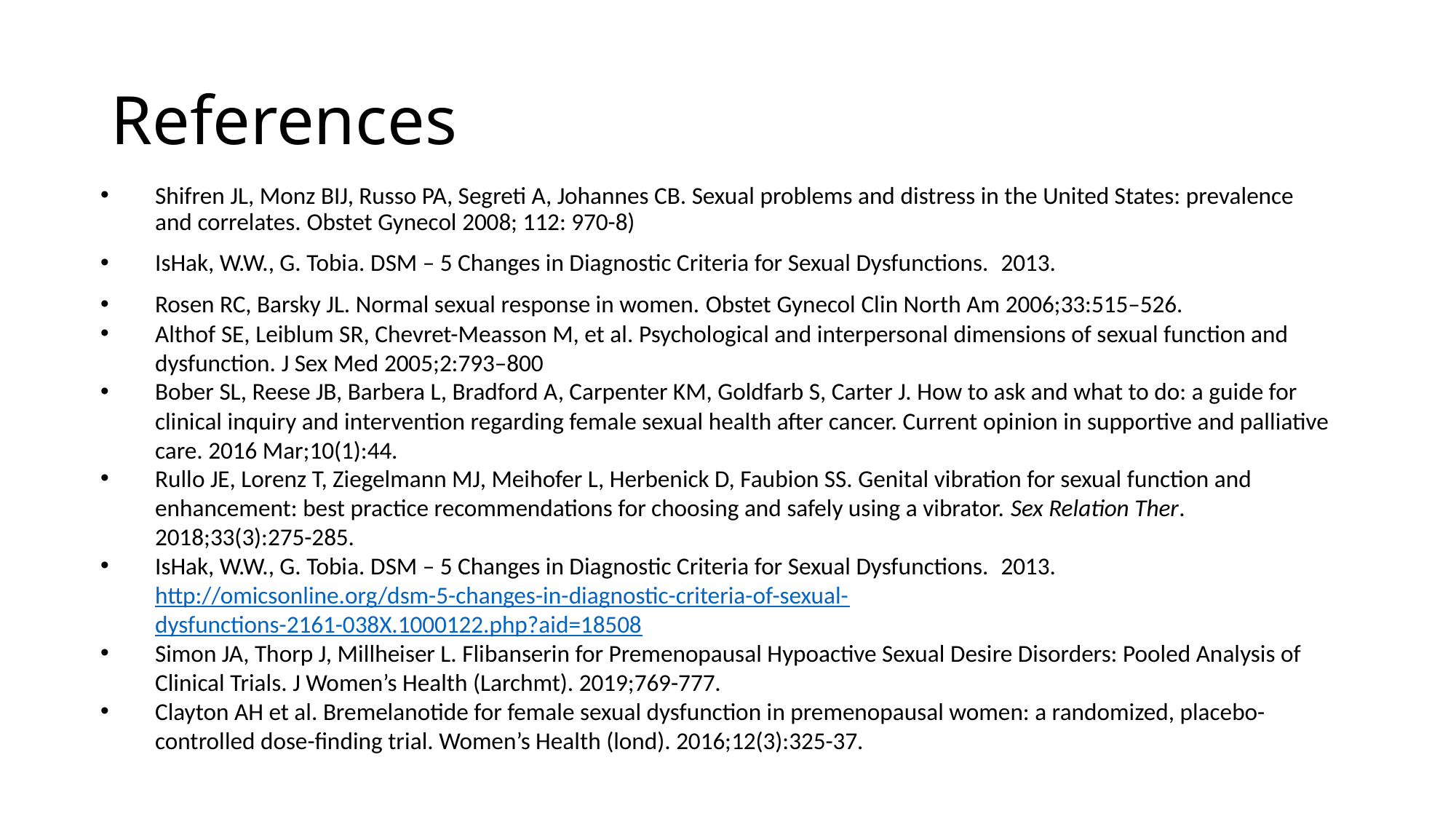

# References
Shifren JL, Monz BIJ, Russo PA, Segreti A, Johannes CB. Sexual problems and distress in the United States: prevalence and correlates. Obstet Gynecol 2008; 112: 970-8)
IsHak, W.W., G. Tobia. DSM – 5 Changes in Diagnostic Criteria for Sexual Dysfunctions.  2013.
Rosen RC, Barsky JL. Normal sexual response in women. Obstet Gynecol Clin North Am 2006;33:515–526.
Althof SE, Leiblum SR, Chevret-Measson M, et al. Psychological and interpersonal dimensions of sexual function and dysfunction. J Sex Med 2005;2:793–800
Bober SL, Reese JB, Barbera L, Bradford A, Carpenter KM, Goldfarb S, Carter J. How to ask and what to do: a guide for clinical inquiry and intervention regarding female sexual health after cancer. Current opinion in supportive and palliative care. 2016 Mar;10(1):44.
Rullo JE, Lorenz T, Ziegelmann MJ, Meihofer L, Herbenick D, Faubion SS. Genital vibration for sexual function and enhancement: best practice recommendations for choosing and safely using a vibrator. Sex Relation Ther. 2018;33(3):275-285.
IsHak, W.W., G. Tobia. DSM – 5 Changes in Diagnostic Criteria for Sexual Dysfunctions.  2013.  http://omicsonline.org/dsm-5-changes-in-diagnostic-criteria-of-sexual-dysfunctions-2161-038X.1000122.php?aid=18508
Simon JA, Thorp J, Millheiser L. Flibanserin for Premenopausal Hypoactive Sexual Desire Disorders: Pooled Analysis of Clinical Trials. J Women’s Health (Larchmt). 2019;769-777.
Clayton AH et al. Bremelanotide for female sexual dysfunction in premenopausal women: a randomized, placebo-controlled dose-finding trial. Women’s Health (lond). 2016;12(3):325-37.

## Slide 47
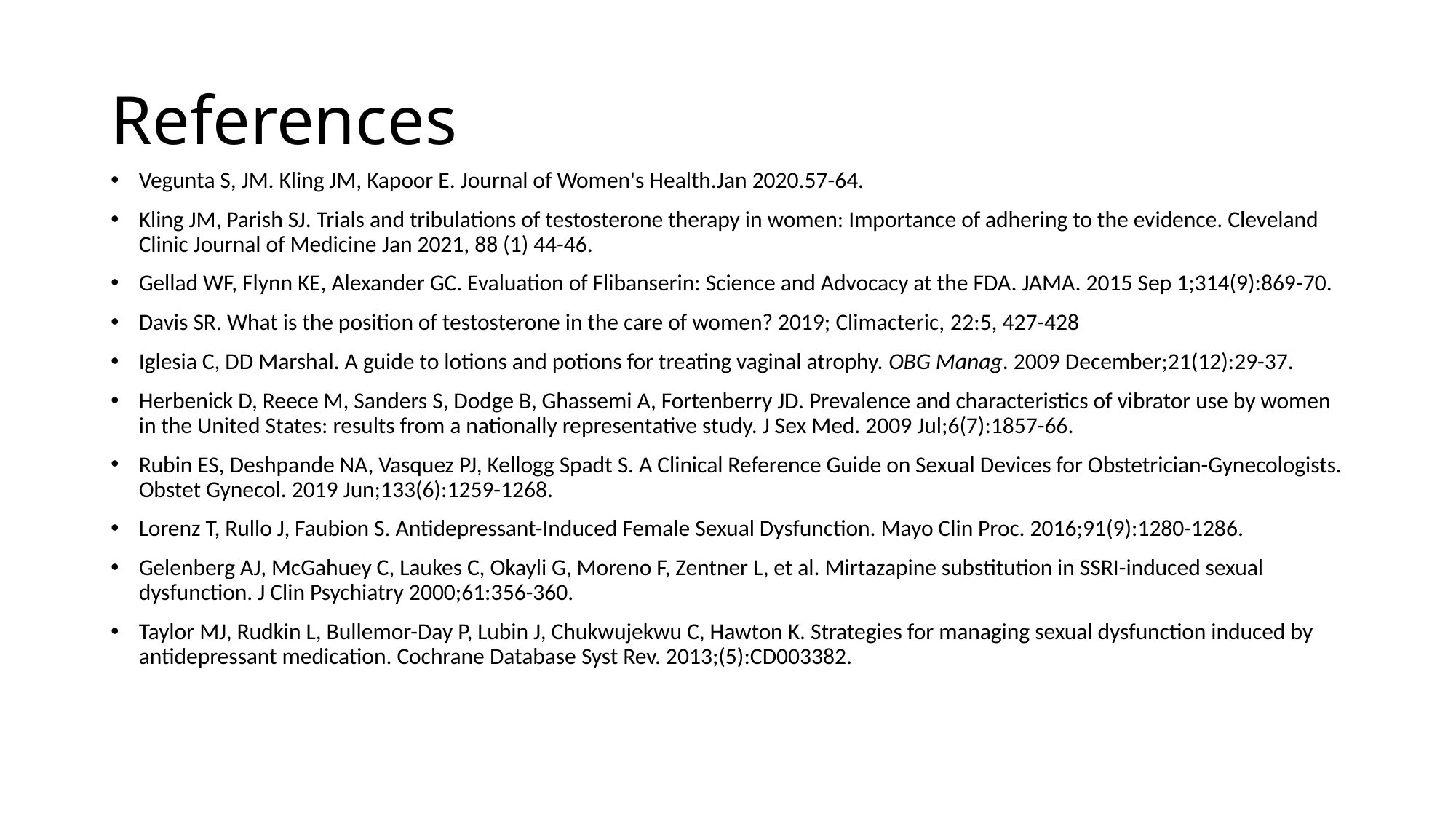

# References
Vegunta S, JM. Kling JM, Kapoor E. Journal of Women's Health.Jan 2020.57-64.
Kling JM, Parish SJ. Trials and tribulations of testosterone therapy in women: Importance of adhering to the evidence. Cleveland Clinic Journal of Medicine Jan 2021, 88 (1) 44-46.
Gellad WF, Flynn KE, Alexander GC. Evaluation of Flibanserin: Science and Advocacy at the FDA. JAMA. 2015 Sep 1;314(9):869-70.
Davis SR. What is the position of testosterone in the care of women? 2019; Climacteric, 22:5, 427-428
Iglesia C, DD Marshal. A guide to lotions and potions for treating vaginal atrophy. OBG Manag. 2009 December;21(12):29-37.
Herbenick D, Reece M, Sanders S, Dodge B, Ghassemi A, Fortenberry JD. Prevalence and characteristics of vibrator use by women in the United States: results from a nationally representative study. J Sex Med. 2009 Jul;6(7):1857-66.
Rubin ES, Deshpande NA, Vasquez PJ, Kellogg Spadt S. A Clinical Reference Guide on Sexual Devices for Obstetrician-Gynecologists. Obstet Gynecol. 2019 Jun;133(6):1259-1268.
Lorenz T, Rullo J, Faubion S. Antidepressant-Induced Female Sexual Dysfunction. Mayo Clin Proc. 2016;91(9):1280-1286.
Gelenberg AJ, McGahuey C, Laukes C, Okayli G, Moreno F, Zentner L, et al. Mirtazapine substitution in SSRI-induced sexual dysfunction. J Clin Psychiatry 2000;61:356-360.
Taylor MJ, Rudkin L, Bullemor-Day P, Lubin J, Chukwujekwu C, Hawton K. Strategies for managing sexual dysfunction induced by antidepressant medication. Cochrane Database Syst Rev. 2013;(5):CD003382.
