# Supplementary material for: Female Sexual Dysfunction: A Primer for Primary Care Health Professionals
Source: MedEdPORTAL. 2023 Apr 25;19:11312. doi: 10.15766/mep_2374-8265.11312 (PMC10126124; doi:10.15766/mep_2374-8265.11312)
Supplement: Supplementary file 1 — 60-Minute Didactic.pptx90-Minute Workshop.pptxDiscussion Cases.docxSexual Devices Language Drills.docxRole-Play Script.docxEvaluation.docx [file mep_2374-8265.11312-s001.zip › B. 90-Minute Workshop.pptx]

## Slide 1
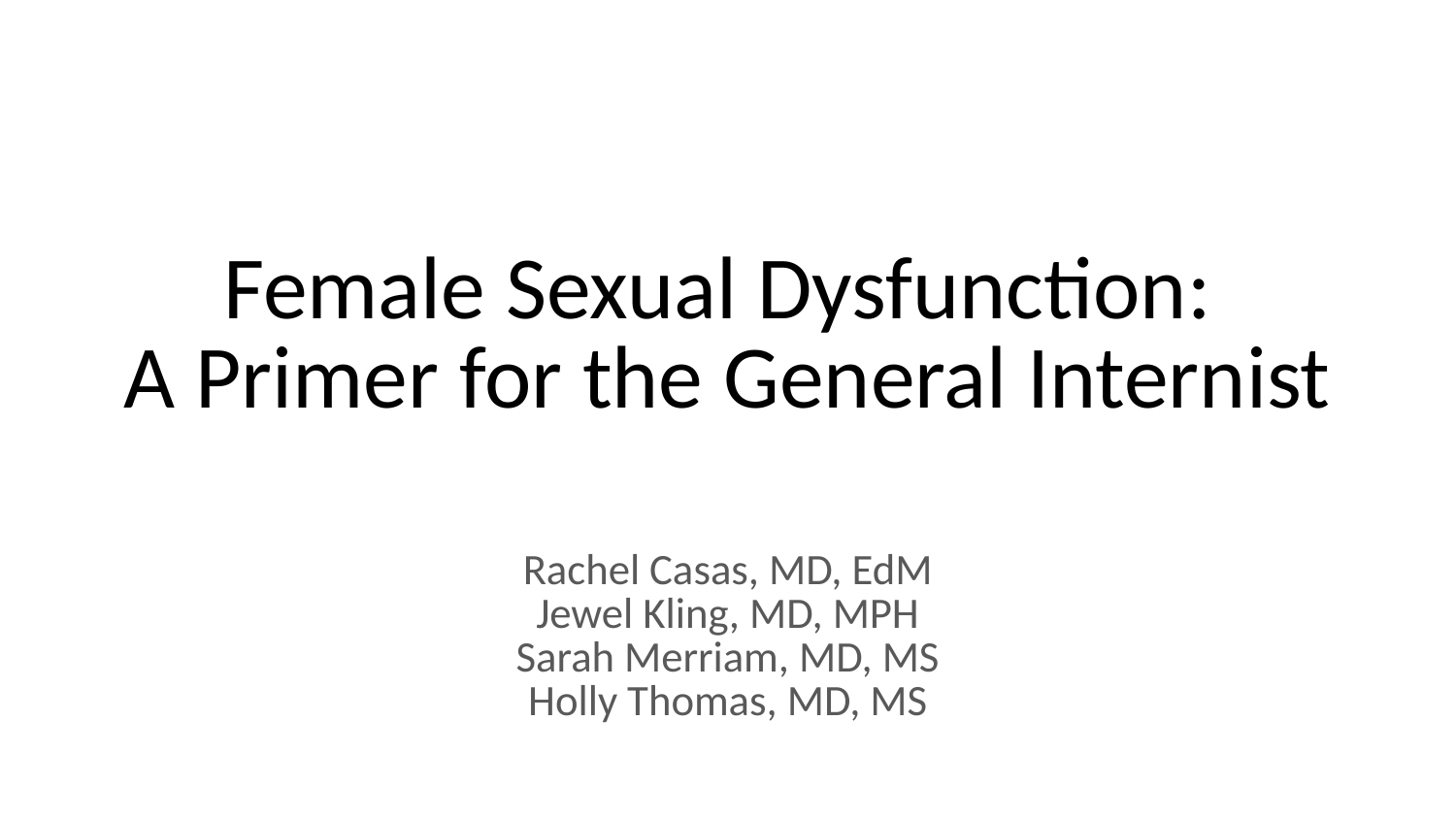

# Female Sexual Dysfunction: A Primer for the General Internist
Rachel Casas, MD, EdM
Jewel Kling, MD, MPH
Sarah Merriam, MD, MS
Holly Thomas, MD, MS

## Slide 2
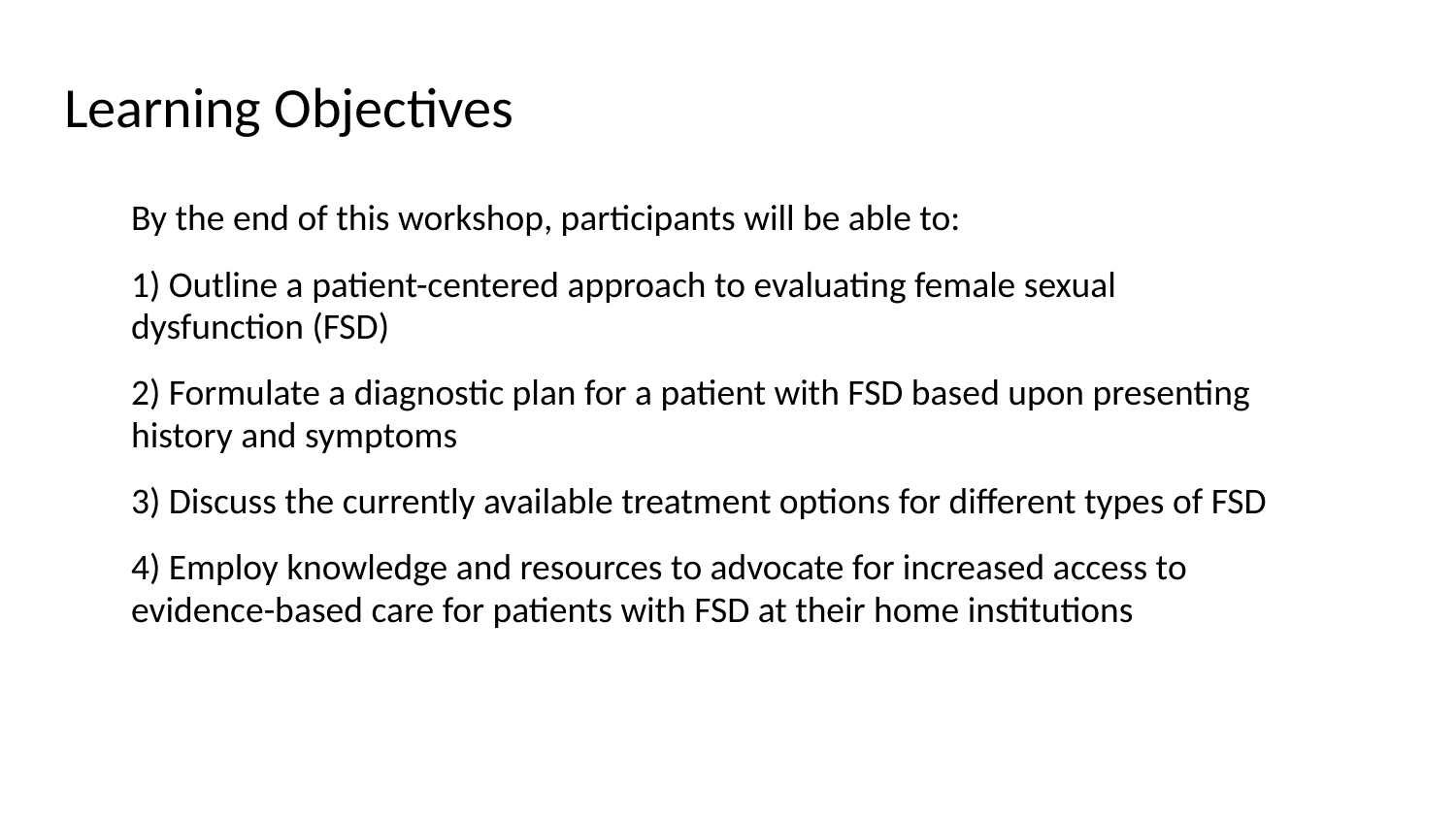

# Learning Objectives
By the end of this workshop, participants will be able to:
1) Outline a patient-centered approach to evaluating female sexual dysfunction (FSD)
2) Formulate a diagnostic plan for a patient with FSD based upon presenting history and symptoms
3) Discuss the currently available treatment options for different types of FSD
4) Employ knowledge and resources to advocate for increased access to evidence-based care for patients with FSD at their home institutions

## Slide 3
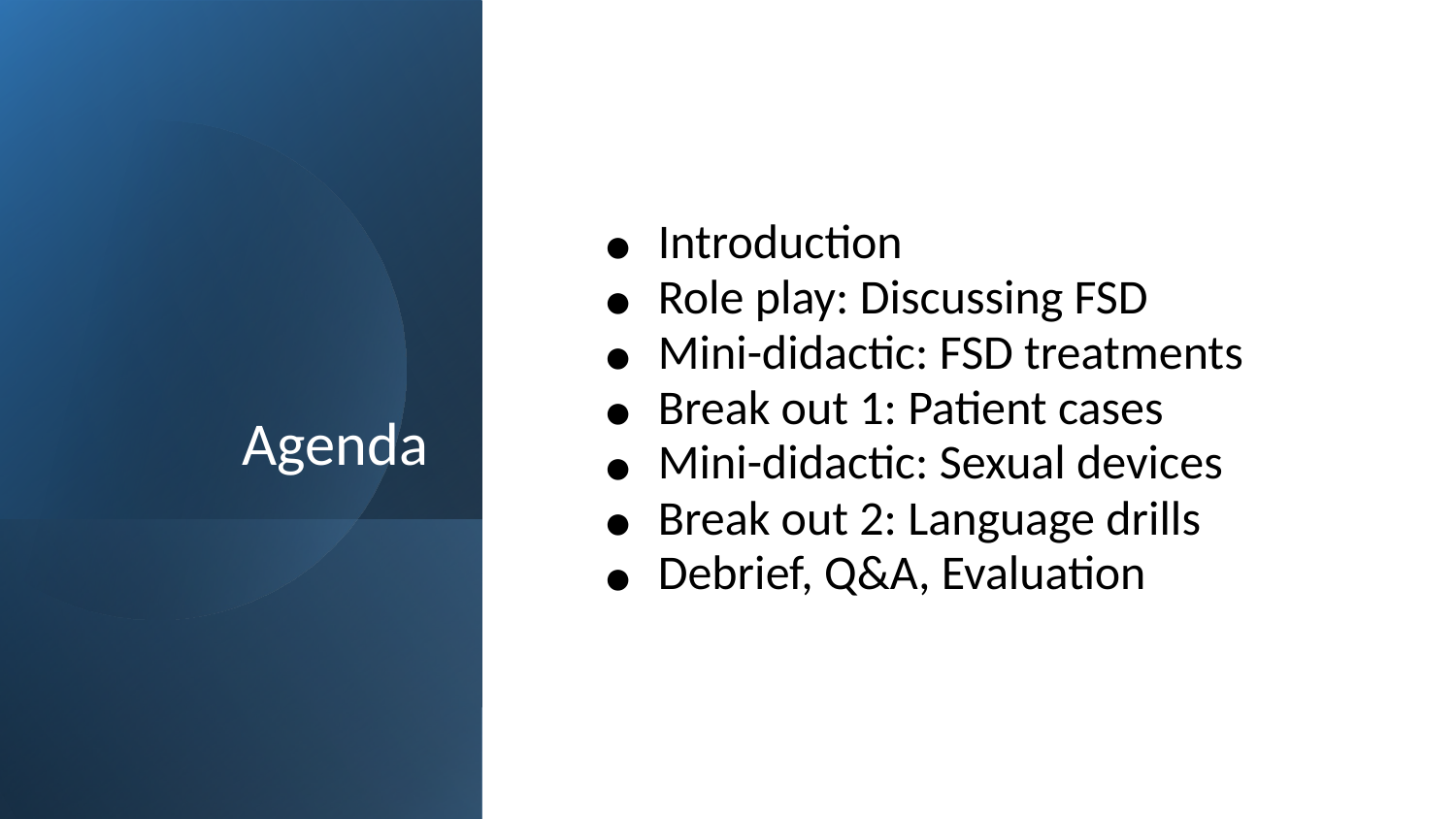

# Agenda
Introduction
Role play: Discussing FSD
Mini-didactic: FSD treatments
Break out 1: Patient cases
Mini-didactic: Sexual devices
Break out 2: Language drills
Debrief, Q&A, Evaluation

## Slide 4
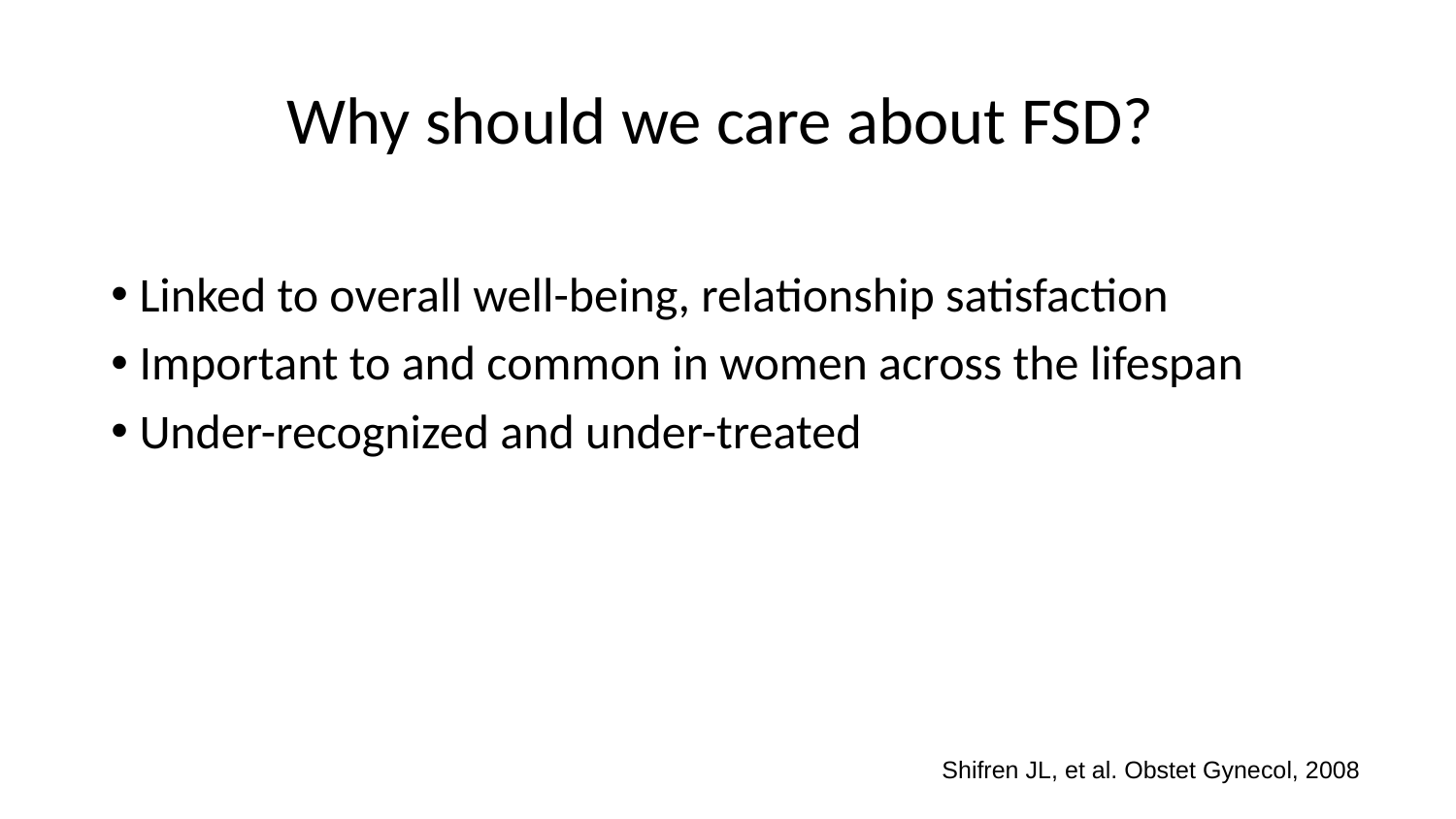

# Why should we care about FSD?
Linked to overall well-being, relationship satisfaction
Important to and common in women across the lifespan
Under-recognized and under-treated
Shifren JL, et al. Obstet Gynecol, 2008

## Slide 5
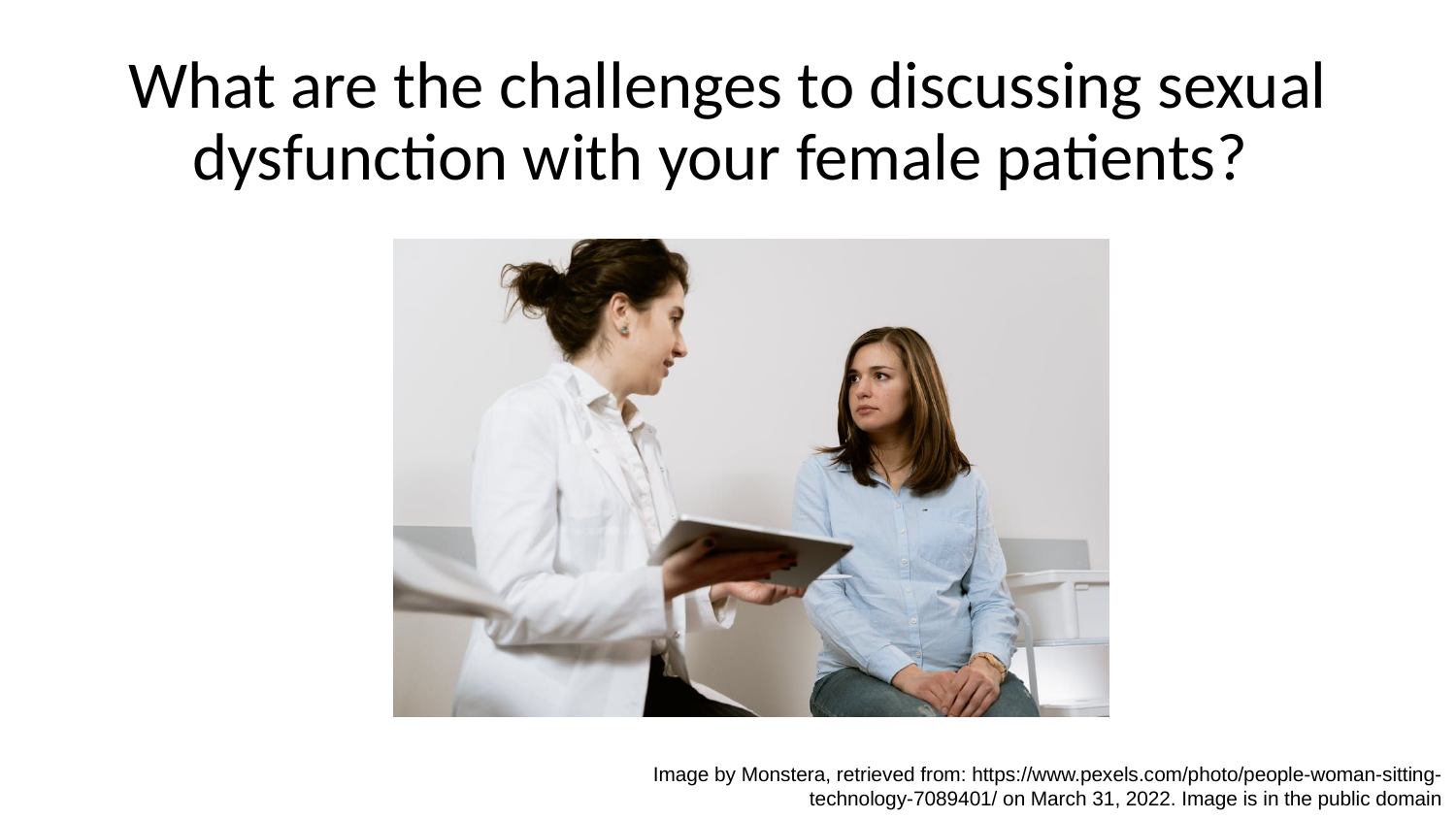

# What are the challenges to discussing sexual dysfunction with your female patients?
Image by Monstera, retrieved from: https://www.pexels.com/photo/people-woman-sitting-technology-7089401/ on March 31, 2022. Image is in the public domain

## Slide 6
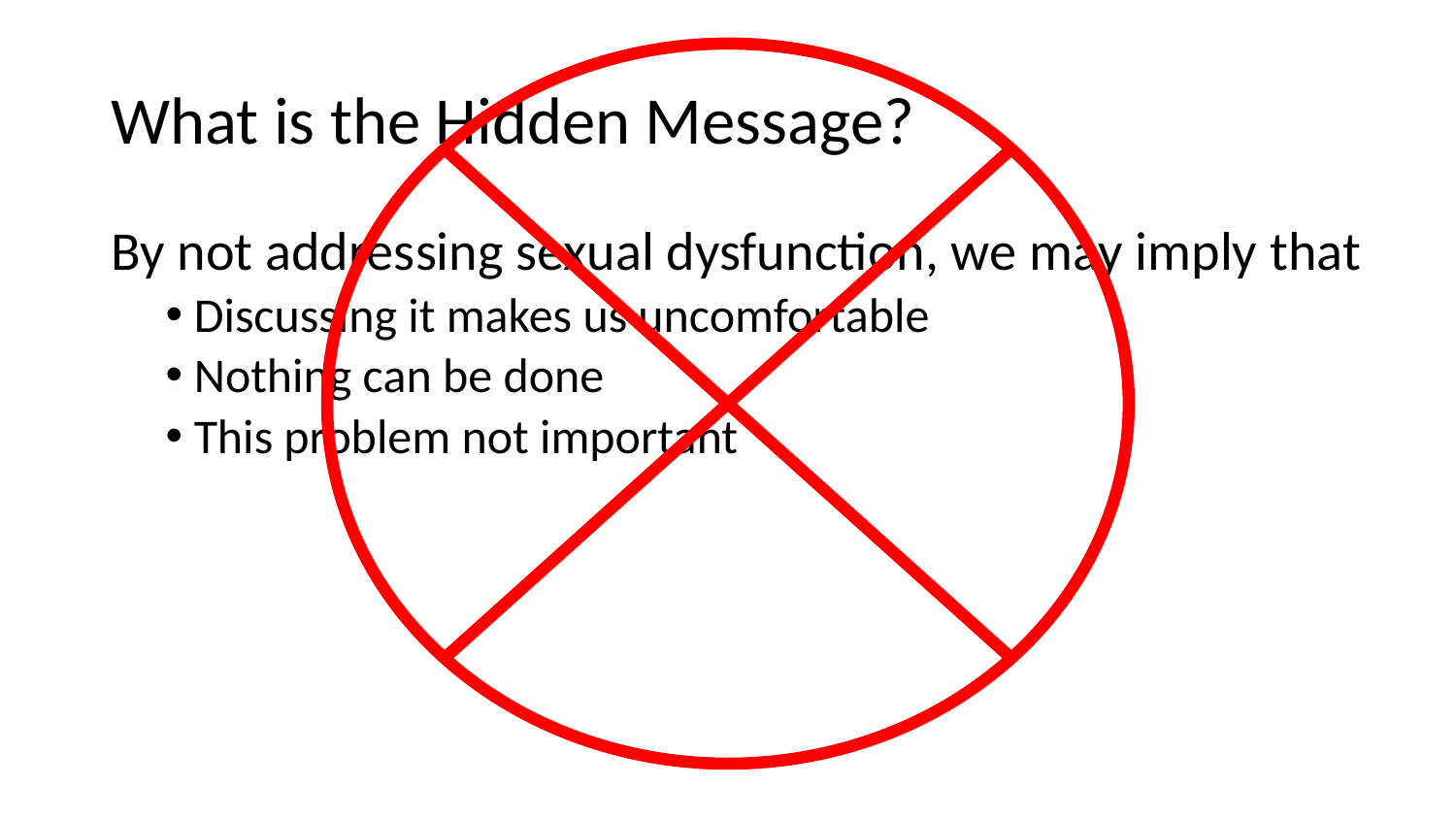

# What is the Hidden Message?
By not addressing sexual dysfunction, we may imply that
Discussing it makes us uncomfortable
Nothing can be done
This problem not important

## Slide 7
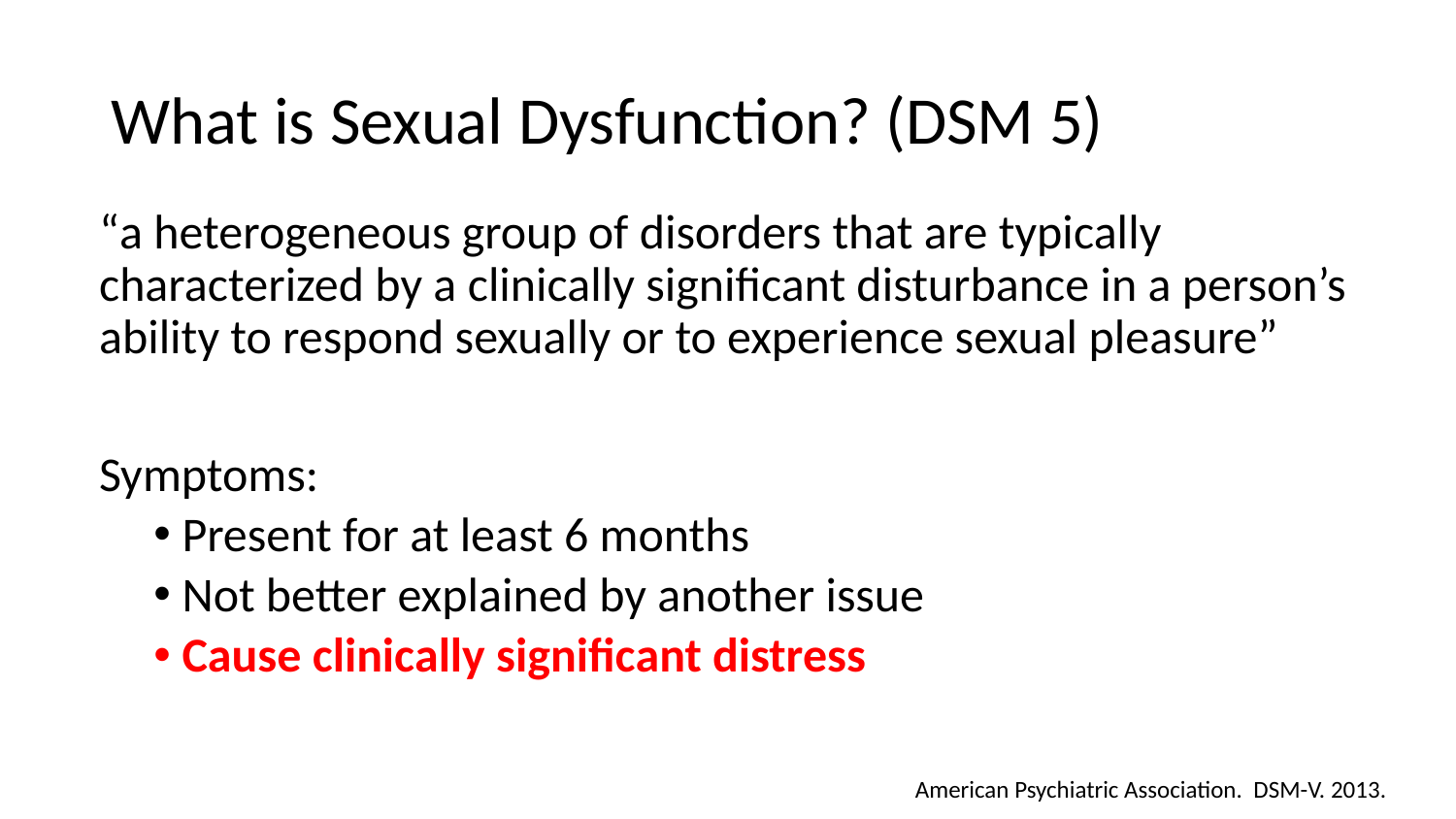

# What is Sexual Dysfunction? (DSM 5)
“a heterogeneous group of disorders that are typically characterized by a clinically significant disturbance in a person’s ability to respond sexually or to experience sexual pleasure”
Symptoms:
Present for at least 6 months
Not better explained by another issue
Cause clinically significant distress
American Psychiatric Association. DSM-V. 2013.

## Slide 8
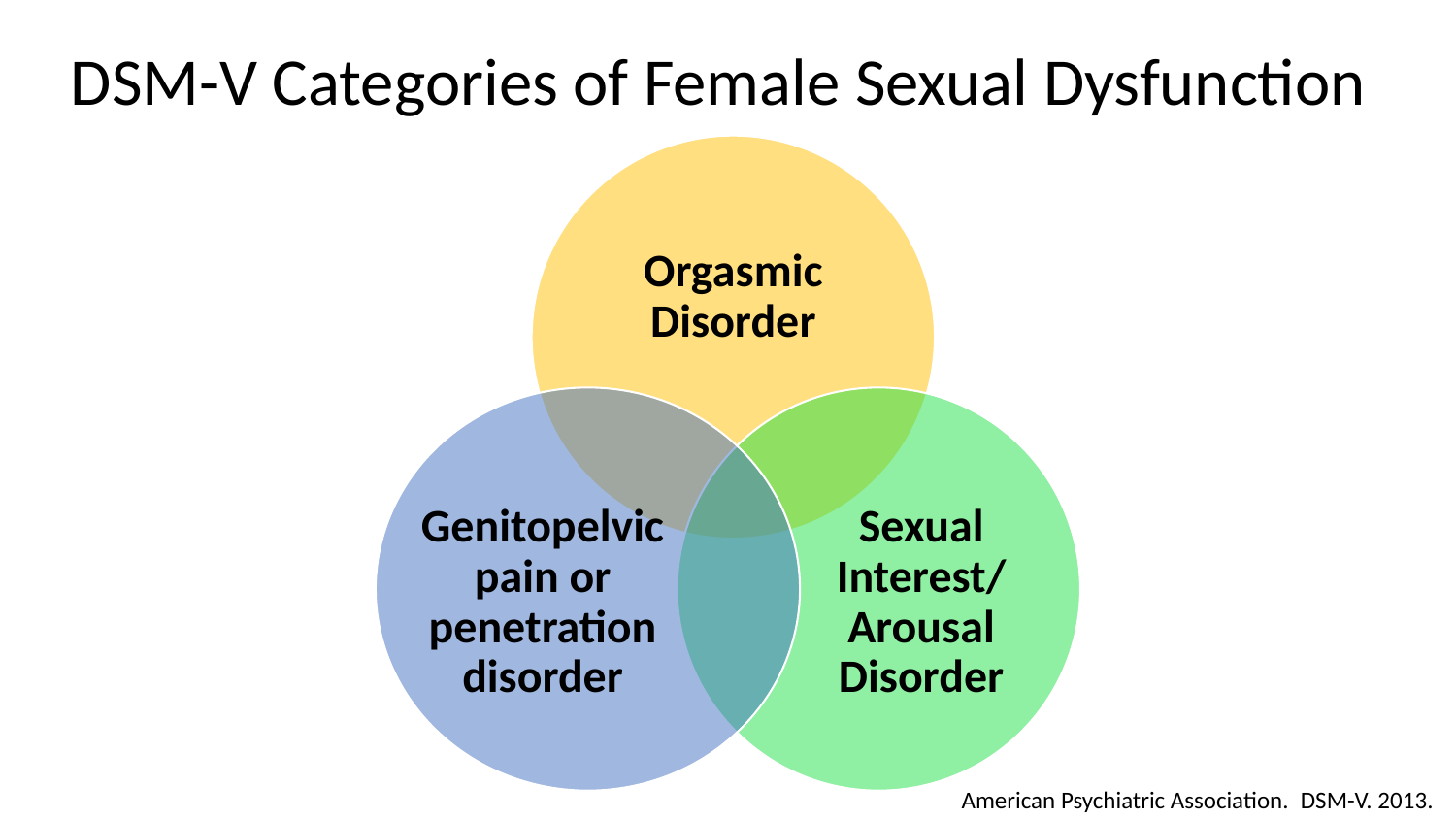

# DSM-V Categories of Female Sexual Dysfunction
Orgasmic Disorder
Genitopelvic pain or penetration disorder
Sexual Interest/ Arousal Disorder
American Psychiatric Association.  DSM-V. 2013.

## Slide 9
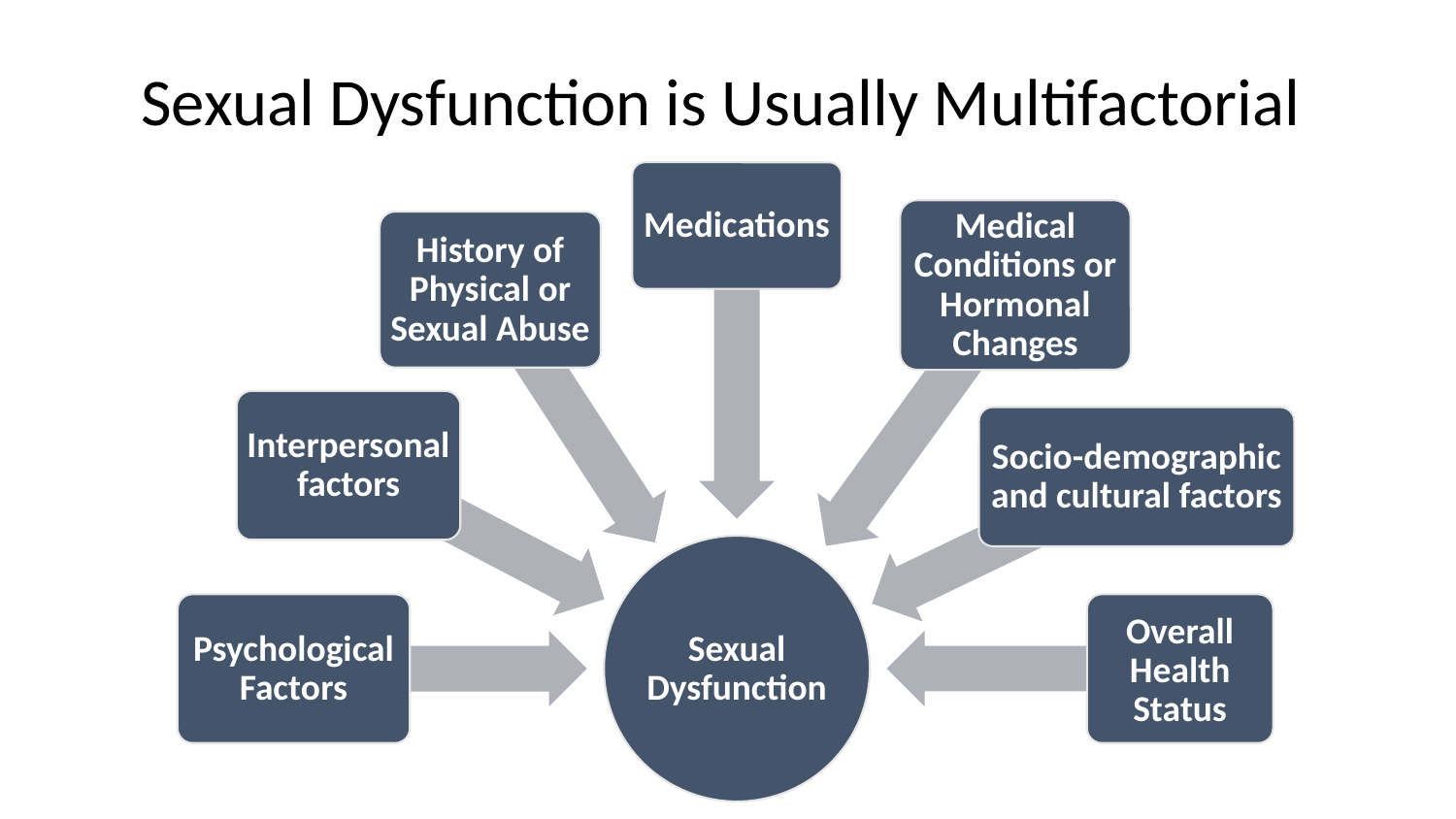

# Sexual Dysfunction is Usually Multifactorial
Medications
Medical Conditions or Hormonal Changes
History of Physical or Sexual Abuse
Interpersonal factors
Socio-demographic and cultural factors
Sexual Dysfunction
Psychological Factors
Overall Health Status

## Slide 10
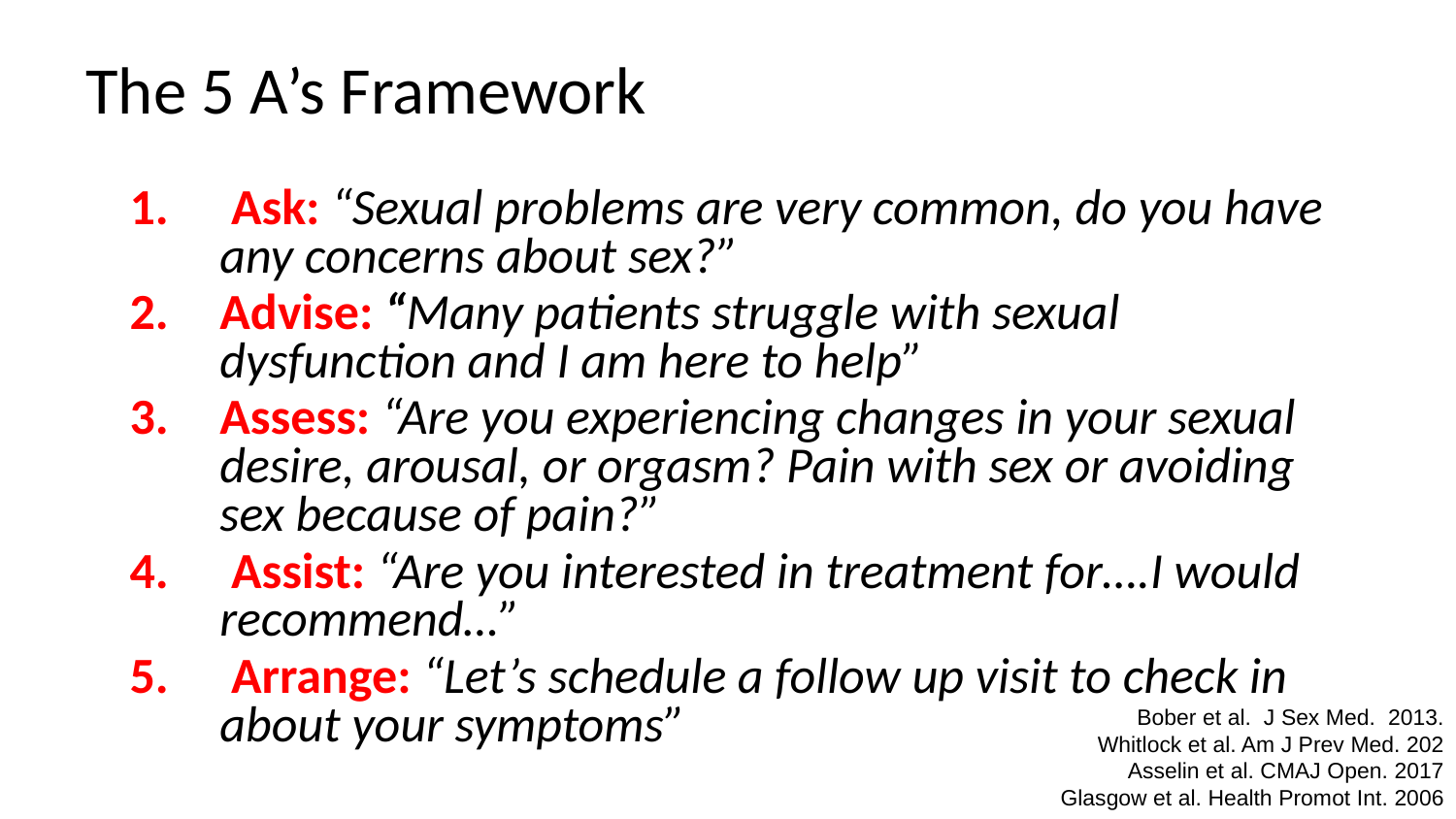

# The 5 A’s Framework
 Ask: “Sexual problems are very common, do you have any concerns about sex?”
Advise: “Many patients struggle with sexual dysfunction and I am here to help”
Assess: “Are you experiencing changes in your sexual desire, arousal, or orgasm? Pain with sex or avoiding sex because of pain?”
 Assist: “Are you interested in treatment for….I would recommend…”
 Arrange: “Let’s schedule a follow up visit to check in about your symptoms”
Bober et al. J Sex Med. 2013.
Whitlock et al. Am J Prev Med. 202
Asselin et al. CMAJ Open. 2017
Glasgow et al. Health Promot Int. 2006

## Slide 11
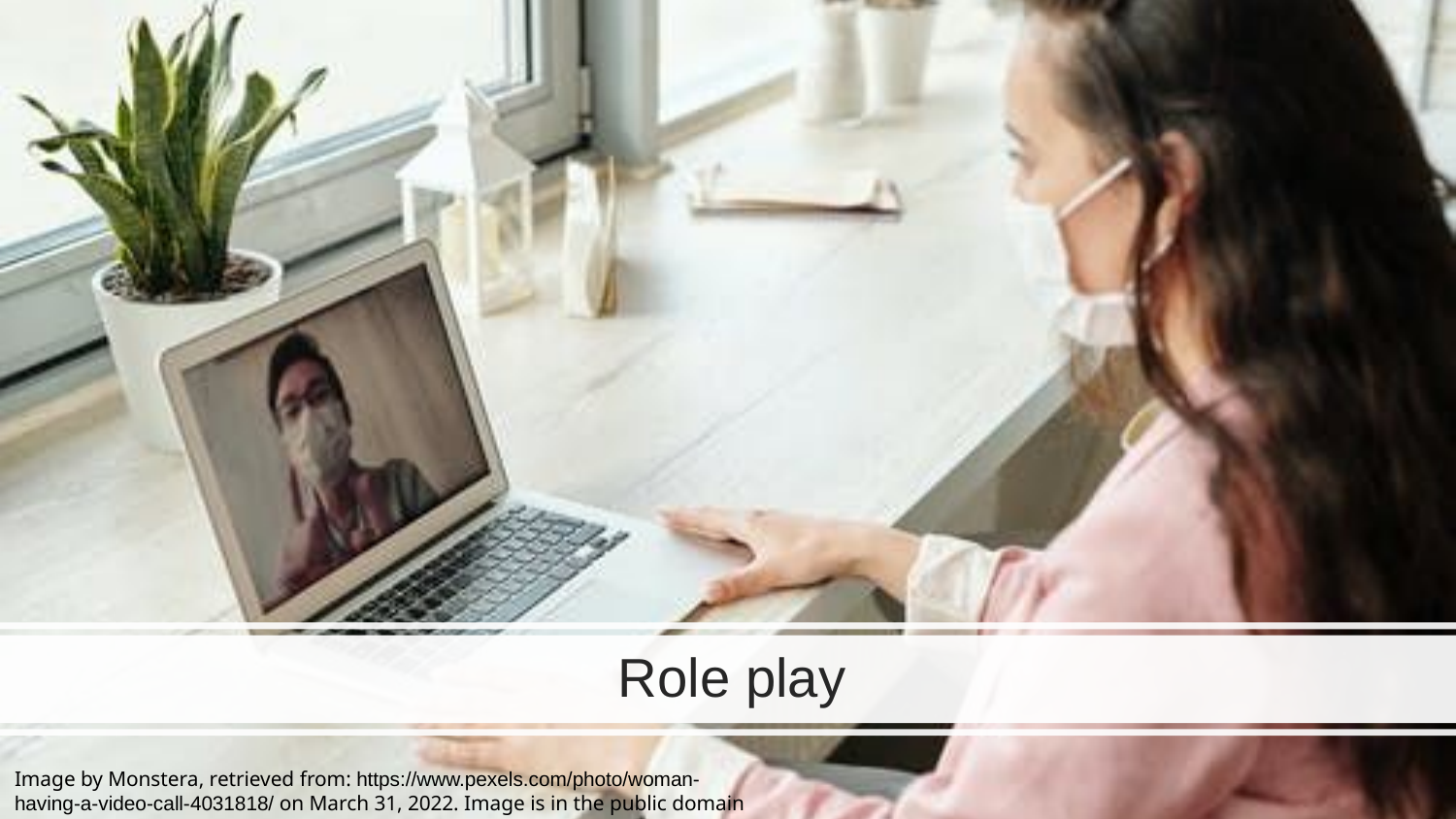

# Role play
Image by Monstera, retrieved from: https://www.pexels.com/photo/woman-having-a-video-call-4031818/ on March 31, 2022. Image is in the public domain

## Slide 12
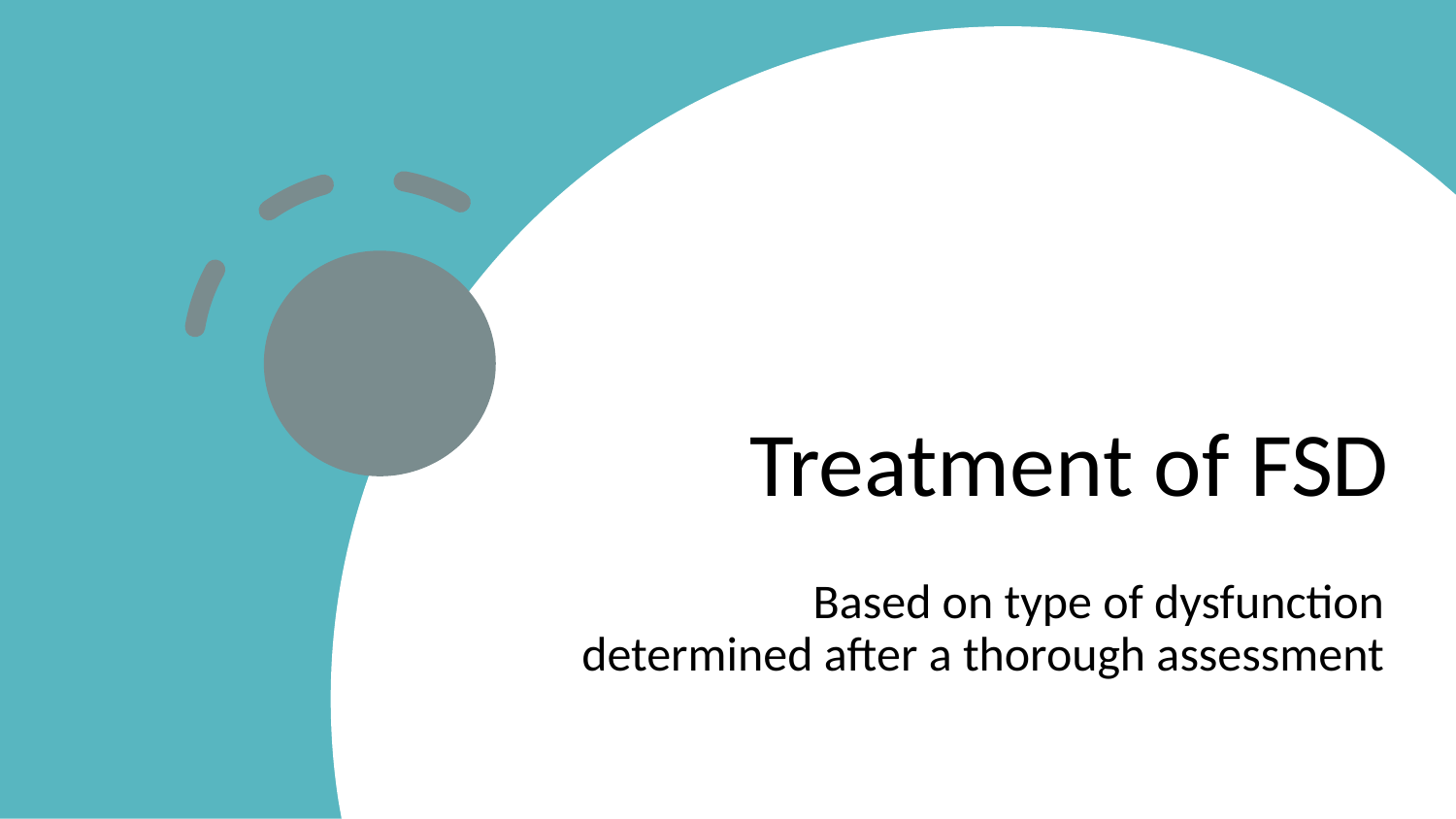

# Treatment of FSD
Based on type of dysfunction determined after a thorough assessment

## Slide 13
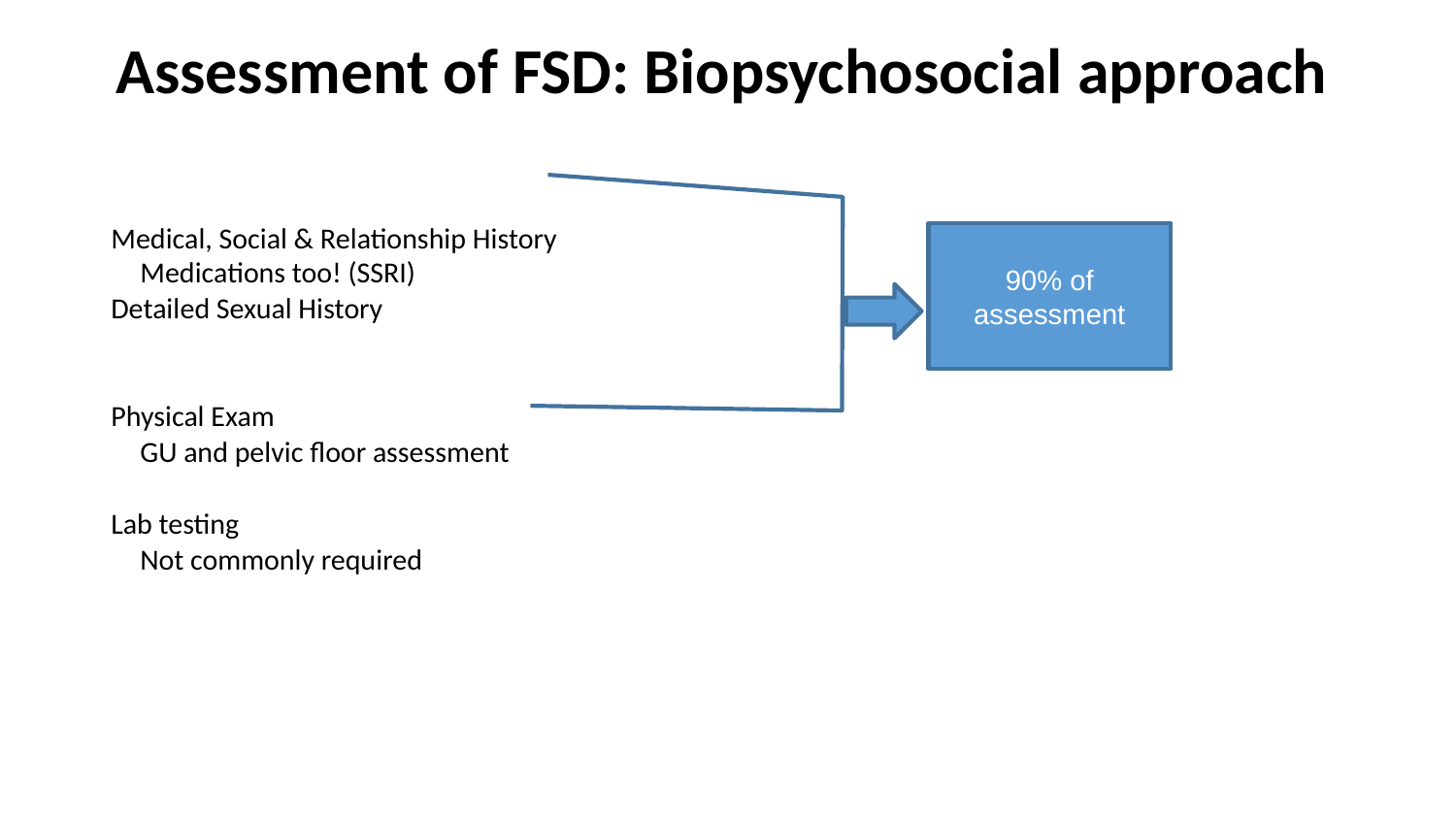

# Assessment of FSD: Biopsychosocial approach
Medical, Social & Relationship History
	Medications too! (SSRI)
Detailed Sexual History
Physical Exam
	GU and pelvic floor assessment
Lab testing
	Not commonly required
90% of assessment

## Slide 14
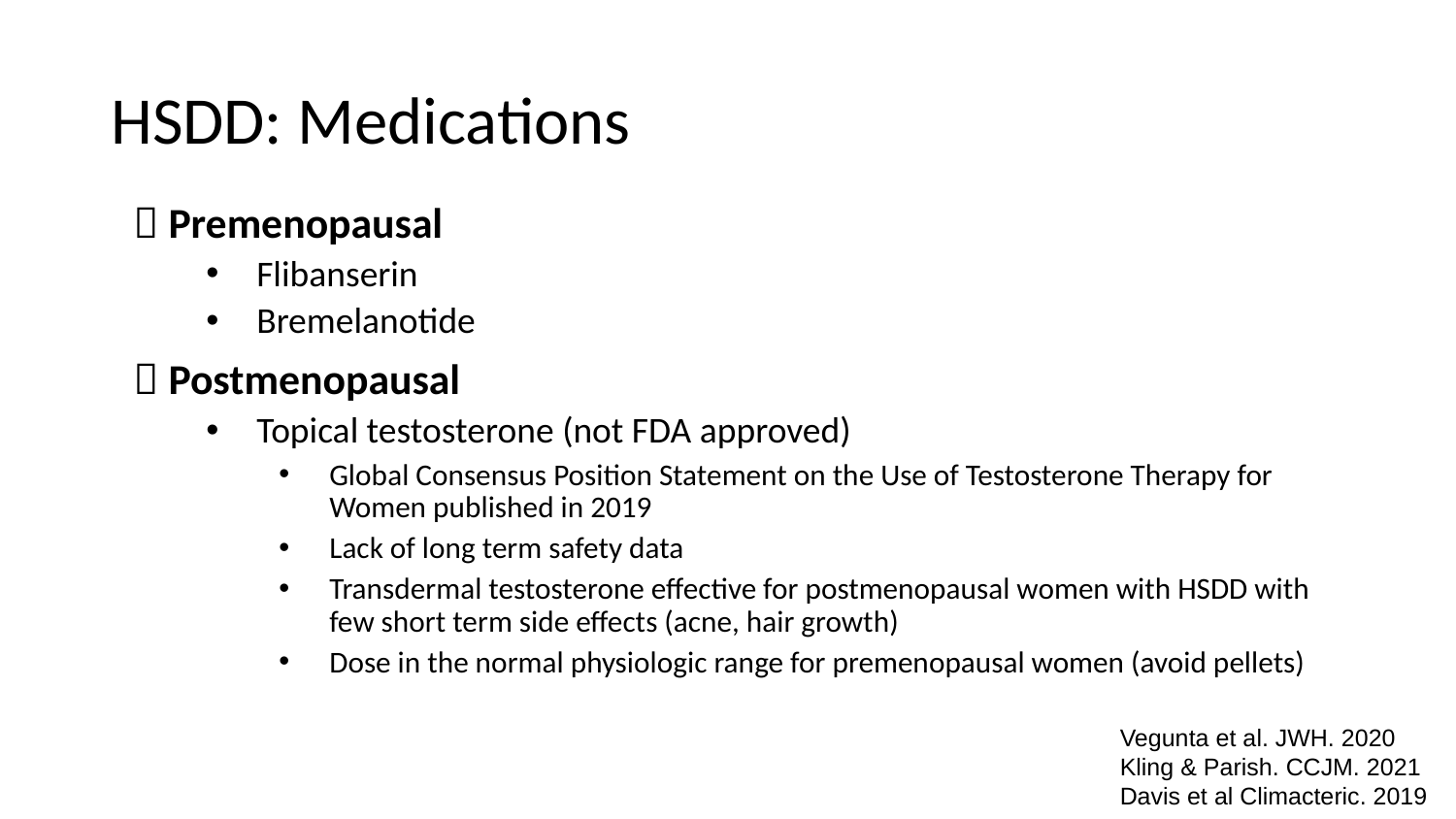

# HSDD: Medications
 Premenopausal
Flibanserin
Bremelanotide
 Postmenopausal
Topical testosterone (not FDA approved)
Global Consensus Position Statement on the Use of Testosterone Therapy for Women published in 2019
Lack of long term safety data
Transdermal testosterone effective for postmenopausal women with HSDD with few short term side effects (acne, hair growth)
Dose in the normal physiologic range for premenopausal women (avoid pellets)
Vegunta et al. JWH. 2020
Kling & Parish. CCJM. 2021
Davis et al Climacteric. 2019

## Slide 15
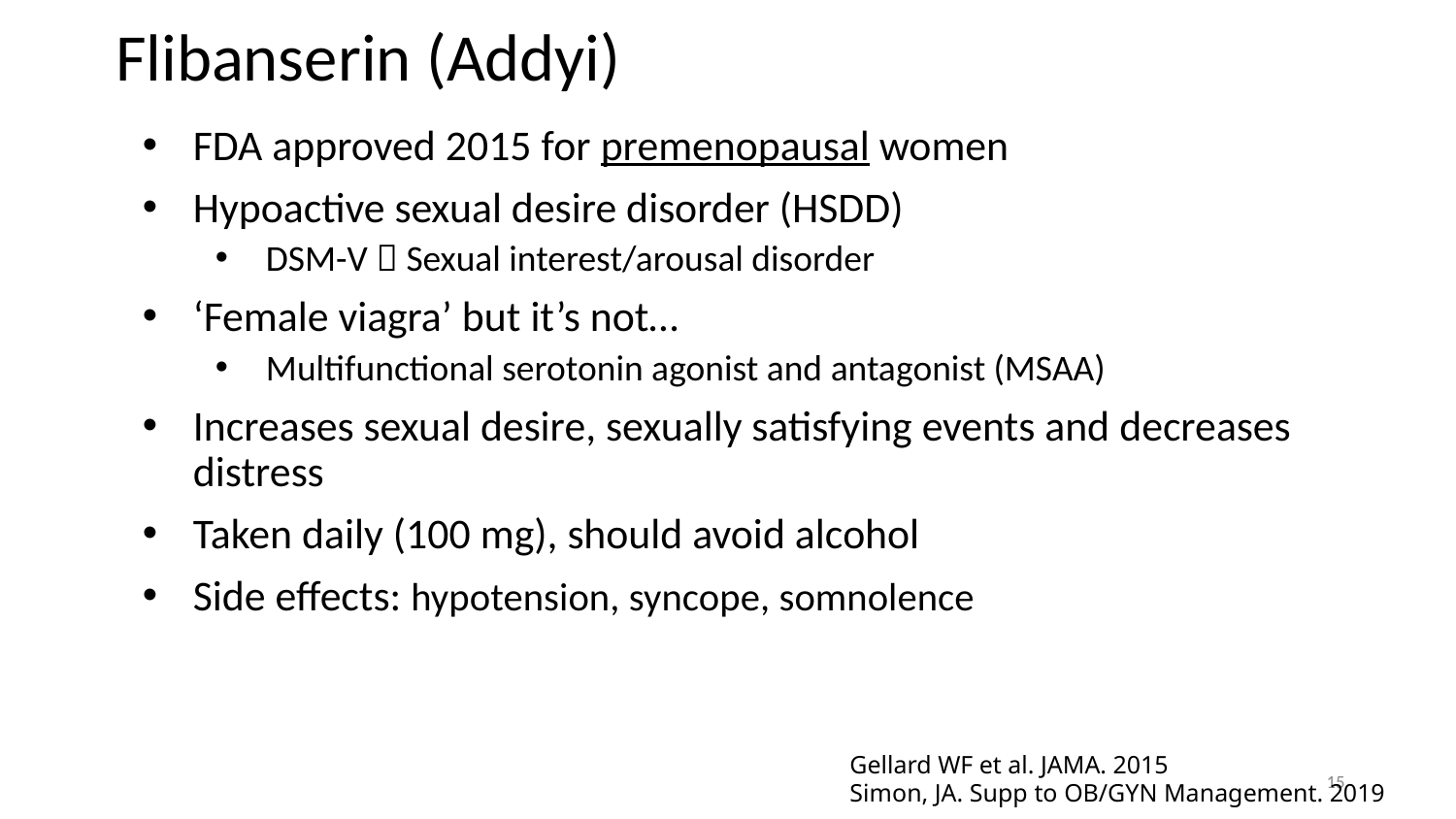

# Flibanserin (Addyi)
FDA approved 2015 for premenopausal women
Hypoactive sexual desire disorder (HSDD)
DSM-V  Sexual interest/arousal disorder
‘Female viagra’ but it’s not…
Multifunctional serotonin agonist and antagonist (MSAA)
Increases sexual desire, sexually satisfying events and decreases distress
Taken daily (100 mg), should avoid alcohol
Side effects: hypotension, syncope, somnolence
Gellard WF et al. JAMA. 2015
Simon, JA. Supp to OB/GYN Management. 2019
15

## Slide 16
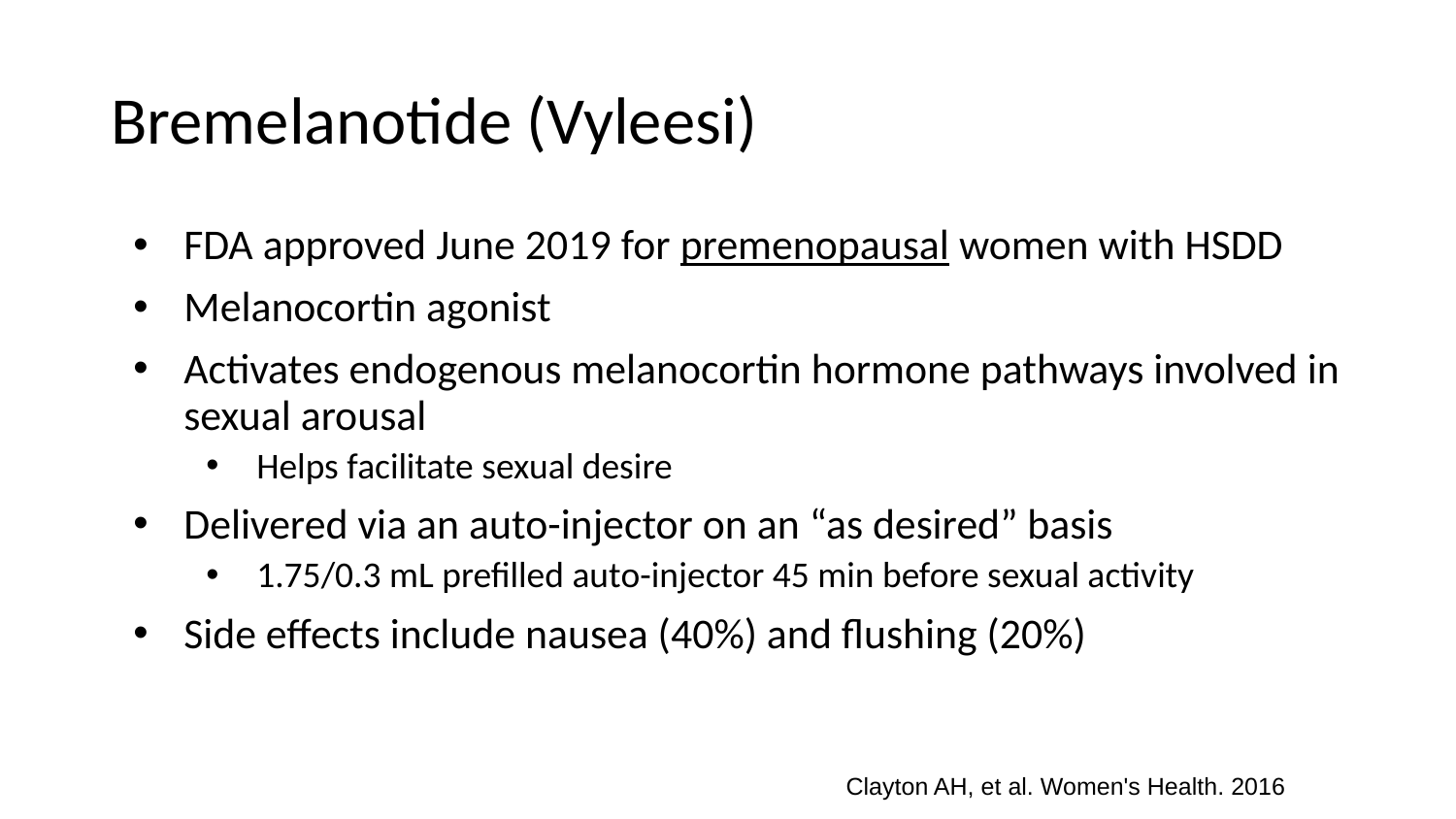

# Bremelanotide (Vyleesi)
FDA approved June 2019 for premenopausal women with HSDD
Melanocortin agonist
Activates endogenous melanocortin hormone pathways involved in sexual arousal
Helps facilitate sexual desire
Delivered via an auto-injector on an “as desired” basis
1.75/0.3 mL prefilled auto-injector 45 min before sexual activity
Side effects include nausea (40%) and flushing (20%)
Clayton AH, et al. Women's Health. 2016

## Slide 17
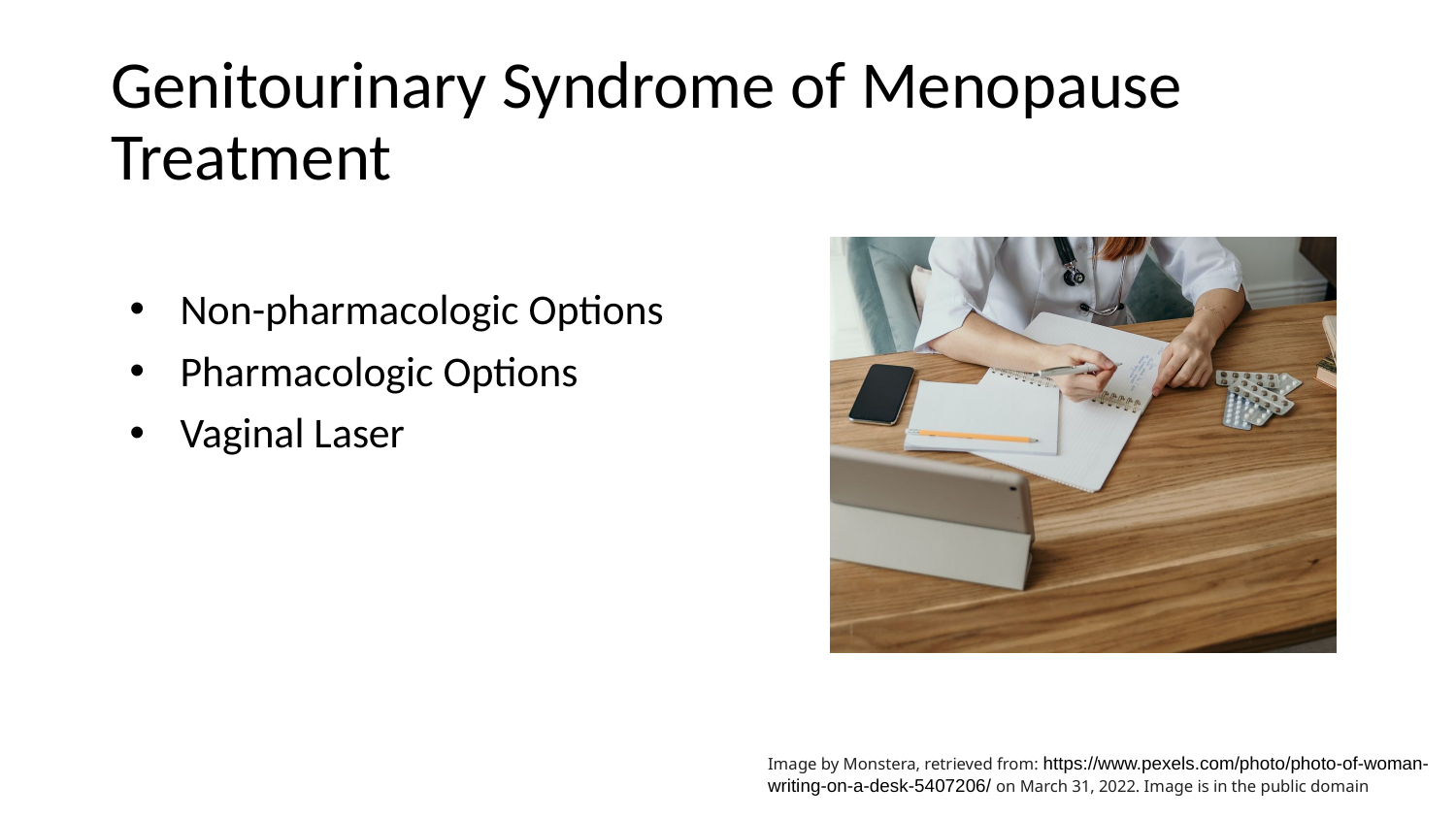

# Genitourinary Syndrome of Menopause Treatment
Non-pharmacologic Options
Pharmacologic Options
Vaginal Laser
Image by Monstera, retrieved from: https://www.pexels.com/photo/photo-of-woman-writing-on-a-desk-5407206/ on March 31, 2022. Image is in the public domain

## Slide 18
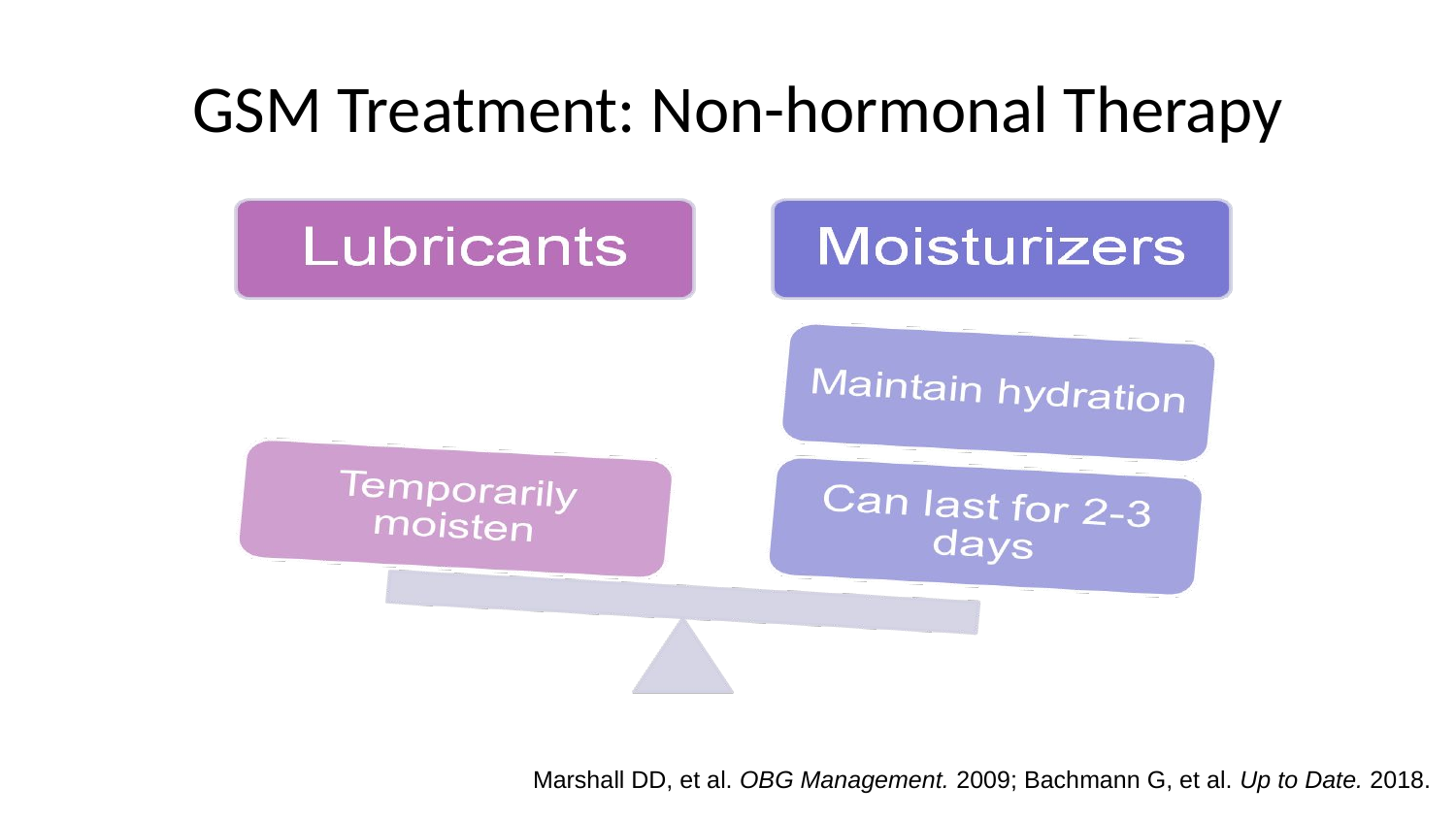

# GSM Treatment: Non-hormonal Therapy
Marshall DD, et al. OBG Management. 2009; Bachmann G, et al. Up to Date. 2018.

## Slide 19
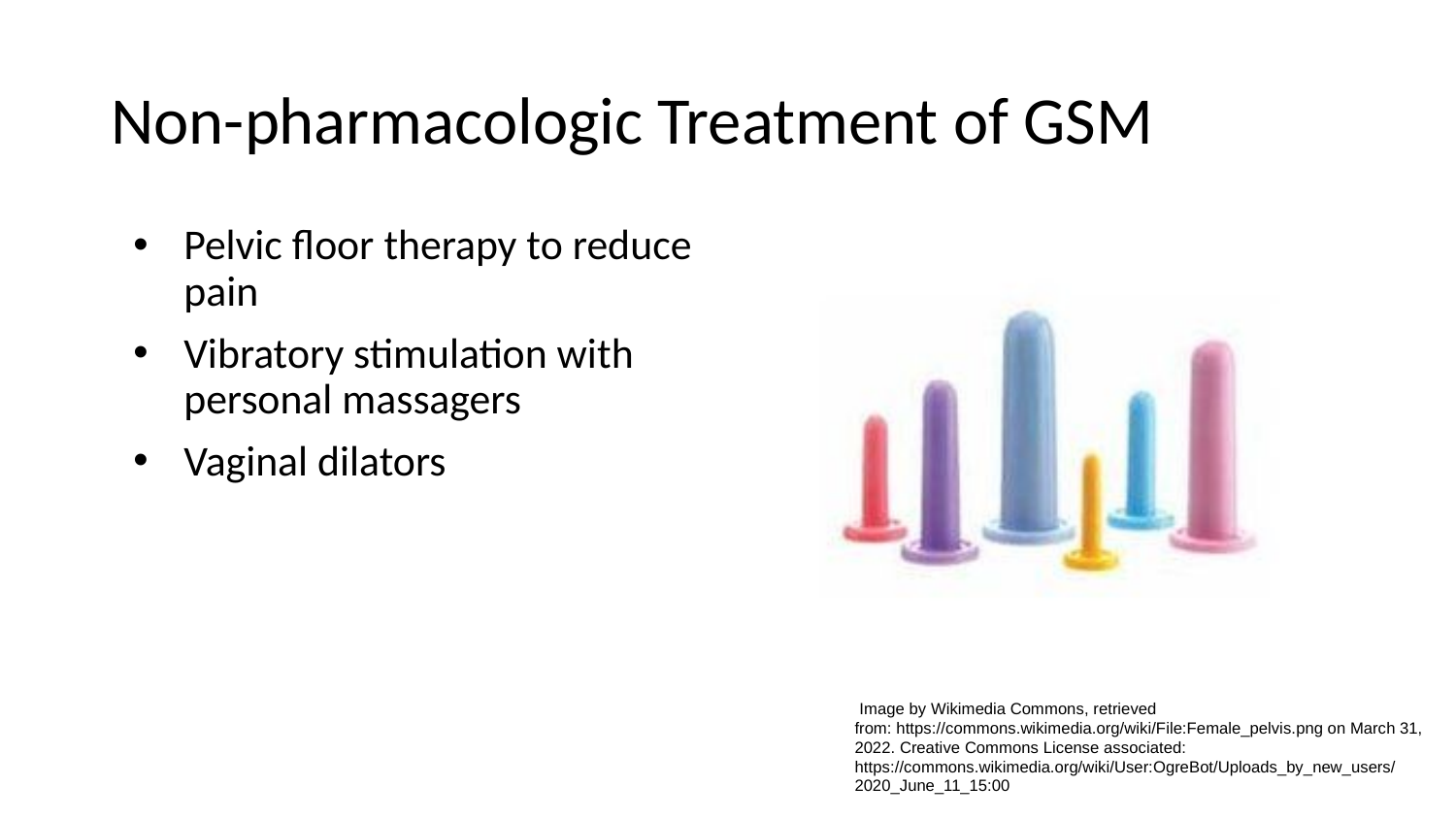

# Non-pharmacologic Treatment of GSM
Pelvic floor therapy to reduce pain
Vibratory stimulation with personal massagers
Vaginal dilators
 Image by Wikimedia Commons, retrieved from: https://commons.wikimedia.org/wiki/File:Female_pelvis.png on March 31, 2022. Creative Commons License associated: https://commons.wikimedia.org/wiki/User:OgreBot/Uploads_by_new_users/2020_June_11_15:00

## Slide 20
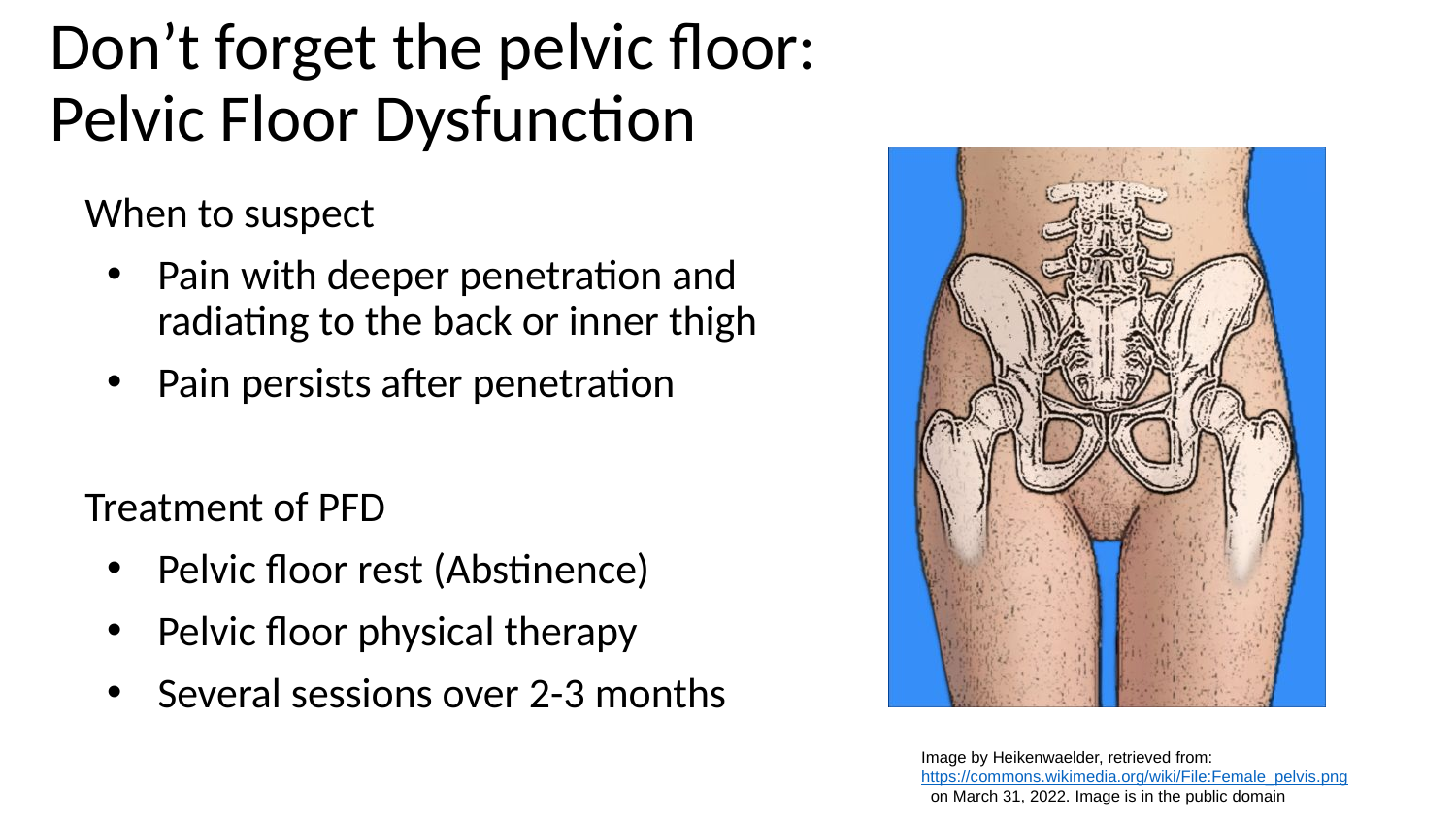

# Don’t forget the pelvic floor: Pelvic Floor Dysfunction
When to suspect
Pain with deeper penetration and radiating to the back or inner thigh
Pain persists after penetration
Treatment of PFD
Pelvic floor rest (Abstinence)
Pelvic floor physical therapy
Several sessions over 2-3 months
Image by Heikenwaelder, retrieved from: https://commons.wikimedia.org/wiki/File:Female_pelvis.png
 on March 31, 2022. Image is in the public domain

## Slide 21
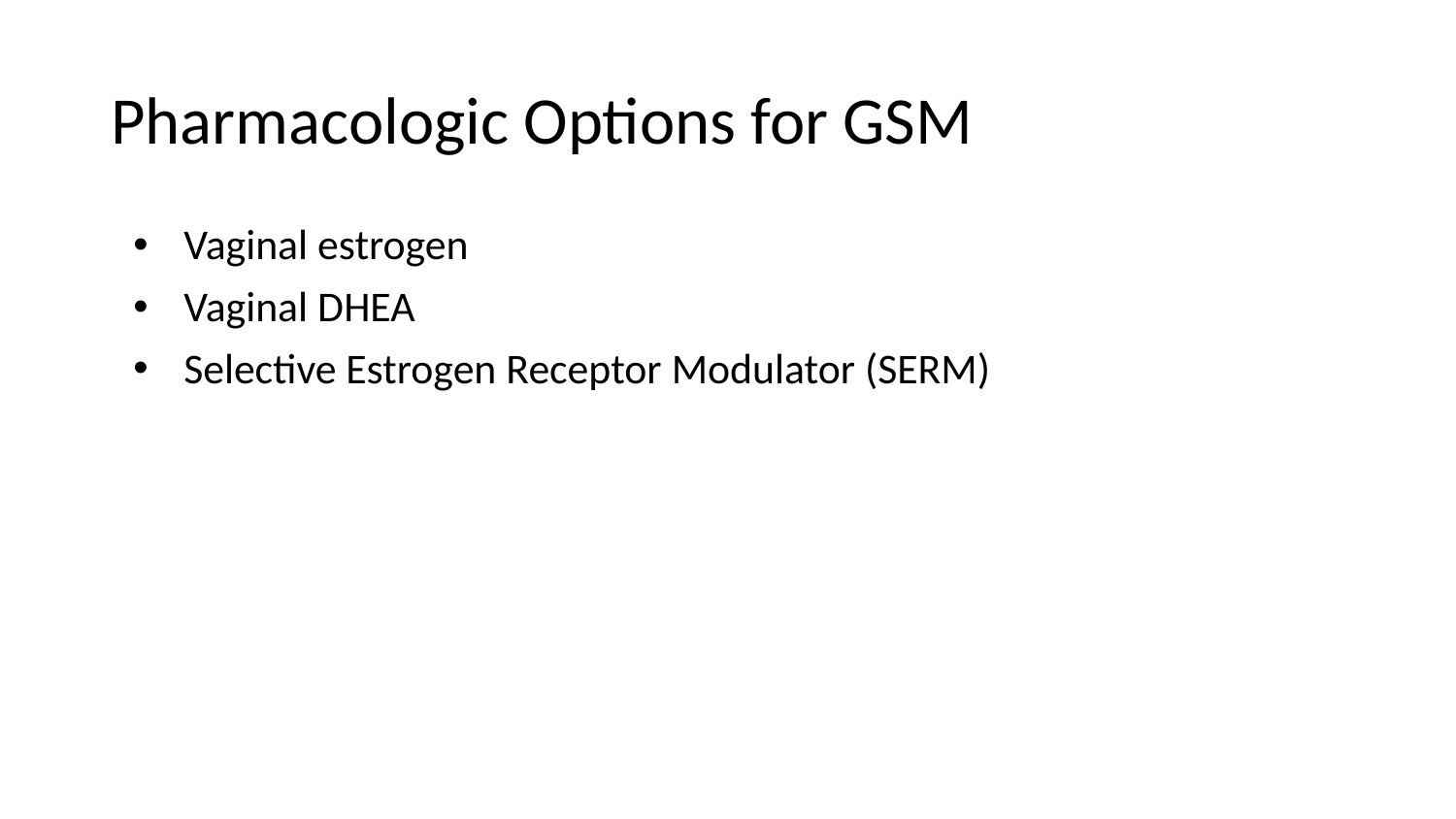

# Pharmacologic Options for GSM
Vaginal estrogen
Vaginal DHEA
Selective Estrogen Receptor Modulator (SERM)

## Slide 22
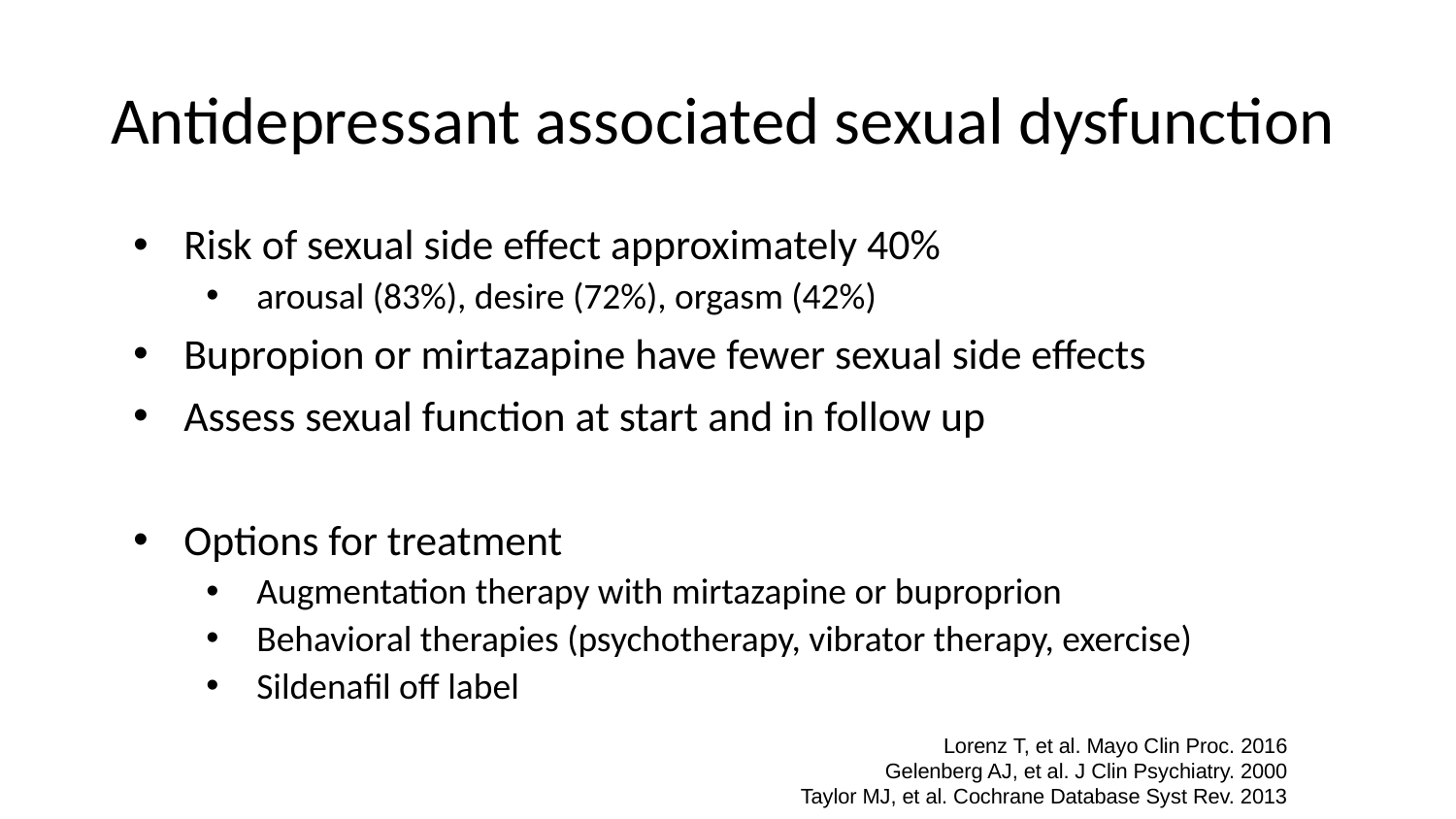

# Antidepressant associated sexual dysfunction
Risk of sexual side effect approximately 40%
arousal (83%), desire (72%), orgasm (42%)
Bupropion or mirtazapine have fewer sexual side effects
Assess sexual function at start and in follow up
Options for treatment
Augmentation therapy with mirtazapine or buproprion
Behavioral therapies (psychotherapy, vibrator therapy, exercise)
Sildenafil off label
Lorenz T, et al. Mayo Clin Proc. 2016
Gelenberg AJ, et al. J Clin Psychiatry. 2000
Taylor MJ, et al. Cochrane Database Syst Rev. 2013
.

## Slide 23
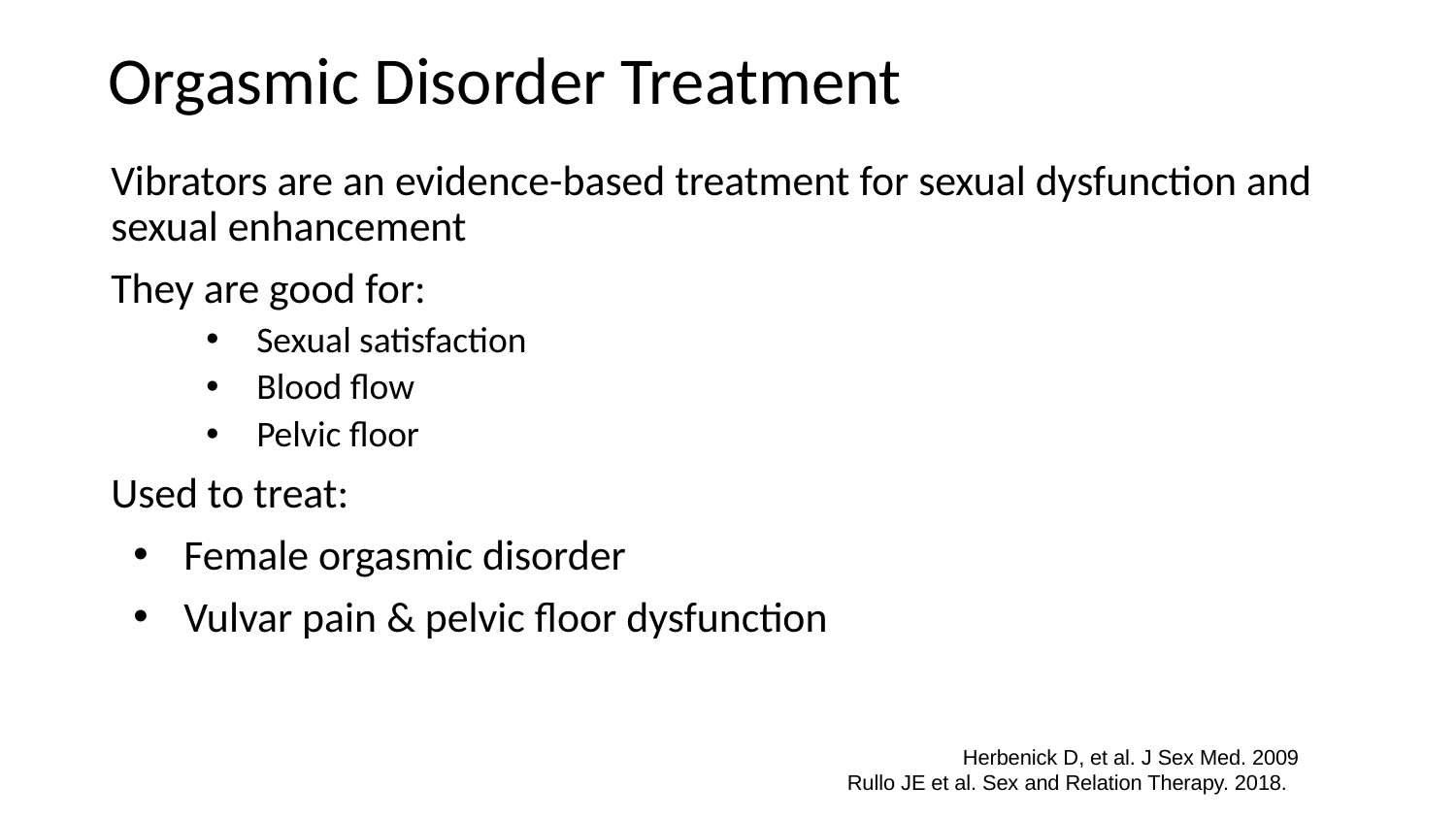

# Orgasmic Disorder Treatment
Vibrators are an evidence-based treatment for sexual dysfunction and sexual enhancement
They are good for:
Sexual satisfaction
Blood flow
Pelvic floor
Used to treat:
Female orgasmic disorder
Vulvar pain & pelvic floor dysfunction
Herbenick D, et al. J Sex Med. 2009
  Rullo JE et al. Sex and Relation Therapy. 2018.

## Slide 24
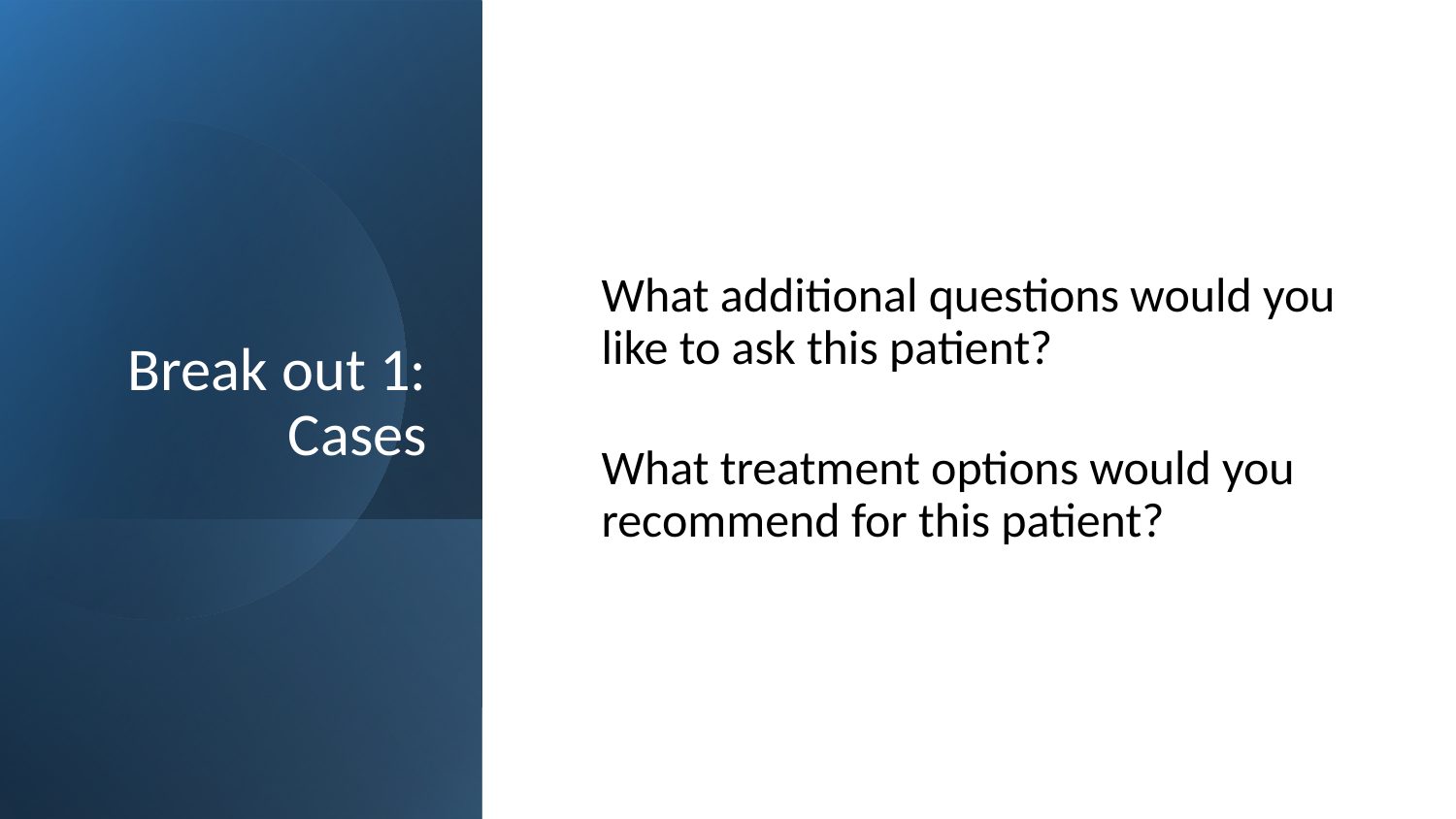

# Break out 1: Cases
What additional questions would you like to ask this patient?
What treatment options would you recommend for this patient?

## Slide 25
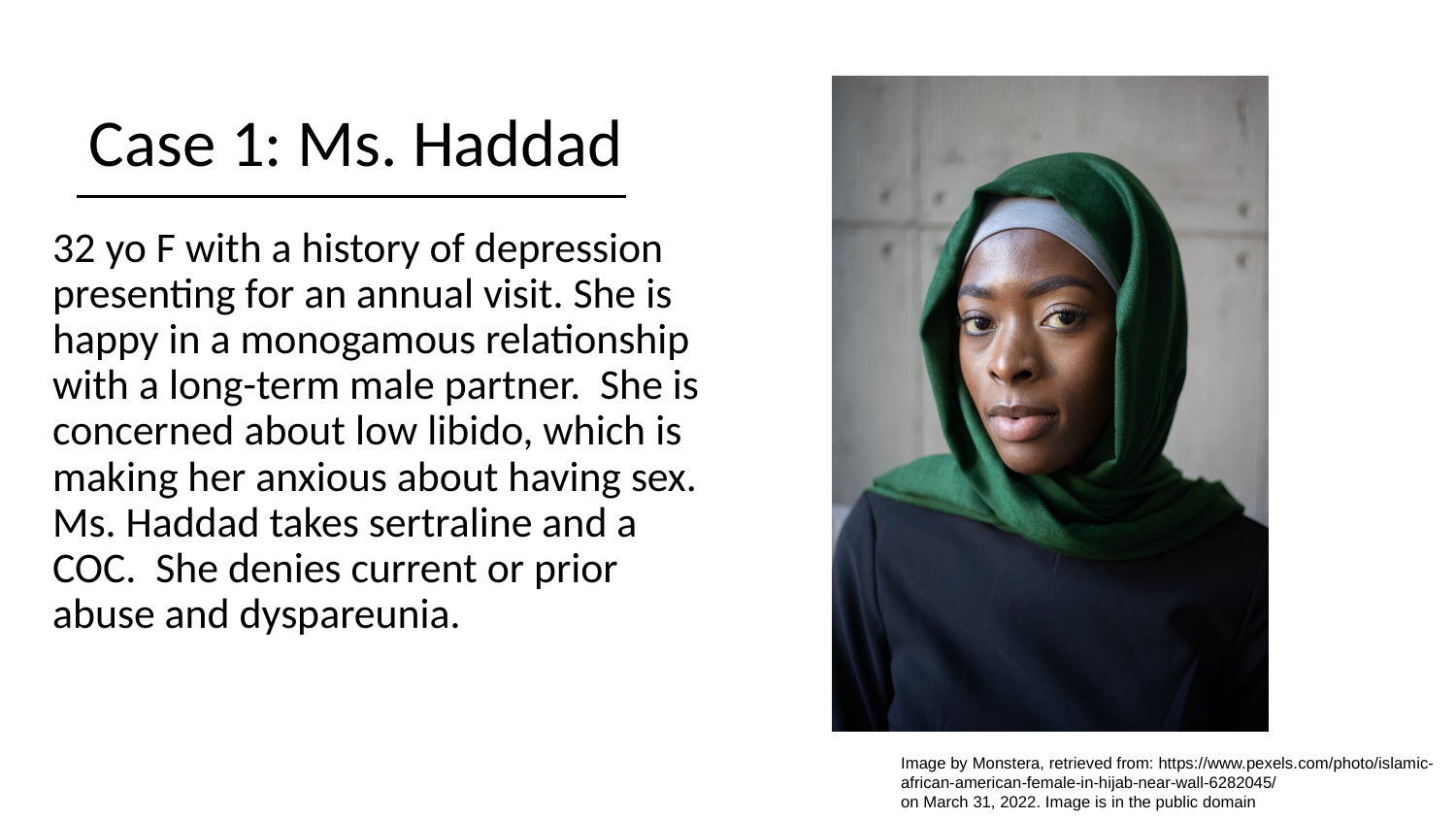

# Case 1: Ms. Haddad
32 yo F with a history of depression presenting for an annual visit. She is happy in a monogamous relationship with a long-term male partner. She is concerned about low libido, which is making her anxious about having sex. Ms. Haddad takes sertraline and a COC. She denies current or prior abuse and dyspareunia.
Image by Monstera, retrieved from: https://www.pexels.com/photo/islamic-african-american-female-in-hijab-near-wall-6282045/
on March 31, 2022. Image is in the public domain

## Slide 26
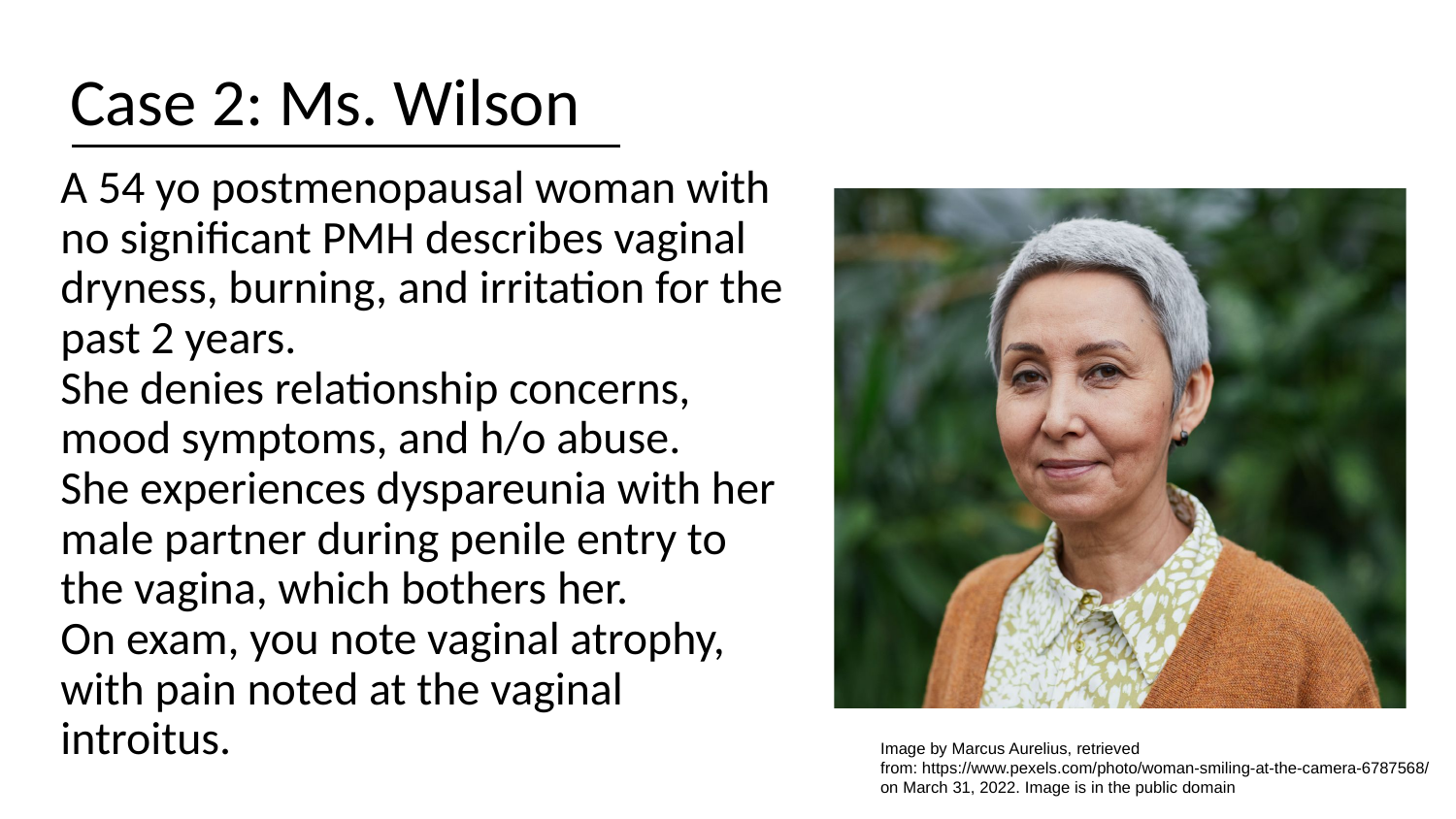

# Case 2: Ms. Wilson
A 54 yo postmenopausal woman with no significant PMH describes vaginal dryness, burning, and irritation for the past 2 years.
She denies relationship concerns, mood symptoms, and h/o abuse.
She experiences dyspareunia with her male partner during penile entry to the vagina, which bothers her.
On exam, you note vaginal atrophy, with pain noted at the vaginal introitus.
Image by Marcus Aurelius, retrieved from: https://www.pexels.com/photo/woman-smiling-at-the-camera-6787568/
on March 31, 2022. Image is in the public domain

## Slide 27
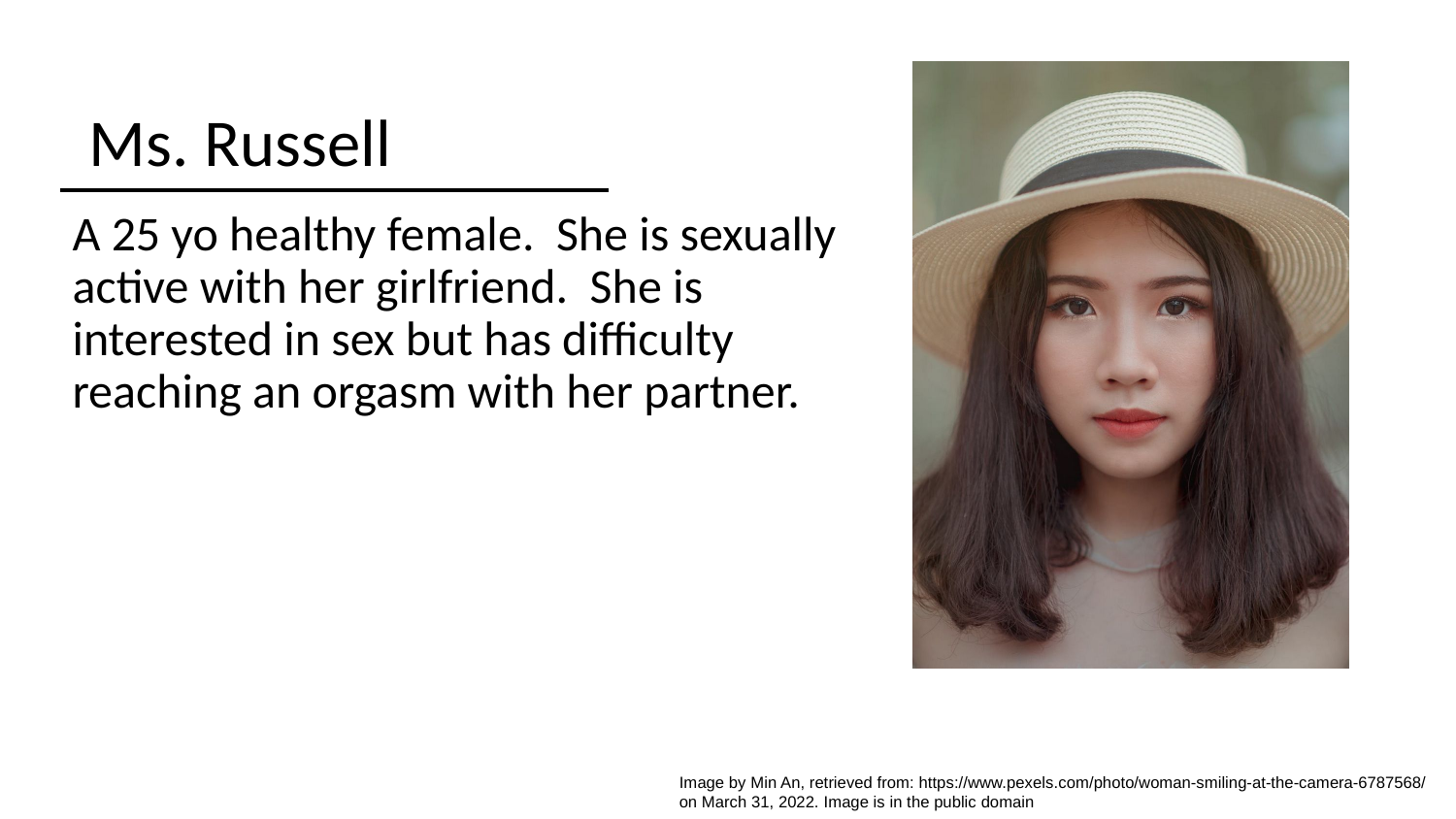

# Ms. Russell
A 25 yo healthy female. She is sexually active with her girlfriend. She is interested in sex but has difficulty reaching an orgasm with her partner.
Image by Min An, retrieved from: https://www.pexels.com/photo/woman-smiling-at-the-camera-6787568/
on March 31, 2022. Image is in the public domain

## Slide 28
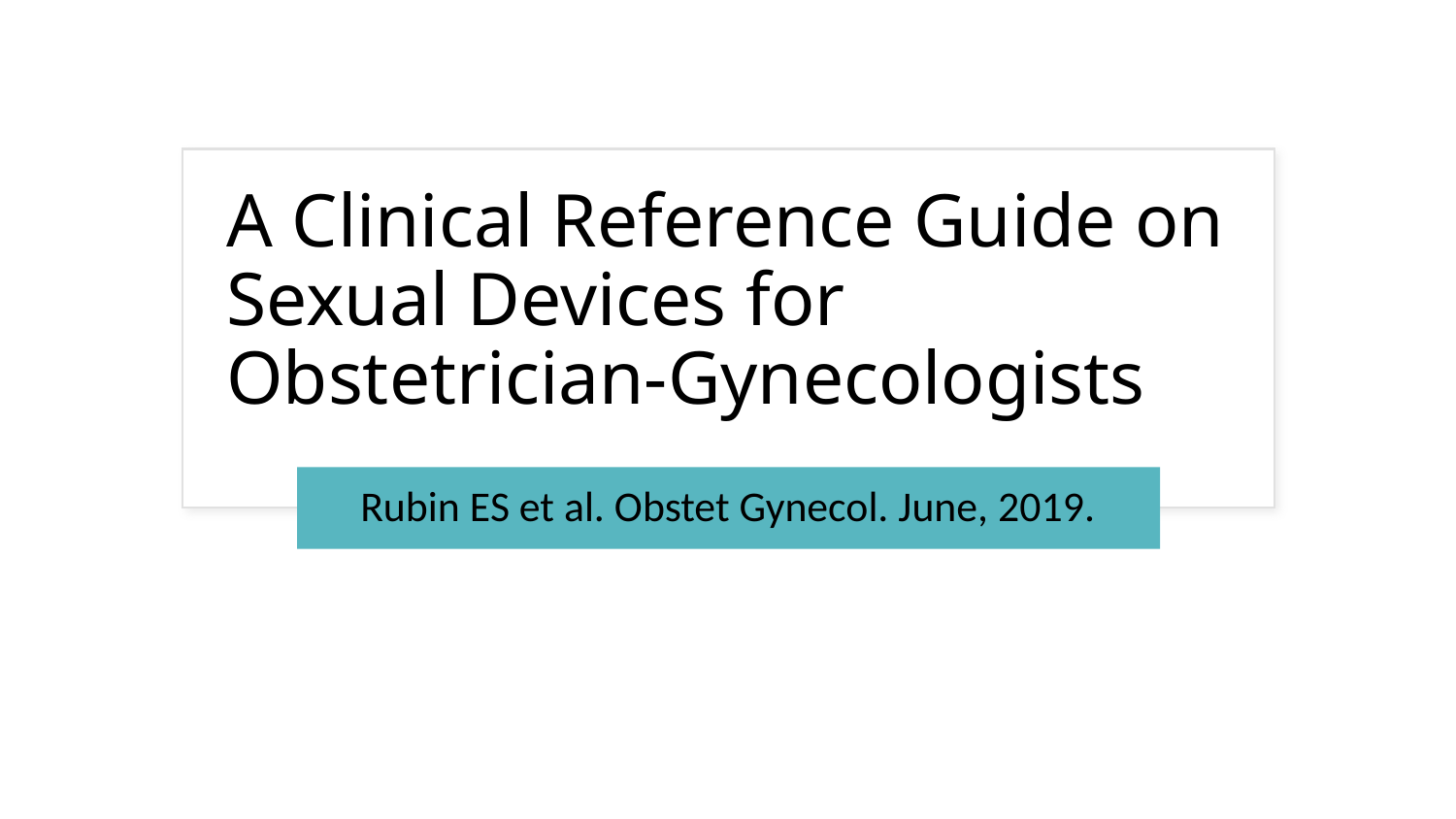

# A Clinical Reference Guide on Sexual Devices for Obstetrician-Gynecologists
Rubin ES et al. Obstet Gynecol. June, 2019.

## Slide 29
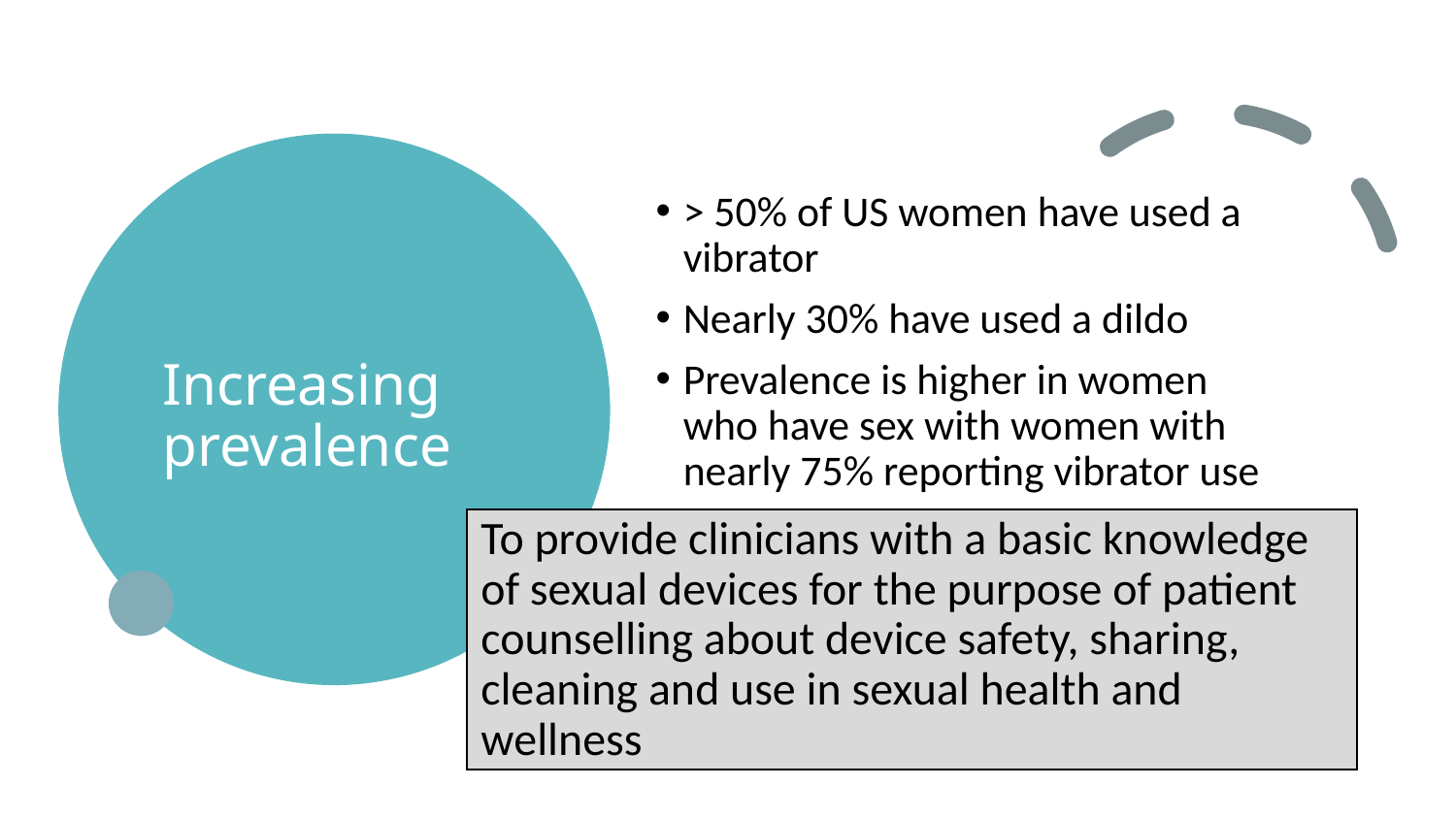

# Increasing prevalence
> 50% of US women have used a vibrator
Nearly 30% have used a dildo
Prevalence is higher in women who have sex with women with nearly 75% reporting vibrator use
To provide clinicians with a basic knowledge of sexual devices for the purpose of patient counselling about device safety, sharing, cleaning and use in sexual health and wellness

## Slide 30
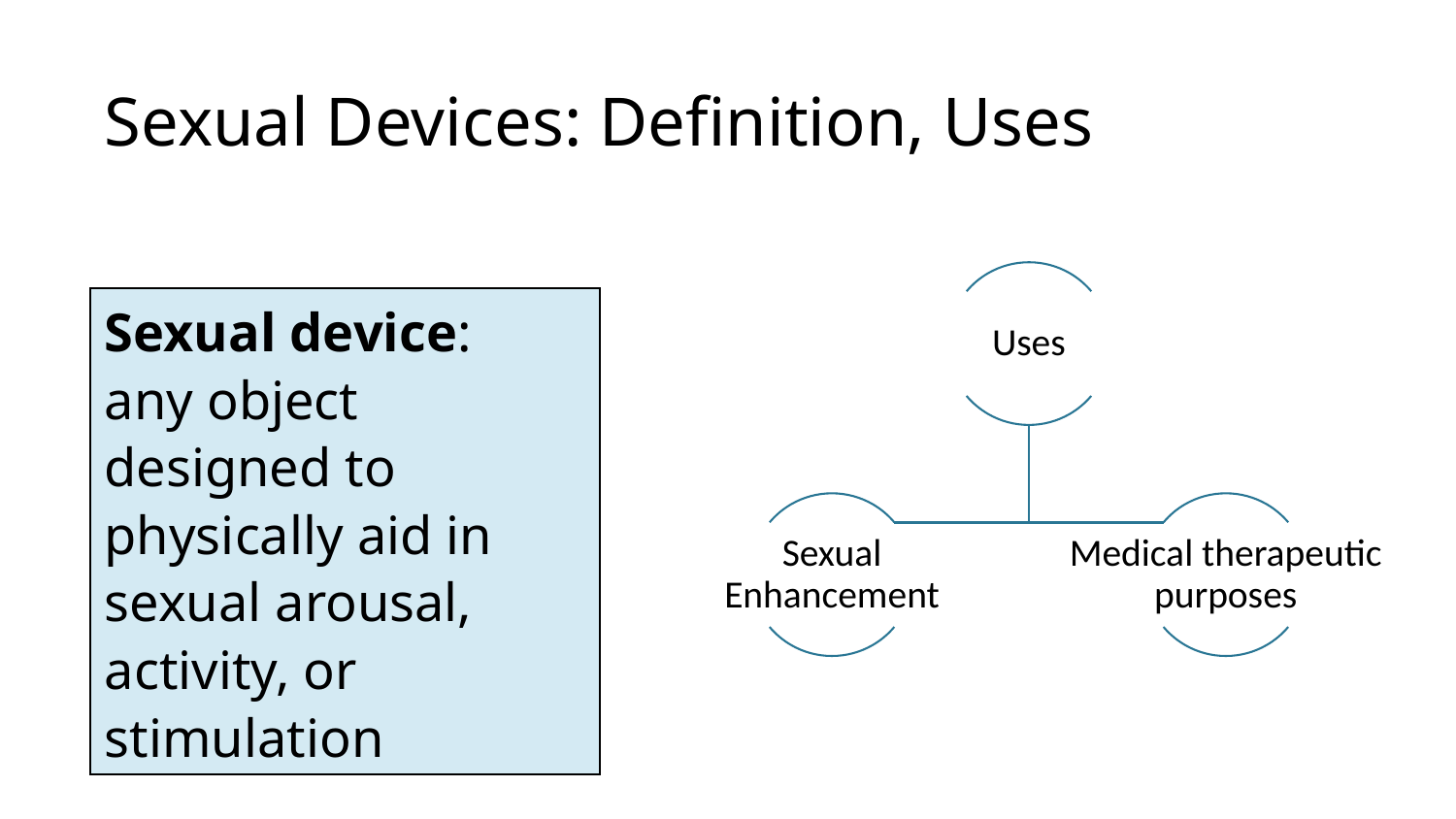

# Sexual Devices: Definition, Uses
Sexual device:
any object designed to physically aid in sexual arousal, activity, or stimulation

## Slide 31
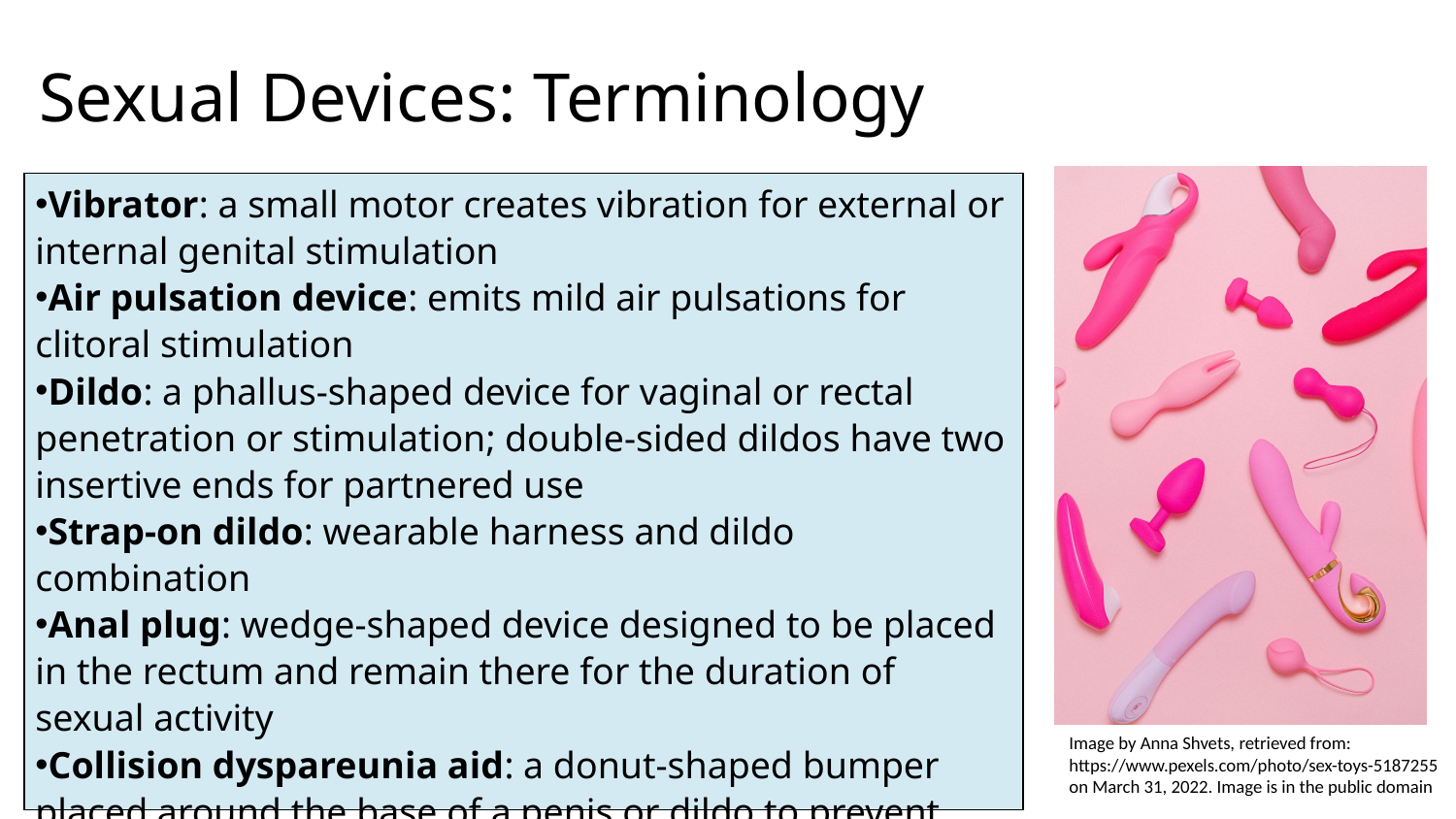

# Sexual Devices: Terminology
Vibrator: a small motor creates vibration for external or internal genital stimulation
Air pulsation device: emits mild air pulsations for clitoral stimulation
Dildo: a phallus-shaped device for vaginal or rectal penetration or stimulation; double-sided dildos have two insertive ends for partnered use
Strap-on dildo: wearable harness and dildo combination
Anal plug: wedge-shaped device designed to be placed in the rectum and remain there for the duration of sexual activity
Collision dyspareunia aid: a donut-shaped bumper placed around the base of a penis or dildo to prevent deep penetration; aka “penile ring”
Image by Anna Shvets, retrieved from: https://www.pexels.com/photo/sex-toys-5187255 on March 31, 2022. Image is in the public domain

## Slide 32
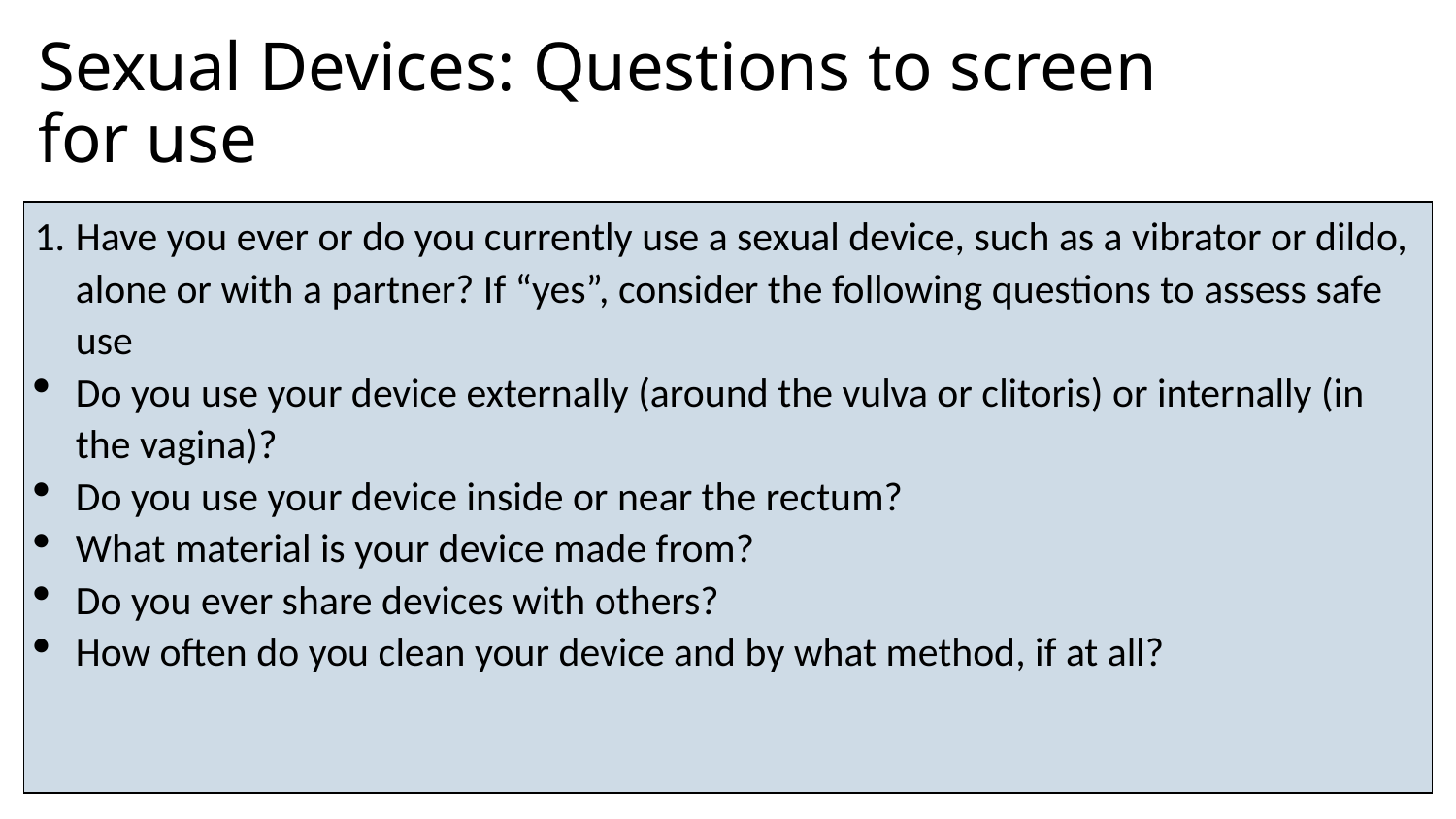

# Sexual Devices: Questions to screen for use
Have you ever or do you currently use a sexual device, such as a vibrator or dildo, alone or with a partner? If “yes”, consider the following questions to assess safe use
Do you use your device externally (around the vulva or clitoris) or internally (in the vagina)?
Do you use your device inside or near the rectum?
What material is your device made from?
Do you ever share devices with others?
How often do you clean your device and by what method, if at all?

## Slide 33
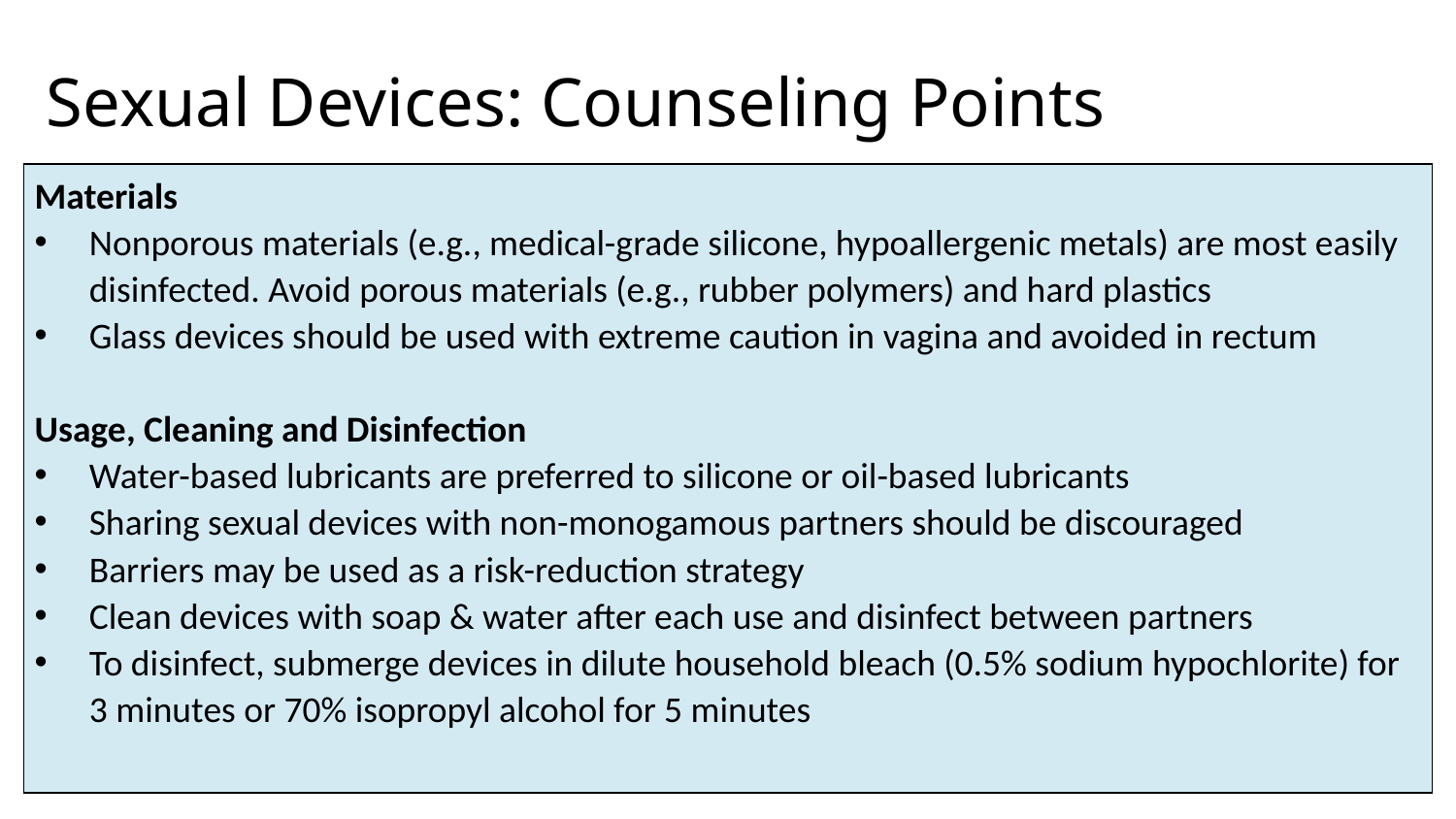

# Sexual Devices: Counseling Points
Materials
Nonporous materials (e.g., medical-grade silicone, hypoallergenic metals) are most easily disinfected. Avoid porous materials (e.g., rubber polymers) and hard plastics
Glass devices should be used with extreme caution in vagina and avoided in rectum
Usage, Cleaning and Disinfection
Water-based lubricants are preferred to silicone or oil-based lubricants
Sharing sexual devices with non-monogamous partners should be discouraged
Barriers may be used as a risk-reduction strategy
Clean devices with soap & water after each use and disinfect between partners
To disinfect, submerge devices in dilute household bleach (0.5% sodium hypochlorite) for 3 minutes or 70% isopropyl alcohol for 5 minutes

## Slide 34
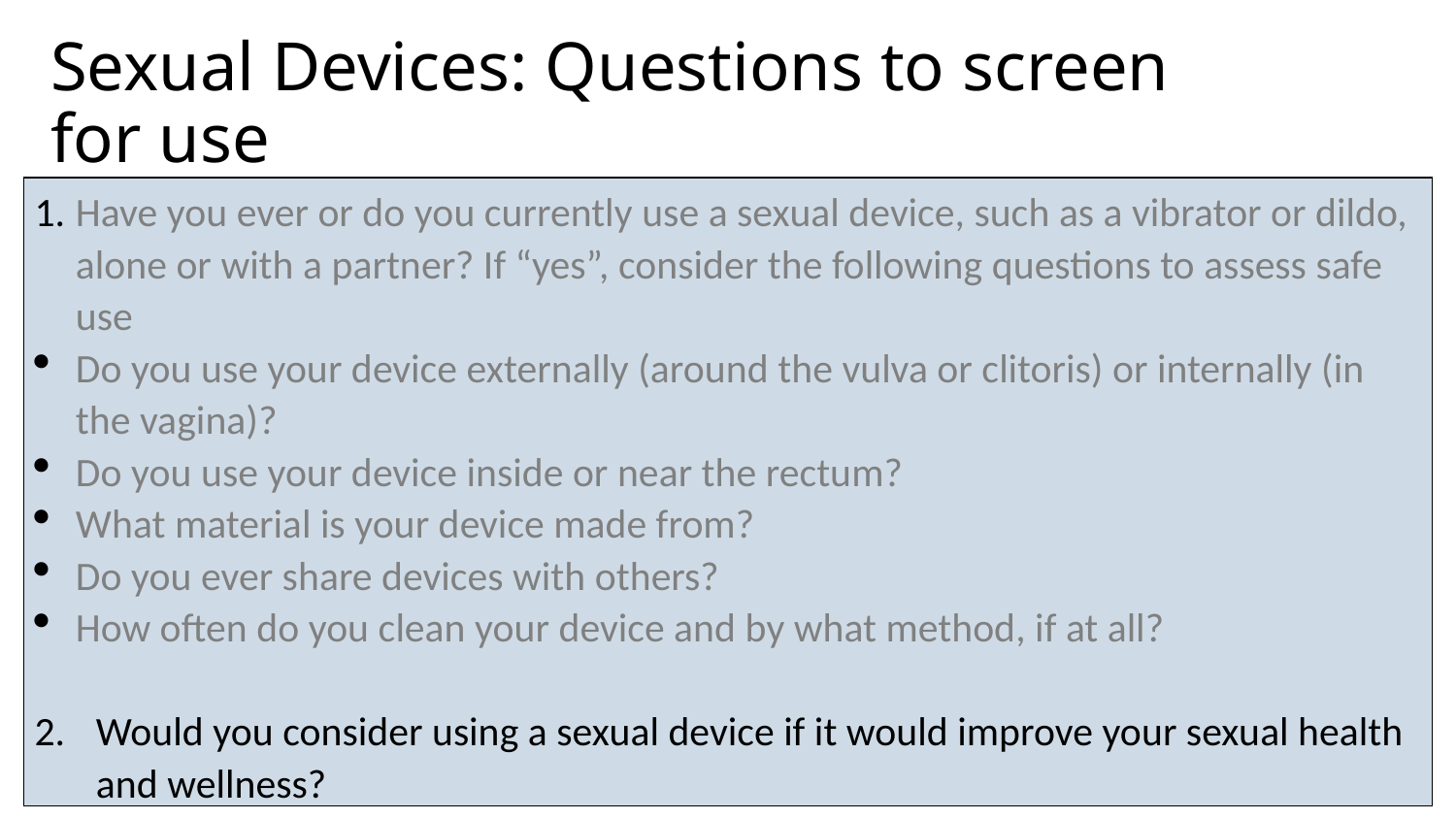

# Sexual Devices: Questions to screen for use
Have you ever or do you currently use a sexual device, such as a vibrator or dildo, alone or with a partner? If “yes”, consider the following questions to assess safe use
Do you use your device externally (around the vulva or clitoris) or internally (in the vagina)?
Do you use your device inside or near the rectum?
What material is your device made from?
Do you ever share devices with others?
How often do you clean your device and by what method, if at all?
Would you consider using a sexual device if it would improve your sexual health and wellness?

## Slide 35
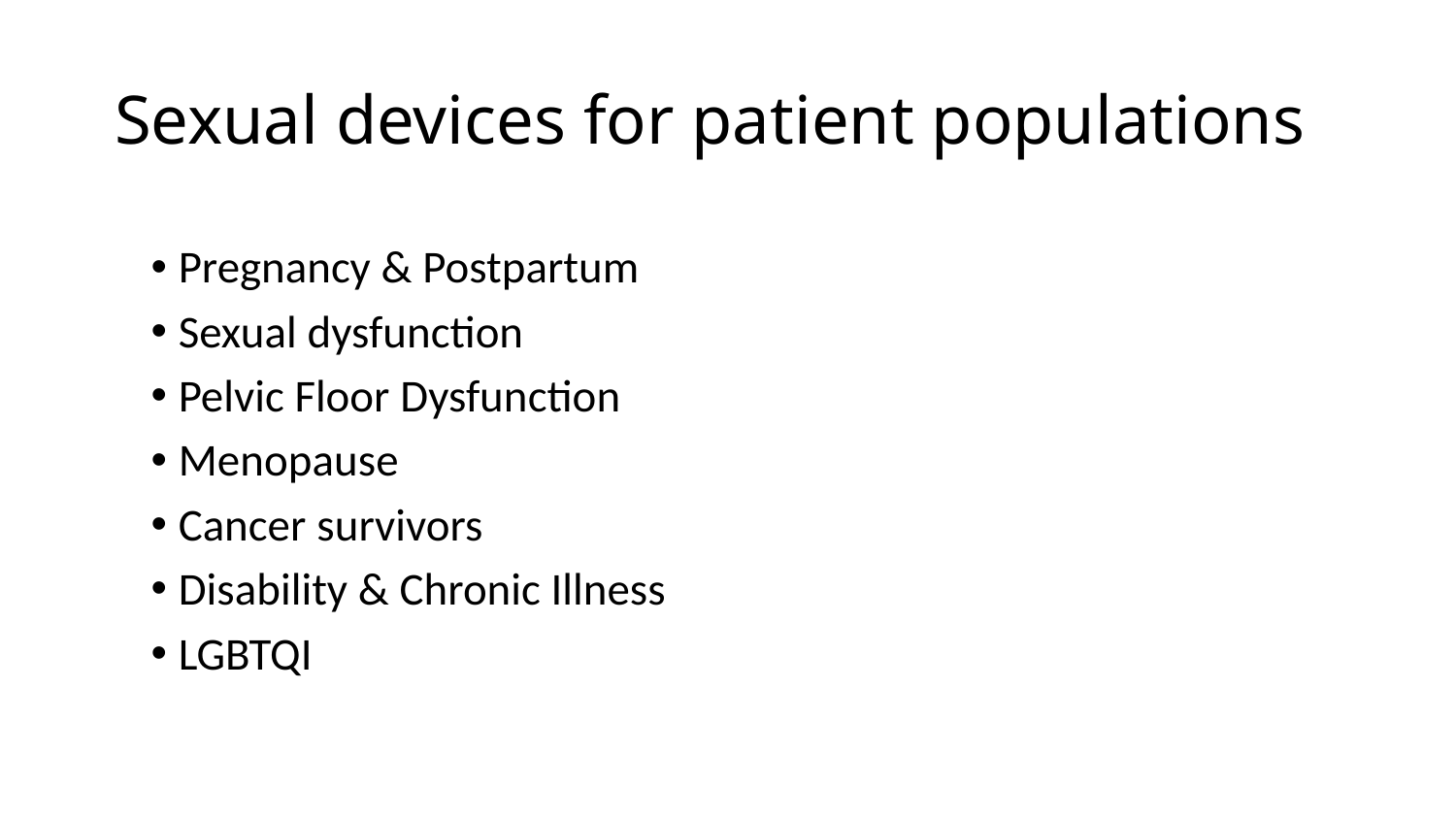

# Sexual devices for patient populations
Pregnancy & Postpartum
Sexual dysfunction
Pelvic Floor Dysfunction
Menopause
Cancer survivors
Disability & Chronic Illness
LGBTQI

## Slide 36
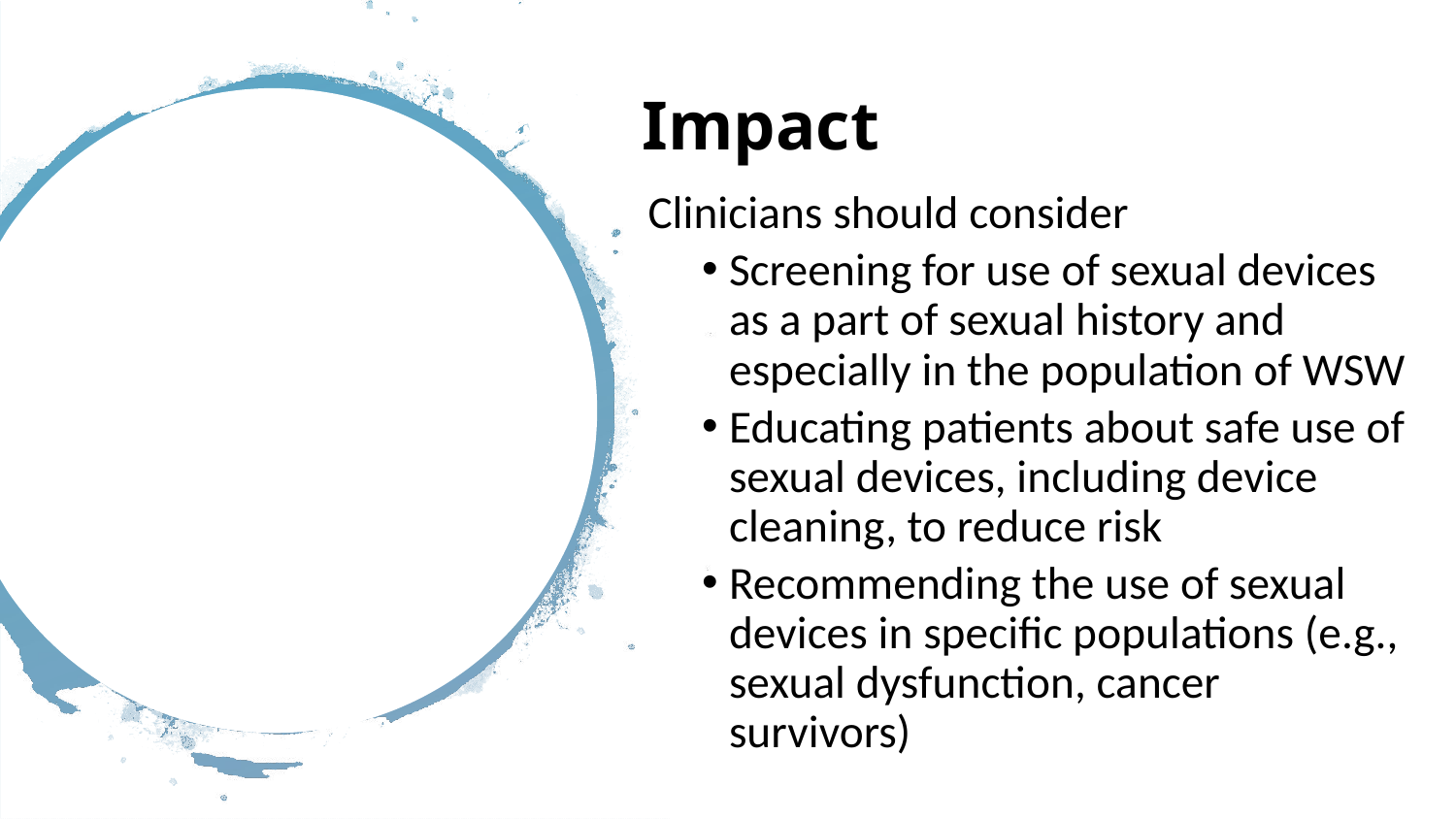

# Impact
Clinicians should consider
Screening for use of sexual devices as a part of sexual history and especially in the population of WSW
Educating patients about safe use of sexual devices, including device cleaning, to reduce risk
Recommending the use of sexual devices in specific populations (e.g., sexual dysfunction, cancer survivors)

## Slide 37
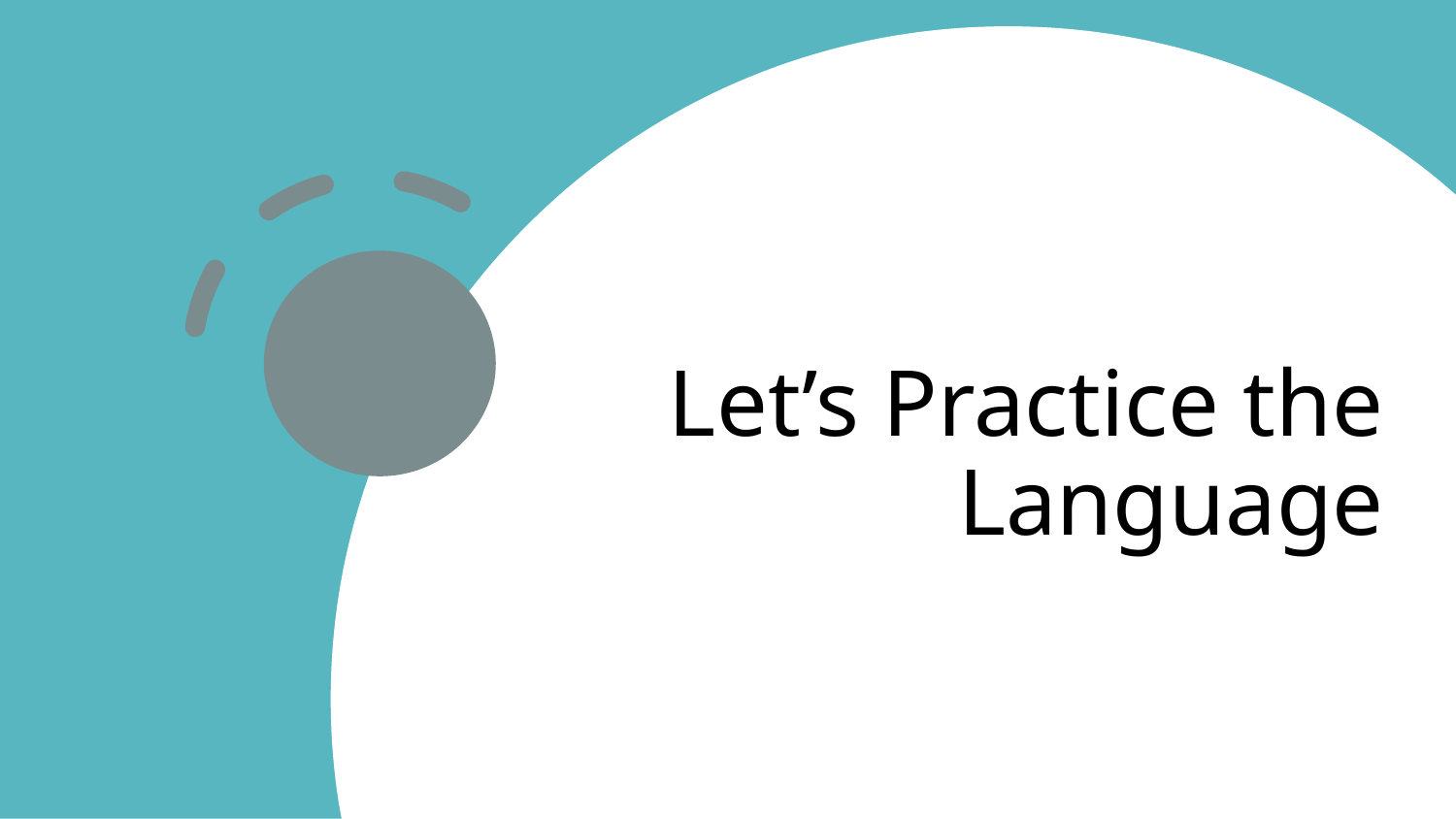

# Let’s Practice the Language

## Slide 38
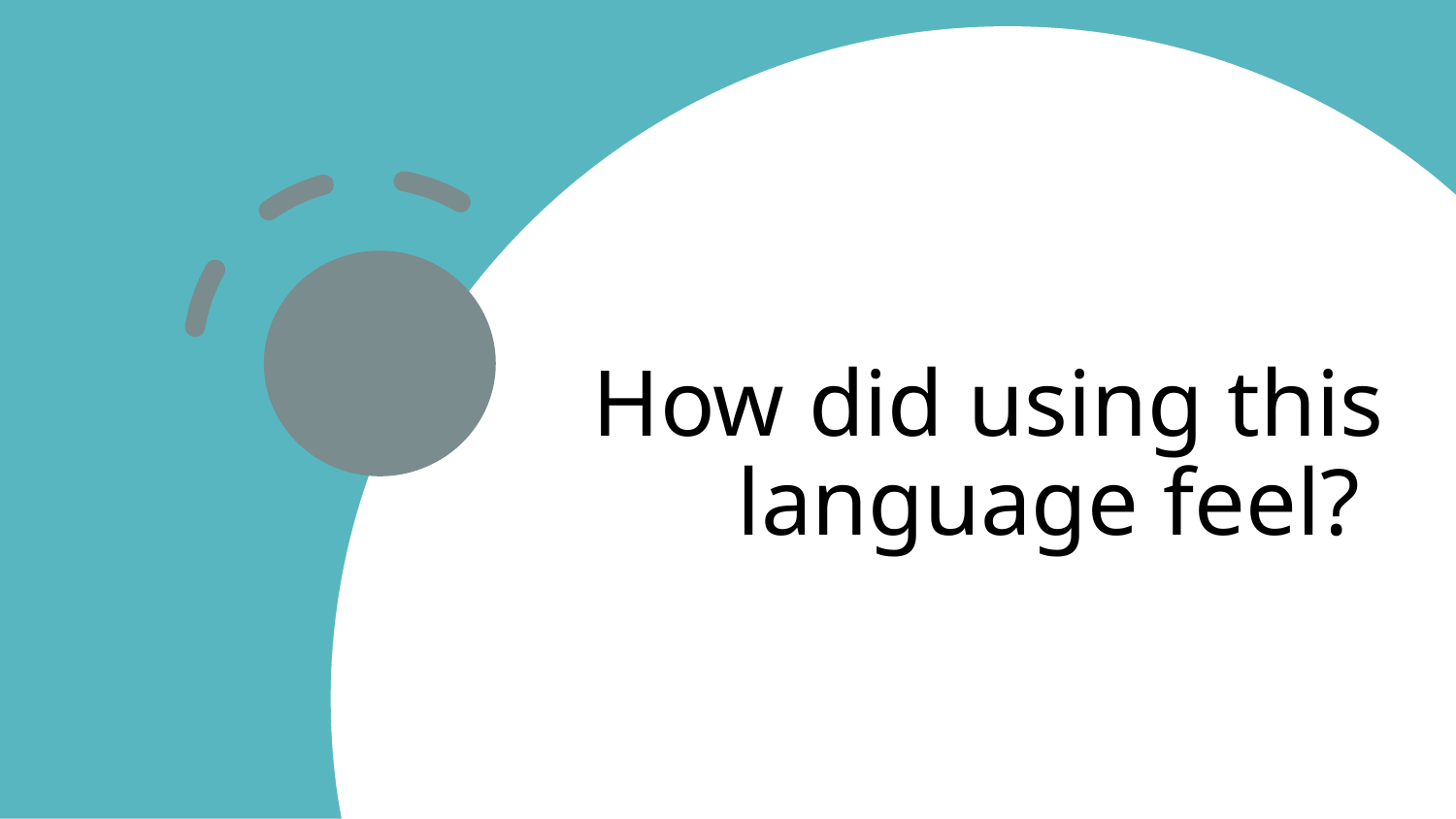

# How did using this language feel?

## Slide 39
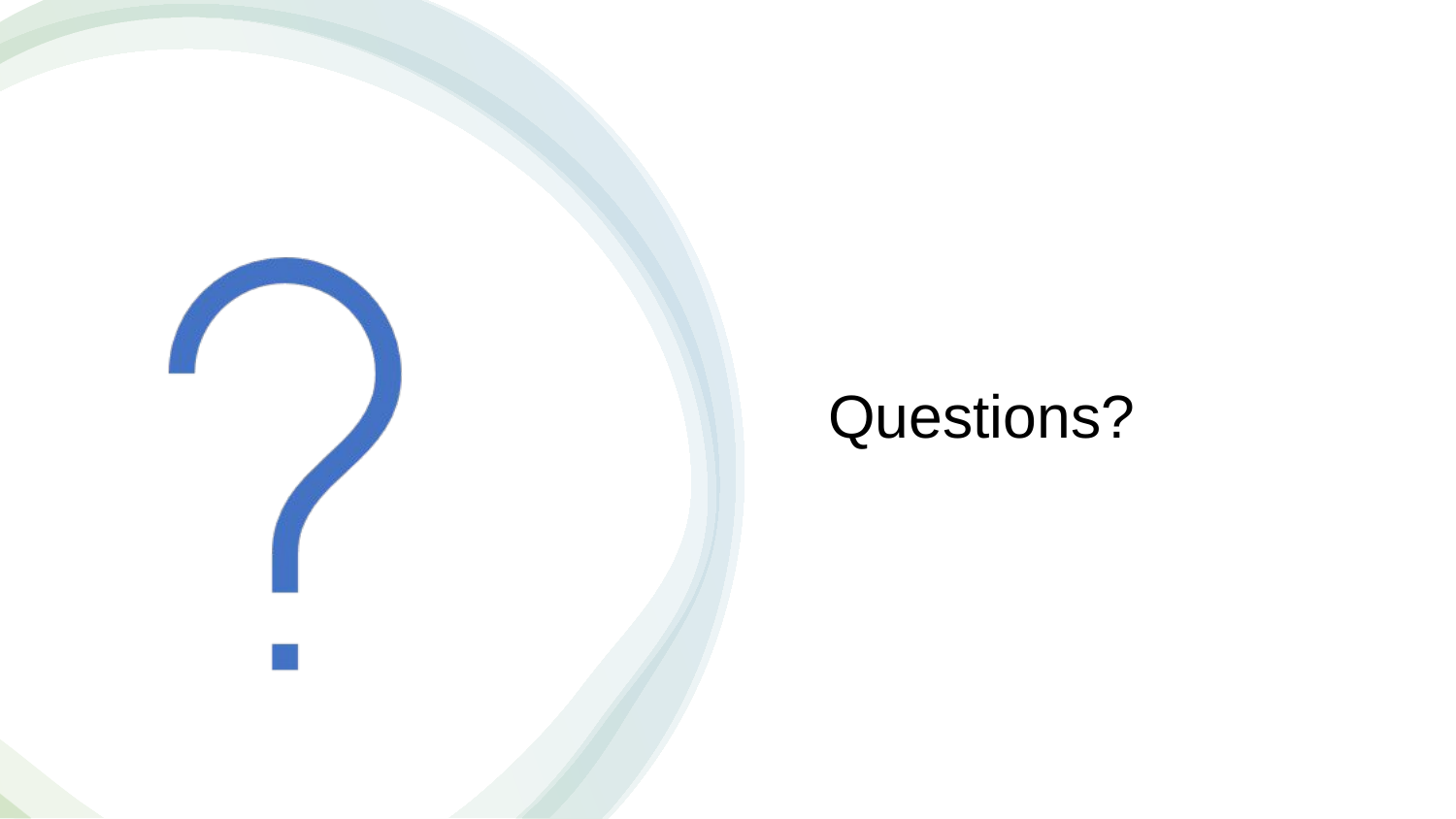

# Questions?

## Slide 40
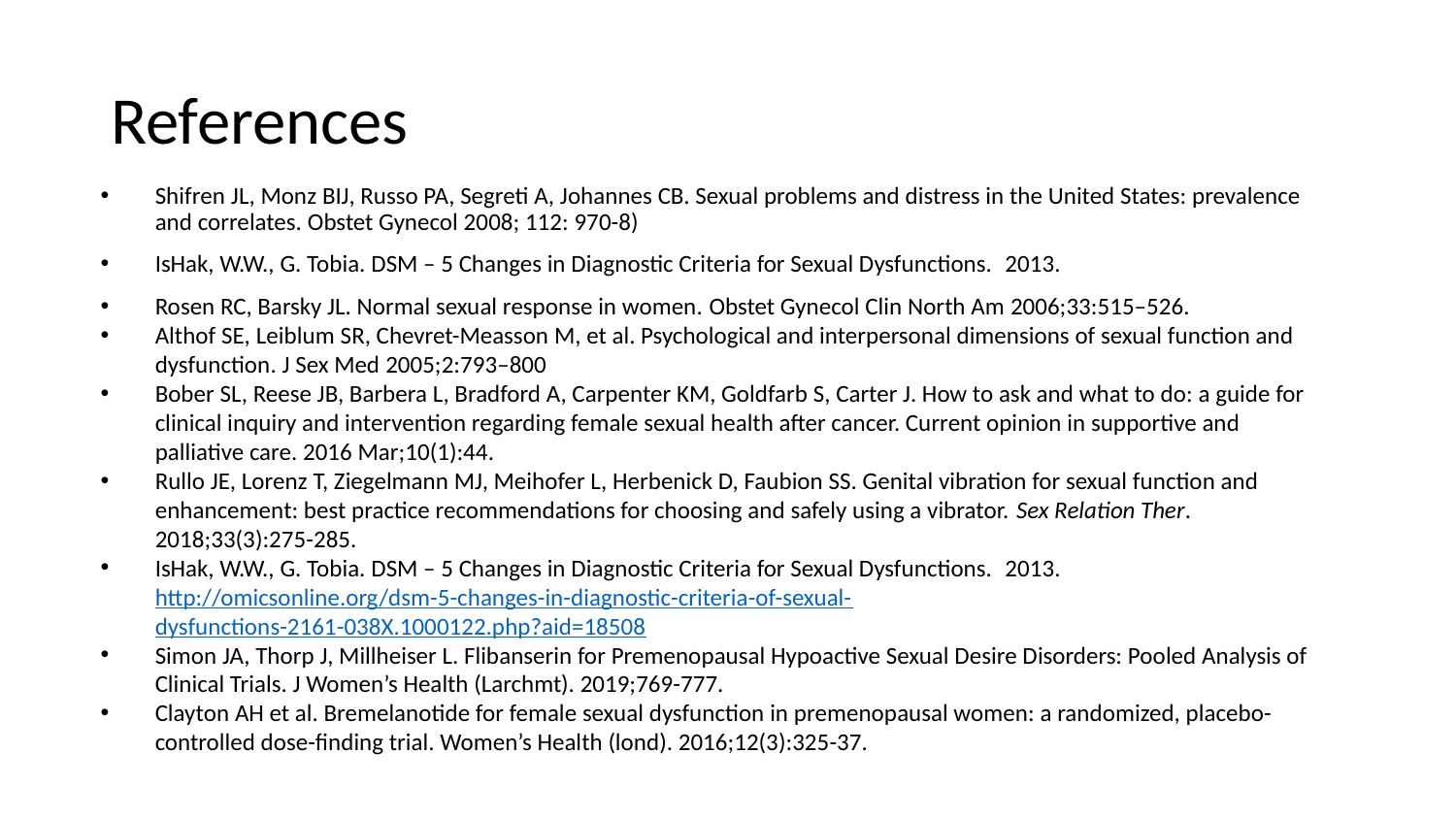

# References
Shifren JL, Monz BIJ, Russo PA, Segreti A, Johannes CB. Sexual problems and distress in the United States: prevalence and correlates. Obstet Gynecol 2008; 112: 970-8)
IsHak, W.W., G. Tobia. DSM – 5 Changes in Diagnostic Criteria for Sexual Dysfunctions.  2013.
Rosen RC, Barsky JL. Normal sexual response in women. Obstet Gynecol Clin North Am 2006;33:515–526.
Althof SE, Leiblum SR, Chevret-Measson M, et al. Psychological and interpersonal dimensions of sexual function and dysfunction. J Sex Med 2005;2:793–800
Bober SL, Reese JB, Barbera L, Bradford A, Carpenter KM, Goldfarb S, Carter J. How to ask and what to do: a guide for clinical inquiry and intervention regarding female sexual health after cancer. Current opinion in supportive and palliative care. 2016 Mar;10(1):44.
Rullo JE, Lorenz T, Ziegelmann MJ, Meihofer L, Herbenick D, Faubion SS. Genital vibration for sexual function and enhancement: best practice recommendations for choosing and safely using a vibrator. Sex Relation Ther. 2018;33(3):275-285.
IsHak, W.W., G. Tobia. DSM – 5 Changes in Diagnostic Criteria for Sexual Dysfunctions.  2013.  http://omicsonline.org/dsm-5-changes-in-diagnostic-criteria-of-sexual-dysfunctions-2161-038X.1000122.php?aid=18508
Simon JA, Thorp J, Millheiser L. Flibanserin for Premenopausal Hypoactive Sexual Desire Disorders: Pooled Analysis of Clinical Trials. J Women’s Health (Larchmt). 2019;769-777.
Clayton AH et al. Bremelanotide for female sexual dysfunction in premenopausal women: a randomized, placebo-controlled dose-finding trial. Women’s Health (lond). 2016;12(3):325-37.

## Slide 41
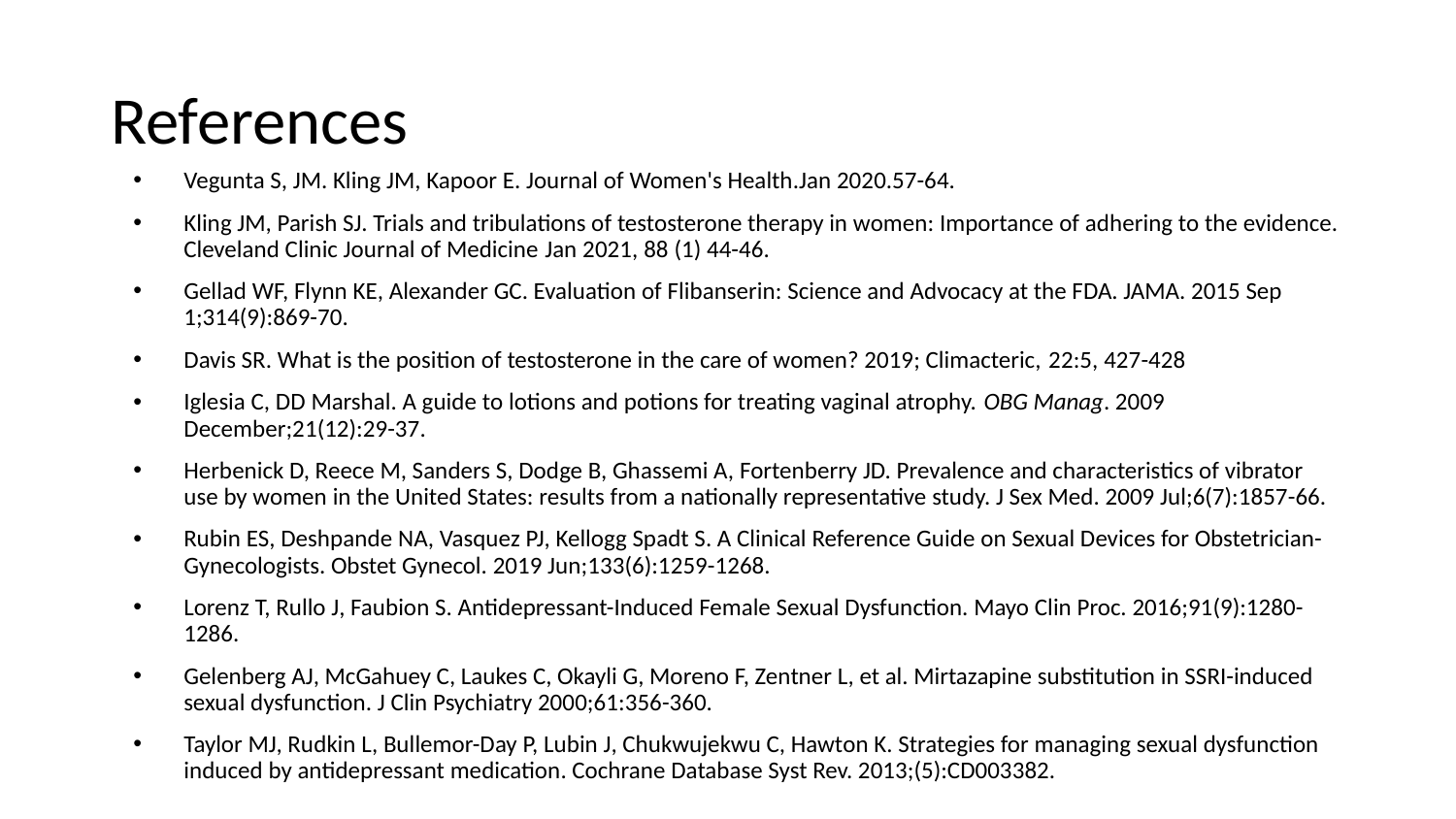

# References
Vegunta S, JM. Kling JM, Kapoor E. Journal of Women's Health.Jan 2020.57-64.
Kling JM, Parish SJ. Trials and tribulations of testosterone therapy in women: Importance of adhering to the evidence. Cleveland Clinic Journal of Medicine Jan 2021, 88 (1) 44-46.
Gellad WF, Flynn KE, Alexander GC. Evaluation of Flibanserin: Science and Advocacy at the FDA. JAMA. 2015 Sep 1;314(9):869-70.
Davis SR. What is the position of testosterone in the care of women? 2019; Climacteric, 22:5, 427-428
Iglesia C, DD Marshal. A guide to lotions and potions for treating vaginal atrophy. OBG Manag. 2009 December;21(12):29-37.
Herbenick D, Reece M, Sanders S, Dodge B, Ghassemi A, Fortenberry JD. Prevalence and characteristics of vibrator use by women in the United States: results from a nationally representative study. J Sex Med. 2009 Jul;6(7):1857-66.
Rubin ES, Deshpande NA, Vasquez PJ, Kellogg Spadt S. A Clinical Reference Guide on Sexual Devices for Obstetrician-Gynecologists. Obstet Gynecol. 2019 Jun;133(6):1259-1268.
Lorenz T, Rullo J, Faubion S. Antidepressant-Induced Female Sexual Dysfunction. Mayo Clin Proc. 2016;91(9):1280-1286.
Gelenberg AJ, McGahuey C, Laukes C, Okayli G, Moreno F, Zentner L, et al. Mirtazapine substitution in SSRI-induced sexual dysfunction. J Clin Psychiatry 2000;61:356-360.
Taylor MJ, Rudkin L, Bullemor-Day P, Lubin J, Chukwujekwu C, Hawton K. Strategies for managing sexual dysfunction induced by antidepressant medication. Cochrane Database Syst Rev. 2013;(5):CD003382.
