# Supplementary material for: Female Sexual Dysfunction: A Primer for Primary Care Health Professionals
Source: MedEdPORTAL. 2023 Apr 25;19:11312. doi: 10.15766/mep_2374-8265.11312 (PMC10126124; doi:10.15766/mep_2374-8265.11312)
Supplement: Supplementary file 1 — 60-Minute Didactic.pptx90-Minute Workshop.pptxDiscussion Cases.docxSexual Devices Language Drills.docxRole-Play Script.docxEvaluation.docx [file mep_2374-8265.11312-s001.zip › C. Discussion Cases.docx]

**Female Sexual Dysfunction: A Primer for Primary Care Clinicians**

Discussion Cases

For the case assigned to your group, discuss the following questions:

- What additional questions would you like to ask this patient?
- What treatment options would you recommend for this patient?

**Case 1: Ms. Haddad**

32 yo F with a history of depression presenting for an annual visit. She is happy in a monogamous relationship with a long-term male partner. She is concerned about low libido, which is making her anxious about having sex. Ms. Haddad takes sertraline and a COC. She denies current or prior abuse and dyspareunia.

**Case 2: Ms. Wilson**

A 54 yo postmenopausal woman with no significant PMH describes vaginal dryness, burning, and irritation for the past 2 years.

She denies relationship concerns, mood symptoms, and h/o abuse.

She experiences dyspareunia with her male partner during penile entry to the vagina, which bothers her.

On exam, you note vaginal atrophy, with pain noted at the vaginal introitus

**Case 3: Ms. Russell**

A 25 yo healthy female. She is sexually active with her girlfriend. She is interested in sex but has difficulty reaching an orgasm with her partner.
