# Supplementary material for: Female Sexual Dysfunction: A Primer for Primary Care Health Professionals
Source: MedEdPORTAL. 2023 Apr 25;19:11312. doi: 10.15766/mep_2374-8265.11312 (PMC10126124; doi:10.15766/mep_2374-8265.11312)
Supplement: Supplementary file 1 — 60-Minute Didactic.pptx90-Minute Workshop.pptxDiscussion Cases.docxSexual Devices Language Drills.docxRole-Play Script.docxEvaluation.docx [file mep_2374-8265.11312-s001.zip › D. Sexual Devices Language Drills.docx]

# With a partner, read out loud the following patient counselling scenarios verbatim. Take turns, so that each of you has the opportunity to speak as both the doctor and the patient.

**Scenario 1**

| **Doctor** | **Patient** |
| --- | --- |
| Thank you so much again for taking some time during our visit today to talk with me about the changes in libido you’ve been noticing. | Thank you for asking – no doctor has ever spoken with me about things like this before |
| Of course! Sex is a very important part of many peoples’ relationships. If I may ask one other question – have you ever or do you currently use a sexual device like a vibrator or a dildo – either alone or with your partner? | No, I haven’t. I’ve seen them advertised and on movies but thinking about buy one makes me feel little nervous and a little awkward. |
| I would never recommend that you do anything that makes you uncomfortable, but I can share that the use of sexual devices is common. As many as 50% of women have used a vibrator. Would you consider using a sexual device if it would help your sexual health and wellness? | I guess… if you think it would help me with the pain that I’ve been having with sex I would definitely consider trying it. Nothing else has been working! |
| Yes -- I think you could consider a couple of things to help, in addition to seeing the pelvic floor physical therapist like we discussed. Since insertive sex is too painful for you right now, you might consider trying out an external clitoral stimulation device, like a vibrator or air pulsation device, to help with lubrication and orgasm. | That makes sense. It’s helpful to know that this is something that many women use to help with sex. |
| Absolutely. The other thing that I will mention is that sometimes, vibrators or dildos can be used as alternatives to medical dilators. If a dilator is recommended by your pelvic floor physical therapist, you can definitely ask your therapist more about sexual devices during your session. | Okay, I will think about this more when I get home, and I will talk to my pelvic floor therapist about it… since you don’t think that would make them feel uncomfortable. |
| Sounds great. And if you have any questions or concerns, please don’t hesitate to call me in the office. | Thanks so much |

**Scenario 2**

| **Doctor** | **Patient** |
| --- | --- |
| Thank you so much again for taking some time during our visit today to talk with me about the changes in libido you’ve been noticing. | Thank you for asking – no doctor has ever spoken with me about things like this before |
| Of course! Sex is a very important part of many peoples’ relationships. If I may ask one other question – have you ever or do you currently use a sexual device like a vibrator or a dildo – either alone or with your partner? | Yes, I have used a vibrator before to help me orgasm. Usually I use it with my partner, Shelby, but sometimes I do use it by myself. |
| Thank you, that’s helpful for me to know and something that I hear commonly from my patients. Would it be okay if I asked you some additional questions about how you use your vibrator? I ask a these to all my patients who use sexual devices to make sure they’re being used as safely as possible. | Sure |
| Do you use the vibrator externally around the vagina or clitoris or inside the vagina? | We both use it on the outside around the clitoris. You know, to help with orgasm like I mentioned |
| That makes sense. And do you ever use it inside or near the rectum? | No, we don’t use it like that. |
| OK. And last question - do you share your vibrator with your partner, or with others? | My partner and I will share it sometimes. |
| Sure. It is very reasonable to share with a monogamous partner. In that case, I usually advise that you clean the device after every use with mild soap and warm water. Some devices are waterproof and can be submerged, but if yours isn’t then I recommend wiping it down with a washcloth. | That makes sense |
| And, just in the event that your relationship changes in the future, I should also say that I do not recommend sharing sexual devices with non-monogamous partners. It is best to disinfect the vibrator or other device between partners and use a barrier method like a condom to reduce risk of developing an infection. | Ok I will keep that in mind. That makes a lot of sense… I hasn’t really considered this before so thank you for bringing it up! |
