# Supplementary material for: Female Sexual Dysfunction: A Primer for Primary Care Health Professionals
Source: MedEdPORTAL. 2023 Apr 25;19:11312. doi: 10.15766/mep_2374-8265.11312 (PMC10126124; doi:10.15766/mep_2374-8265.11312)
Supplement: Supplementary file 1 — 60-Minute Didactic.pptx90-Minute Workshop.pptxDiscussion Cases.docxSexual Devices Language Drills.docxRole-Play Script.docxEvaluation.docx [file mep_2374-8265.11312-s001.zip › E. Role-Play Script.docx]

Female Sexual Dysfunction – A Primer for Primary Care Clinicians

Role Play Script: 5 A’s and a biopsychosocial approach

Layla: facilitator 1

Dr.: facilitator 2

Background: Layla is a 30-year-old woman in primary care clinic for an annual exam. She has no major past medical history, and is not currently taking any medications. During the sexual history, we find out that she’s in a 3-year relationship with her fiancé Connor. They are sexually active and monogamous, and using condoms and natural family planning for contraception.

Dr: So you’re sexually active with your fiancé Connor. Sexual problems can actually be pretty common. Do you have any problems with regards to sex?

Layla: Hmm, things could definitely be better.

Dr.: Tell me more.

Layla: Well, over the past year or so my libido has just tanked! I used to want to have sex at least one or two times a week, but now it seems like I’m just not interested.

Dr.: It sounds like you’ve noticed a big change.

Layla: Definitely. And don’t get me wrong, Connor is very sweet and understanding, but I do worry that this could have a negative effect on our relationship down the road.

Dr.: Yes, sex is a very important part of many peoples’ relationships!

Layla: Everything else about our relationship is great. So if I could just get that spark back…

Dr.: Let’s talk some more so we can figure out how to best help you.

*Pause for reflection – What did you notice?*

Dr.: So tell me more about the change in your libido you’ve noticed.

Layla: Well, about a year ago I just started… wanting sex less. I’m not sure why. Before that things were fine. When I think about it… I mean, I did start a new job about a year ago as well, and there is some stress there, but I don’t think that explains everything.

Dr.: Any other changes you’ve noticed? Like do you have pain with sex, or trouble reaching orgasm?

Layla: Hmm, no pain, really… It does take me longer to get to orgasm than it used to, but I can. It’s just sometimes my mind is distracted while we’re trying to have sex.

Dr.: How has your mood been with this going on?

Layla: Well, of course it’s frustrating, but in general I feel pretty good.

Dr.: And how are you managing the job stress?

Layla: I like to run on the treadmill after I get home from work. That is a real stress reliever for me.

Dr.: And how are things with Connor:

Layla: Oh he is so sweet. He wants to try to help me with this thing, but he doesn’t know how. We’ve tried doing a date night once a week, and while that’s nice, it doesn’t necessarily make me more interested in sex, you know?

Dr.: So it seems like you’re able to talk about the change in your libido.

Layla: Yes we are.

*Pause for reflection – What did you notice?*

Dr.: That’s good to hear. Sometimes the way we were raised can have an effect on our sexuality as we get older. Do you think that plays a role for you?

Layla: Hmm, I’m not really sure what you mean.

Dr.: Well some of my patients, their religious background, or beliefs in their family, can have an effect.

Layla: Ahh I see. I mean, we went to church on holidays, but I’m not too religious. My parents didn’t really talk about sex with me much.

Dr.: Ok. And are there any medical symptoms you are experiencing that we didn’t talk about?

Layla: I don’t think so. What would you be looking for?

Dr.: Perhaps changes in your weight, hot flashes, feeling tired, changes in your menstrual cycle?

Layla: Haha, well, I think everyone is tired, right? No, nothing major.

Dr.: Great, thanks. Now I’m going to go ahead and do a physical exam to see if we get any clues.

….

Dr.: Ok, everything looks healthy with regards to your physical exam. So we have a few options to tackle this problem you’re experiencing…

*Pause for reflection – What did you notice?*
