# Supplementary material for: Female Sexual Dysfunction: A Primer for Primary Care Health Professionals
Source: MedEdPORTAL. 2023 Apr 25;19:11312. doi: 10.15766/mep_2374-8265.11312 (PMC10126124; doi:10.15766/mep_2374-8265.11312)
Supplement: Supplementary file 1 — 60-Minute Didactic.pptx90-Minute Workshop.pptxDiscussion Cases.docxSexual Devices Language Drills.docxRole-Play Script.docxEvaluation.docx [file mep_2374-8265.11312-s001.zip › F. Evaluation.docx]

# Workshop evaluation

Please complete the survey below. All questions are optional. Thank you!

Which of the following is the gender that you most identify with?

- Woman
- Man
- Cisgender
- Transgender
- Genderqueer, Gender Fluid, and/or Agender
- Intersex
- Prefer not to answer
- Other
  - Please describe your gender (free response)

How long have you been in clinical practice since completing your training?

- 0-2 years
- 3-5 years
- 6-10 years
- 11-15 years
- > 15 years

What is your current academic rank? [check all that apply]

- Medical Student
- Resident
- APP (e.g., nurse practitioner or physician assistant)
- Fellow
- Clinical instructor
- Clinical assistant
- Clinical associate
- Instructor
- Assistant professor
- Associate professor
- Full professor
- Not Applicable
- Other
  - Please describe your current academic rank (free response)

What region of the country are you currently in practice?

- New England
- Mid-Atlantic
- South
- Midwest
- Northwest
- California-Hawaii
- Other
- If outside of the United States, please describe your current region or country of practice (free response)

Which of the following best describes your current practice setting? [check all that apply]

- Urban Academic center
- Suburban academic center
- Rural academic center
- Urban community center
- Suburban community center
- Rural community center
- Other
  - Please describe your current practice setting (free response)

Have you had prior training in female sexual dysfunction in the past?

- Yes
- No

How often do you discuss sexual dysfunction with female or LGBTQI patients?

- Never
- Less than 6 times per year
- Monthly
- Weekly
- Daily

How would you rate the quality of content of this workshop?

- Outstanding
- Above Average
- Average
- Below Average
- Poor

How would you rate the audiovisual materials and activities (slides, handouts, breakout activities, etc)?

- Outstanding
- Above Average
- Average
- Below Average
- Poor

What is your overall evaluation of this session?

- Outstanding
- Above Average
- Average
- Below Average
- Poor

Did this workshop change how you plan to discuss female sexual dysfunction with your patients?

- Yes
- No

What is one change that you plan to make in your clinical practice as a result of this workshop? (free response)

How can we improve this workshop for future sessions? (free response)

Do you have any additional comments? (free response)
